# Supplementary material for: Maximum-likelihood model fitting for quantitative analysis of SMLM data
Source: Nat Methods. 2022 Dec 15;20(1):139–48. doi: 10.1038/s41592-022-01676-z (PMC9834062; doi:10.1038/s41592-022-01676-z)
Supplement: Supplementary file 1 — Supplementary Tables 1–7 [file 41592_2022_1676_MOESM1_ESM.pdf]

nature methods

Article https://doi.org/10.1038/s41592-022-01676-z

Maximum-likelihood model fitting for quantitative analysis of SMLM data

In the format provided by the authors and unedited

Supplementary Information

Supplementary Table 1. Simulation parameters.

Parameter	Value*	Unit	Symbol
**Extended Data Figure 1 and Extended Data Figure 2:**			
**SMLM property**			
photon count	15000		
background	500	photons/pixel/localization	
labelling efficiency	60	percent	
re-blinks	2		
lifetime	2	frame	
linkage error	3	nm	
EM on	true		
number of frames	100000		
**NPC model (NPC_{m3}) parameter**			
x	[-20, 20]	nm	x_0
y	[-20, 20]	nm	y_0
z	[-20, 20]	nm	z_0
xrot	[-15, 15]	°	α
yrot	[-15, 15]	°	β
zrot	[0, 360]	°	γ
variation	0	nm	ϵ
weight	1		w_1
xscale	1		s_x
yscale	1		s_y
zscale	1		s_z
ringDistance	50	nm	s
radius	53.4	nm	r
cornerDegree	12	°	ψ
azimuthalShift	8.8	°	θ
background weight	0.1		w_{bg}
**Simulations in Fig. 2d-f, Extended Data Figure 5a-e, Extended Data Figure 7, and Extended Data Figure 10e:**			
**SMLM property**			
photon count	12000		
background	140	photons/pixel/localization	
labelling efficiency	67	percent	
re-activation	4.1		
lifetime	1	frame	
linkage error	6.4**	nm	
EM on	true		
number of frames	100000		
**NPC model (NPC_{m3}) parameter**			
x	[-50, 50]	nm	x_0
y	[-50, 50]	nm	y_0
z	[-50, 50]	nm	z_0
xrot	[-15, 15]	°	α
yrot	[-15, 15]	°	β
zrot	[0, 360]	°	γ
variation	0	nm	ϵ
weight	1		w_1
xscale	1		s_x
yscale	1		s_y
zscale	1		s_z
ringDistance	50.2	nm	s
radius	53.4	nm	r
cornerDegree	12	°	ψ
azimuthalShift	8.8	°	θ
background density	20	μm^{-2}	ρ_{bg}
**Extended Data Figure 6:**			
**SMLM property**			
photon count	12000		
background	140	photons/pixel/localization	
labelling efficiency	67	percent	

re-activation	4.1		
lifetime	1	frame	
linkage error	6.4	nm	
EM on	true		
number of frames	100000		
**Elliptical NPC model (NPC_e) parameter**			
x	[-50, 50]	nm	x_0
y	[-50, 50]	nm	y_0
z	[-50, 50]	nm	z_0
xrot	[-15, 15]	°	α
yrot	[-15, 15]	°	β
zrot	[0, 180]	°	γ
variation	0	nm	ϵ
weight	1		w_1
xscale	1		s_x
yscale	1		s_y
zscale	1		s_z
ringDistance	50.2	nm	s
avgR	53.4	nm	r
ellipticity	0.1		e
cornerDegree	12	°	ψ
azimuthalShift	8.8	°	θ
background density	20	μm^{-2}	ρ_{bg}

Simulations in Extended Data Figure 5f-h:

**SMLM property**			
photon count	8000		
background	63	photons/pixel/localization	
labelling efficiency	NA	percent	
re-activation	2		
lifetime	1	frame	
linkage error	4	nm	
EM on	true		
number of frames	100000		
**Microtubule model (MT_{m2}) parameter**			
x	0	nm	x_0
y	0	nm	y_0
z	0	nm	z_0
xrot	0	°	α
yrot	0	°	β
zrot	0	°	γ
variation	0	nm	ϵ
weight	1		w_1
xscale	1		s_x
yscale	1		s_y
zscale	1		s_z
xMid	0	nm	x_{mid}
yMid	0	nm	y_{mid}
zMid	0	nm	z_{mid}
x_n	0, 1200, 2400, 3600, 4800, 6000, 7200	nm	x_{n}
y_n	0, -600, 0, 600, 0, -600, 0	nm	y_{n}
z_n	50, 25, 0, -25, 50, -25, 0	nm	z_{n}
background density	40	μm^{-2}	ρ_{bg}
number of molecules	$a \times 540$***		

Simulations in Extended Data Figure 8:

**SMLM property**			
photon count	12000		
background	140	photons/pixel/localization	
labelling efficiency	67	percent	
re-activation	4.1		
lifetime	1	frame	
linkage error (free)	4	nm	
EM on	true		
number of frames	100000		
**Line segment model (LS) parameter**			
x	0	nm	x_0
y	0	nm	y_0
z	0	nm	z_0
xrot	0	°	α
yrot	0	°	β

zrot	0	°	γ
variation	0	nm	ϵ
weight	1		w_1
xscale	1		s_x
yscale	1		s_y
zscale	1		s_z
xMid	0	nm	x_{mid}
yMid	0	nm	y_{mid}
zMid	0	nm	z_{mid}
dist	$300/(N-1)$****	nm	h
rotAzi_q	[-60 60]	°	θ_q
rotEle_q	[-60 60]	°	φ_q
background density	20	μm^{-2}	ρ_{bg}
number of molecules	$N \times 20$****		

* These values were used unless indicated otherwise.
** 4 nm was used for Extended Data Figure 10e.
*** a is the arclength in µm.
**** N is the number of vertices.

Supplementary Table 2. Fitting settings used in this study for Nup96 in single color data.

Step	Model	Parameter type	Internal parameter name	Free parameter	Relative LB	Relative UB	Absolute LB	Absolute UB	Initial value	Unit	Symbol
1	1	e	x	yes	-50	50	-150	150	$\tilde{x}_k$	nm	x_0
1	1	e	y	yes	-50	50	-150	150	$\tilde{y}_k$	nm	y_0
1	1	e	z	yes	-100	20	-300	300	$\tilde{z}_k - 40$	nm	z_0
1	1	e	weight	no					1		w_1
1	1	e	xrot	yes	-30	30	-inf	inf	0	°	α
1	1	e	yrot	yes	-30	30	-inf	inf	0	°	β
1	1	e	zrot	no					0	°	γ
1	1	e	xscale	no					1		s_x
1	1	e	yscale	no					1		s_y
1	1	e	zscale	no					1		s_z
1	1	e	variation	no					0	nm	ϵ
1	2	e	x	no					0	nm	x_0
1	2	e	y	no					0	nm	y_0
1	2	e	z	yes	-30	100	0	300	40	nm	$\hat{z}_0$
1	2	e	xrot	no					0	°	α
1	2	e	yrot	no					0	°	β
1	2	e	zrot	no					0	°	γ
1	2	e	variation	no					0	nm	ϵ
1	2	e	weight	no					1		w_1
1	2	e	xscale	no					0		s_x
1	2	e	yscale	no					0		s_y
1	2	e	zscale	no					0		s_z
1	L1	e	weight	yes	-1	1	0.001	0.999	0		w_{bg}
2	1	e	x	yes	-20	20	-150	150	$\hat{x}_0^{1,1} + \phi(\hat{z}_0^{1,2})_{[1]}$	nm	x_0
2	1	e	y	yes	-20	20	-150	150	$\hat{y}_0^{1,1} + \phi(\hat{z}_0^{1,2})_{[2]}$	nm	y_0
2	1	e	z	yes	-20	20	-300	300	$\hat{z}_0^{1,1} + \phi(\hat{z}_0^{1,2})_{[3]}$	nm	z_0
2	1	e	xrot	yes	-15	15	-inf	inf	inherited	°	α
2	1	e	yrot	yes	-15	15	-inf	inf	inherited	°	β
2	1	e	zrot	no					0	°	γ
2	1	e	variation	yes	-10	10	1	20	5	nm	ϵ
2	1	e	weight	no					1		w_1
2	1	e	xscale	no					0		s_x
2	1	e	yscale	no					0		s_y
2	1	e	zscale	no					0		s_z
2	1	i	azimuthalShift	no					0	°	θ
2	1	i	cornerDegree	no					12	°	ψ
2	1	i	ringDistance	yes	-10	10	0	100	inherited	nm	s
2	1	i	radius	yes	-10	10	30	70	inherited	nm	r
2	L1	e	weight	no					inherited		w_{bg}
3	1	e	x	no					inherited	nm	x_0
3	1	e	y	no					inherited	nm	y_0
3	1	e	z	no					inherited	nm	z_0
3	1	e	xrot	no					inherited	°	α
3	1	e	yrot	no					inherited	°	β
3	1	e	zrot	yes	-180	180	-inf	inf	0	°	r
3	1	e	variation	yes	-10	10	1	20	5	nm	ϵ
3	1	e	weight	no					inherited		w_1
3	1	e	xscale	no					inherited		s_x
3	1	e	yscale	no					inherited		s_y
3	1	e	zscale	no					inherited		s_z
3	1	i	ringDistance	no					inherited	nm	s
3	1	i	radius	no					inherited	nm	r
3	1	i	cornerDegree	no					12	°	ψ
3	1	i	azimuthalShift	yes	-180	180	-inf	inf	0	°	θ
3	L1	e	weight	yes	-1	1	0.001	0.999	inherited		w_{bg}

Note: In column *Parameter type*, i and e represent intrinsic and extrinsic parameters, respectively. In column *Model*, L1 represents layer 1. In column *initial value*, a tilde operator represents a median value, a hat operator indicates a parameter estimate, and superscripts represent steps and models (e.g., $\hat{z}_0^{1,2}$ means the estimate of z_0 from model 2 in step 1). The function $\vec{z}'_{[k]} = \phi(z)_{[k]}$ outputs the kth element of the vector $\vec{z}'$ derived from $\vec{z} = [0 \quad 0 \quad z]$ rotated by the rotation matrix constructed using $\{\hat{\alpha}^{1,1}, \hat{\beta}^{1,1}, \hat{r}^{1,1}\}$ as the angles $\{\alpha, \beta, \gamma\}$ in the equation (10). 'inherited' indicates that a value is inherited from its counterpart estimate in the last step. Other symbols have the same meanings as in the main text. Abbreviations: LB/UB: lower/upper boundaries. Blanks indicate values not applicable.

Supplementary Table 3. Fitting settings used in this study for Nup96 in dual-color data.

Step	Model	Parameter type	Internal parameter name	Free parameter	Relative LB	Relative UB	Absolute LB	Absolute UB	Initial value	Unit	Symbol
1	1	e	x	yes	-50	50	-150	150	$\tilde{x}_k$	nm	x_0
1	1	e	y	yes	-50	50	-150	150	$\tilde{y}_k$	nm	y_0
1	1	e	z	yes	-100	20	-300	300	$\tilde{z}_k - 40$	nm	z_0
1	1	e	weight	no					1		w_1
1	1	e	xrot	yes	-30	30	-inf	inf	0	º	α
1	1	e	yrot	yes	-30	30	-inf	inf	0	º	β
1	1	e	zrot	no					0	º	γ
1	1	e	xscale	no					1		s_x
1	1	e	yscale	no					1		s_y
1	1	e	zscale	no					1		s_z
1	1	e	variation	no					0	nm	ϵ
1	2	e	x	no					0	nm	x_0
1	2	e	y	no					0	nm	y_0
1	2	e	z	yes	-30	100	0	300	40	nm	z_0
1	2	e	xrot	no					0	º	α
1	2	e	yrot	no					0	º	β
1	2	e	zrot	no					0	º	γ
1	2	e	variation	no					0	nm	ϵ
1	2	e	weight	no					1		w_1
1	2	e	xscale	no					0		s_x
1	2	e	yscale	no					0		s_y
1	2	e	zscale	no					0		s_z
1	L1	e	weight	yes	-1	1	0.001	0.999	0		w_{bg}
2	1	e	x	yes	-20	20	-150	150	$\hat{x}_0^{1,1} + \phi(\hat{z}_0^{1,2})_{[1]}$	nm	x_0
2	1	e	y	yes	-20	20	-150	150	$\hat{y}_0^{1,1} + \phi(\hat{z}_0^{1,2})_{[2]}$	nm	y_0
2	1	e	z	yes	-20	20	-300	300	$\hat{z}_0^{1,1} + \phi(\hat{z}_0^{1,2})_{[3]}$	nm	z_0
2	1	e	xrot	yes	-10	10	-Inf	Inf	inherited	º	α
2	1	e	yrot	yes	-10	10	-Inf	Inf	inherited	º	β
2	1	e	zrot	yes	-180	180	-inf	inf	[1 360]	º	r
2	1	e	variation	yes	-10	10	1	20	5	nm	ϵ
2	1	e	weight	no					1		w_1
2	1	e	xscale	no					1		s_x
2	1	e	yscale	no					1		s_y
2	1	e	zscale	no					1		s_z
2	1	i	ringDistance	no					50.2	nm	s
2	1	i	radius	no					53.4	nm	r
2	1	i	cornerDegree	no					12	º	ψ
2	1	i	azimuthalShift	no					8.8	º	θ
2	L1	e	weight	yes	-1	1	0.001	0.999	inherited		w_{bg}

Note: In column *Parameter type*, i and e represent intrinsic and extrinsic parameters, respectively. In column *Model*, L1 represents layer 1. In column *initial value*, a tilde operator represents a median value, a hat operator indicates a parameter estimate, and superscripts represent steps and models (e.g., $\hat{z}_0^{1,2}$ means the estimate of z_0 from model 2 in step 1). The function $\vec{z}'_{[k]} = \phi(z)_{[k]}$ outputs the kth element of the vector $\vec{z}'$ derived from $\vec{z} = [0 \quad 0 \quad z]$ rotated by the rotation matrix constructed using $\{\hat{\alpha}^{1,1}, \hat{\beta}^{1,1}, \hat{r}^{1,1}\}$ as the angles $\{\alpha, \beta, \gamma\}$ in the equation (10). 'inherited' indicates that a value is inherited from its counterpart estimate in the last step. Other symbols have the same meanings as in the main text. Abbreviations: LB/UB: lower/upper boundaries. Blanks indicate values not applicable.

Supplementary Table 4. Fitting settings used in this study for micrometer-long microtubule segments.

Step	Model	Parameter type	Internal parameter name	Free parameter	Relative		Absolute		Initial value	Unit	Symbol
					LB	UB	LB	UB			
1	1	e	x	no					$\tilde{x}_k$	nm	x_0
1	1	e	y	no					$\tilde{y}_k$	nm	y_0
1	1	e	z	no					$\tilde{z}_k$	nm	z_0
1	1	e	xrot	no					0	°	α
1	1	e	yrot	no					0	°	β
1	1	e	zrot	no					$pcaRot(\tilde{x}_k, \tilde{y}_k,)$	°	γ
1	1	e	variation	yes	-Inf	Inf	30	45	40	nm	ϵ
1	1	e	weight	no					1		w_1
1	1	e	xscale	no					1		s_x
1	1	e	yscale	no					1		s_y
1	1	e	zscale	no					1		s_z
1	1	i	xMid	yes	-Inf	Inf	-500	500	0	nm	x_{mid}
1	1	i	yMid	yes	-Inf	Inf	-500	500	0	nm	y_{mid}
1	1	i	zMid	yes	-Inf	Inf	-500	500	0	nm	z_{mid}
1	1	i	dist	no					250	nm	h
1	1	i	rotAzi_q	yes	-25	25	-Inf	Inf	0	°	θ_q
1	1	i	rotEle_q	yes	-25	25	-Inf	Inf	0	°	φ_q
1	L1	e	weight	yes	-1	1	0.001	0.999	0.5		w_{bg}
2	1	e	x	no					inherited	nm	x_0
2	1	e	y	no					inherited	nm	y_0
2	1	e	z	no					inherited	nm	z_0
2	1	e	xrot	no					inherited	°	α
2	1	e	yrot	no					inherited	°	β
2	1	e	zrot	no					inherited	°	γ
2	1	e	variation	no					7	nm	ϵ
2	1	e	weight	no					inherited		w_1
2	1	e	xscale	no					inherited		s_x
2	1	e	yscale	no					inherited		s_y
2	1	e	zscale	no					inherited		s_z
2	1	i	xMid	yes	-30	30	-500	500	inherited	nm	x_{mid}
2	1	i	yMid	yes	-30	30	-500	500	inherited	nm	y_{mid}
2	1	i	zMid	yes	-30	30	-500	500	inherited	nm	z_{mid}
2	1	i	r	yes	-Inf	Inf	10	60	30	nm	r
2	1	i	dist	no					inherited	nm	h
2	1	i	rotAzi_q	yes	-25	25	-Inf	Inf	0	°	θ_q
2	1	i	rotEle_q	yes	-25	25	-Inf	Inf	0	°	φ_q
2	L1	e	weight	yes	-1	1	0.001	0.999	inherited		w_{bg}

Note: In column *Parameter type*, i and e represent intrinsic and extrinsic parameters, respectively. In column *Model*, L1 represents layer 1. In column *initial value*, a tilde operator represents a median value. The function $pcaRot(x_k, y_k,)$ outputs the angle between the x-axis and the first component of the localizations in the x-y plane. 'inherited' indicates that a value is inherited from its counterpart estimate in the last step. Other symbols have the same meanings as in the main text. Abbreviations: LB/UB: lower/upper boundaries. Blanks indicate values not applicable.

Supplementary Table 5. Fitting settings used in this study for long microtubule segments.

Step	Model	Parameter type	Internal parameter name	Free parameter	Relative		Absolute		Initial value	Unit	Symbol
					LB	UB	LB	UB			
1	1	e	x	no					$\tilde{x}_k$	nm	x_0
1	1	e	y	no					$\tilde{y}_k$	nm	y_0
1	1	e	z	no					$\tilde{z}_k$	nm	z_0
1	1	e	xrot	no					0	°	α
1	1	e	yrot	no					0	°	β
1	1	e	zrot	no					$pcaRot(\tilde{x}_k, \tilde{y}_k,)$	°	γ
1	1	e	variation	yes	-Inf	Inf	20	1000	1000	nm	ϵ
1	1	e	weight	no					1		w_1
1	1	e	xscale	no					1		s_x
1	1	e	yscale	no					1		s_y
1	1	e	zscale	no					1		s_z
1	1	i	xMid	yes	-Inf	Inf	-2500	2500	0	nm	x_{mid}
1	1	i	yMid	yes	-Inf	Inf	-2500	2500	0	nm	y_{mid}
1	1	i	zMid	yes	-Inf	Inf	-500	500	0	nm	z_{mid}
1	1	i	dist	no					200	nm	h
1	1	i	rotAzi_q	yes	-20	20	-Inf	Inf	0	°	θ_q
1	1	i	rotEle_q	yes	-15	15	-Inf	Inf	0	°	φ_q
1	L1	e	weight	yes	-1	1	0.001	0.999	0.5		w_{bg}
2	1	e	x	no					inherited	nm	x_0
2	1	e	y	no					inherited	nm	y_0
2	1	e	z	no					inherited	nm	z_0
2	1	e	xrot	no					inherited	°	α
2	1	e	yrot	no					inherited	°	β
2	1	e	zrot	no					inherited	°	γ
2	1	e	variation	yes	-Inf	Inf	20	40	40	nm	ϵ
2	1	e	weight	no					inherited		w_1
2	1	e	xscale	no					inherited		s_x
2	1	e	yscale	no					inherited		s_y
2	1	e	zscale	no					inherited		s_z
2	1	i	xMid	yes	-Inf	Inf	-2500	2500	inherited	nm	x_{mid}
2	1	i	yMid	yes	-Inf	Inf	-2500	2500	inherited	nm	y_{mid}
2	1	i	zMid	yes	-Inf	Inf	-500	500	inherited	nm	z_{mid}
2	1	i	dist	no					inherited	nm	u
2	1	i	rotAzi_q	yes	-Inf	Inf	-20	20	0	°	θ_q
2	1	i	rotEle_q	yes	-Inf	Inf	-15	15	0	°	φ_q
2	L1	e	weight	yes	-1	1	0.001	0.999	inherited		w_{bg}
3	1	e	x	no					inherited	nm	x_0
3	1	e	y	no					inherited	nm	y_0
3	1	e	z	no					inherited	nm	z_0
3	1	e	xrot	no					inherited	°	α
3	1	e	yrot	no					inherited	°	β
3	1	e	zrot	no					inherited	°	γ
3	1	e	variation	no					7	nm	ϵ
3	1	e	weight	no					inherited		w_1
3	1	e	xscale	no					inherited		s_x
3	1	e	yscale	no					inherited		s_y
3	1	e	zscale	no					inherited		s_z
3	1	i	xMid	yes	-30	30	-Inf	Inf	inherited	nm	x_{mid}
3	1	i	yMid	yes	-30	30	-Inf	Inf	inherited	nm	y_{mid}
3	1	i	zMid	yes	-30	30	-Inf	Inf	inherited	nm	z_{mid}
3	1	i	r	yes	-Inf	Inf	10	60	30	nm	r
3	1	i	dist	no					inherited	nm	h
3	1	i	rotAzi_q	yes	-Inf	Inf	-20	20	0	°	θ_q
3	1	i	rotEle_q	yes	-Inf	Inf	-15	15	0	°	φ_q
3	L1	e	weight	yes	-1	1	0.001	0.999	inherited		w_{bg}

Note: In column *Parameter type*, i and e represent intrinsic and extrinsic parameters, respectively. In column *Model*, L1 represents layer 1. In column *initial value*, a tilde operator represents a median value. The function $pcaRot(x_k, y_k,)$ outputs the angle between the x-axis and the first component of the localizations in the x-y plane. 'inherited' indicates that a value is inherited from its counterpart estimate in the last step. Other symbols have the same meanings as in the main text. Abbreviations: LB/UB: lower/upper boundaries. Blanks indicate values not applicable.

Supplementary Table 6. Fitting settings used in this study for endocytic sites in yeast.

Step	Model	Parameter type	Internal parameter name	Free parameter	Relative		Absolute		Initial value	Unit	Symbol
					LB	UB	LB	UB			
1	1	e	x	yes	-30	30	-150	150	0	nm	x_0
1	1	e	y	yes	-50	50	-150	150	$\tilde{y}_k^1$	nm	y_0
1	1	e	zrot	yes	-15	15	-Inf	Inf	0	º	γ
1	1	e	variation	no					0	nm	ϵ
1	1	e	weight	no					1		w_1
1	1	e	xscale	no					1		s_x
1	1	e	yscale	no					1		s_y
1	1	i	innerRadius	no					40	nm	r
1	1	i	outerRadius	no					70	nm	q
1	1	i	thickness	no					60	nm	t
1	2	e	x	no					0	nm	x_0
1	2	e	y	yes	-300	50	-200	200	0	nm	y_0
1	2	e	zrot	no					0	º	γ
1	2	e	variation	no					0	nm	ϵ
1	2	e	weight	no					1		w_1
1	2	e	xscale	no					0		s_x
1	2	e	yscale	no					0		s_y
1	2	i	a	no					85	nm	a
1	2	i	b	yes	0	250	1	270	0	nm	b
1	L1	e	weight	yes	-1	1	0.001	0.999	0		w_{bg}
1	L2	e	weight	yes	-1	1	0.001	0.999	0		w_{bg}

Note: In column Parameter type, i and e represent intrinsic and extrinsic parameters, respectively. In column Model, L1 and L2 represent layer 1 and layer 2. In column initial value, a tilde operator represents a median value, a hat operator indicates a parameter estimate, and superscripts represent channels (e.g., $\tilde{y}_k^1$ means the median of y_k, or y positions of localizations, in channel 1). Other symbols have the same meanings as in the main text. Abbreviations: LB/UB: lower/upper boundaries. Blanks indicate values not applicable.

Supplementary Table 7. Fitting settings used in this study for line segments.

Step	Model	Parameter type	Internal parameter name	Free parameter	Relative		Absolute		Initial value	Unit	Symbol
					LB	UB	LB	UB			
1	1	e	x	no					$\tilde{x}_k$	nm	x_0
1	1	e	y	no					$\tilde{y}_k$	nm	y_0
1	1	e	z	no					$\tilde{z}_k$	nm	z_0
1	1	e	xrot	no					0	º	α
1	1	e	yrot	no					0	º	β
1	1	e	zrot	yes	-360	360	-Inf	Inf	$pcaRot(\tilde{x}_k, \tilde{y}_k,)$	º	γ
1	1	e	variation	yes	-Inf	Inf	10	100	50	nm	ϵ
1	1	e	weight	no					1		w_1
1	1	e	xscale	no					1		s_x
1	1	e	yscale	no					1		s_y
1	1	e	zscale	no					1		s_z
1	1	i	xMid	yes	-30	-30	-200	200	0	nm	x_{mid}
1	1	i	yMid	yes	-30	-30	-200	200	0	nm	y_{mid}
1	1	i	zMid	yes	-30	-30	-200	200	0	nm	z_{mid}
1	1	i	dist	no					$300/(N-1)$	nm	h
1	1	i	rotAzi_q	yes	-60	20	-Inf	Inf	0	º	θ_q
1	1	i	rotEle_q	yes	-15	15	-Inf	Inf	0	º	φ_q
1	L1	e	weight	yes	-1	1	0.001	0.999	0.5		w_{bg}

Note: In column *Parameter type*, i and e represent intrinsic and extrinsic parameters, respectively. In column *Model*, L1 represents layer 1. In column *initial value*, a tilde operator represents a median value. The function $pcaRot(x_k, y_k,)$ outputs the angle between the x-axis and the first component of the localizations in the x-y plane. N is the number of vertices. 'inherited' indicates that a value is inherited from its counterpart estimate in the last step. Other symbols have the same meanings as in the main text. Abbreviations: LB/UB: lower/upper boundaries. Blanks indicate values not applicable.
