# Supplementary material for: Maximum-likelihood model fitting for quantitative analysis of SMLM data
Source: Nat Methods. 2022 Dec 15;20(1):139–48. doi: 10.1038/s41592-022-01676-z (PMC9834062; doi:10.1038/s41592-022-01676-z)
Supplement: Supplementary file 2 — Reporting Summary [file 41592_2022_1676_MOESM2_ESM.pdf]

nature portfolio

Corresponding author(s): Jonas Ries

Last updated by author(s): Nov 22, 2022

Reporting Summary

Nature Portfolio wishes to improve the reproducibility of the work that we publish. This form provides structure for consistency and transparency in reporting. For further information on Nature Portfolio policies, see our Editorial Policies and the Editorial Policy Checklist.

Statistics

For all statistical analyses, confirm that the following items are present in the figure legend, table legend, main text, or Methods section.

n/a	Confirmed	
☐	☒	The exact sample size (*n*) for each experimental group/condition, given as a discrete number and unit of measurement
☐	☒	A statement on whether measurements were taken from distinct samples or whether the same sample was measured repeatedly
☒	☐	The statistical test(s) used AND whether they are one- or two-sided<br>*Only common tests should be described solely by name; describe more complex techniques in the Methods section.*
☒	☐	A description of all covariates tested
☐	☒	A description of any assumptions or corrections, such as tests of normality and adjustment for multiple comparisons
☐	☒	A full description of the statistical parameters including central tendency (e.g. means) or other basic estimates (e.g. regression coefficient) AND variation (e.g. standard deviation) or associated estimates of uncertainty (e.g. confidence intervals)
☒	☐	For null hypothesis testing, the test statistic (e.g. *F*, *t*, *r*) with confidence intervals, effect sizes, degrees of freedom and *P* value noted<br>*Give P values as exact values whenever suitable.*
☒	☐	For Bayesian analysis, information on the choice of priors and Markov chain Monte Carlo settings
☒	☐	For hierarchical and complex designs, identification of the appropriate level for tests and full reporting of outcomes
☒	☐	Estimates of effect sizes (e.g. Cohen's *d*, Pearson's *r*), indicating how they were calculated

Our web collection on statistics for biologists contains articles on many of the points above.

Software and code

Policy information about availability of computer code

Data collection	Micro-Manager (1.4 and 2.0) and our published software EMU (v1.1)
Data analysis	Matlab (2022a) and custom software available at https://github.com/jries/SMAP

For manuscripts utilizing custom algorithms or software that are central to the research but not yet described in published literature, software must be made available to editors and reviewers. We strongly encourage code deposition in a community repository (e.g. GitHub). See the Nature Portfolio guidelines for submitting code & software for further information.

Data

Policy information about availability of data

All manuscripts must include a data availability statement. This statement should provide the following information, where applicable:

- Accession codes, unique identifiers, or web links for publicly available datasets
- A description of any restrictions on data availability
- For clinical datasets or third party data, please ensure that the statement adheres to our policy

All image data used in this study are available on the BioImage Archive data repository (accession number: S-BIAD563).

Field-specific reporting

Please select the one below that is the best fit for your research. If you are not sure, read the appropriate sections before making your selection.

☒ Life sciences ☐ Behavioural & social sciences ☐ Ecological, evolutionary & environmental sciences

For a reference copy of the document with all sections, see nature.com/documents/nr-reporting-summary-flat.pdf

Life sciences study design

All studies must disclose on these points even when the disclosure is negative.

Sample size	We did not perform sample-size calculation. The current sample size is considered sufficient because it already provides higher precision than technical accuracy. Except for the long microtubule segments (for each condition, one segment is used for demonstration), the smallest sample size in this work is 161 and most of experiments/conditions have sample sizes larger than 1,000, resulting in well-defined distributions or average images.
Data exclusions	Only nuclear pore complexes with low quality or with ring separation smaller than 35 nm were excluded from further analyses. The exclusion was necessary for precise measurements of parameters. The rationale for this exclusion was shown in Extended Data Figure 3 and Extended Data Figure 4f-i.
Replication	All attempts at replication were successful. Each analysis was performed on three different cells except for the microtubule data shown in Figure 2. The original microtubule data was from Speiser et al., 2021., where only one cell was acquired.
Randomization	We did not perform any randomization. Randomization is not relevant because no comparison of different biological conditions was performed in this work.
Blinding	We did not perform any blinding. Blinding is not relevant because no comparison of different biological conditions was performed in this work.

Reporting for specific materials, systems and methods

We require information from authors about some types of materials, experimental systems and methods used in many studies. Here, indicate whether each material, system or method listed is relevant to your study. If you are not sure if a list item applies to your research, read the appropriate section before selecting a response.

Materials & experimental systems

n/a	Involved in the study	
☐	☒	Antibodies
☐	☒	Eukaryotic cell lines
☒	☐	Palaeontology and archaeology
☒	☐	Animals and other organisms
☒	☐	Human research participants
☒	☐	Clinical data
☒	☐	Dual use research of concern

Methods

n/a	Involved in the study	
☒	☐	ChIP-seq
☒	☐	Flow cytometry
☒	☐	MRI-based neuroimaging

Antibodies

Antibodies used	Primary:<br>Anti-Elys (catalog no. HPA031658, Atlas Antibodies, 1:50),<br>Anti-Nup133 (catalog no. HPA059767, Atlas Antibodies, 1:150),<br>Anti-Nup62 (catalog no. 610498, BD Biosciences, 1:150),<br>Anti-Nup153 (catalog no. ab24700, Abcam, 1:60)) .<br>Secondary:<br>CF660C labeled anti-rabbit antibody (catalog no. 20813, Biotium),<br>CF660C anti-mouse antibody (catalog no. 20815, Biotium),<br>CF680 labeled anti-rabbit antibody (catalog no. 20818, Biotium),<br>CF680 anti-mouse antibody (catalog no. 20819, Biotium).
Validation	Validations were performed by the respectively indicated manufacturers. The validated applications of the antibodies are summarized bellow: Anti-Elys for immunohistochemistry (IHC) and immunofluorescence in cell lines (ICC-IF); Anti-Nup133 for IHC, western blot (WB), and ICC-IF; Anti-Nup62 for WB (routinely tested), IF, immunoprecipitation (tested during development), and IHC (not recommended); Anti-Nup153 for ICC-IF. These antibodies were used to perform immunofluorescence staining on specimens for demonstrating the described analysis software in the work.

Eukaryotic cell lines

Policy information about cell lines

Cell line source(s)	U-2 OS Nup96-SNAP-tag (catalog no. 300444, CLS Cell Line Service, Eppelheim, Germany).
Authentication	None of cell lines were further authenticated.
Mycoplasma contamination	Cells were tested negative for mycoplasma contamination.
Commonly misidentified lines (See ICLAC register)	No commonly misidentified cell lines were used.
