# Supplementary material for: Maximum-likelihood model fitting for quantitative analysis of SMLM data
Source: Nat Methods. 2022 Dec 15;20(1):139–48. doi: 10.1038/s41592-022-01676-z (PMC9834062; doi:10.1038/s41592-022-01676-z)
Supplement: Supplementary file 7 — Source code of LocMoFit v1.1 [file 41592_2022_1676_MOESM7_ESM.zip › LocMoFit/external/PolyfitnTools/demo/html/polyfitn_demo.html]

polyfitn\_demo


 


## Contents

- Fit a 1-d model to cos(x). We only need the even order terms.
- A surface model in 2-d, with all terms up to third order.
- A linear model, but with no constant term, in 2-d
- A model with various exponents, not all positive integers.

```
% Author: John D'Errico
% Release: 2.0
% Release date: 8/8/06
% What follows are example usages of polyfitn, polyvaln, poly2sympoly, polyn2sym
```

## Fit a 1-d model to cos(x). We only need the even order terms.

```
x = -2:.1:2;
y = cos(x);
p = polyfitn(x,y,'constant x^2 x^4 x^6')

if exist('sympoly') == 2
  % Conversion to a sympoly. If nothing else, its a nice way to display the model.
  polyn2sympoly(p)
end
if exist('sym') == 2
  % Conversion to a symbolic form. Its also nice.
  polyn2sym(p)
end

% Evaluate the regression model at some set of points
polyvaln(p,[0 .5 1])
```

```
p = 

      ModelTerms: [4x1 double]
    Coefficients: [0.99996 -0.49968 0.041242 -0.0012079]
    ParameterVar: [1.2876e-10 1.084e-09 4.6603e-10 1.3903e-11]
    ParameterStd: [1.1347e-05 3.2925e-05 2.1588e-05 3.7286e-06]
              R2: 1
            RMSE: 3.1468e-05
        VarNames: {'x'}

A scalar sympoly object
    0.99996 - 0.49968*x^2 + 0.041242*x^4 - 0.0012079*x^6

ans =

      0.99996
       0.8776
      0.54031
```

## A surface model in 2-d, with all terms up to third order.

```
% Use lots of data.
n = 1000;
x = rand(n,2);
y = exp(sum(x,2)) + randn(n,1)/100;
p = polyfitn(x,y,3)

if exist('sympoly') == 2
  polyn2sympoly(p)
end
if exist('sym') == 2
  polyn2sym(p)
end

% Evaluate on a grid and plot:
[xg,yg]=meshgrid(0:.05:1);
zg = polyvaln(p,[xg(:),yg(:)]);
surf(xg,yg,reshape(zg,size(xg)))
hold on
plot3(x(:,1),x(:,2),y,'o')
hold off
```

```
p = 

      ModelTerms: [10x2 double]
    Coefficients: [1x10 double]
    ParameterVar: [1x10 double]
    ParameterStd: [1x10 double]
              R2: 0.99992
            RMSE: 0.011198
        VarNames: {}

A scalar sympoly object
    0.50402*X1^3 + 1.3927*X1^2*X2 - 0.017552*X1^2 + 1.3798*X1*X2^2 + 0.081473*X1*X2 + 1.2726*X1 + 0.43959*X2^3 + 0.087656*X2^2 + 1.2254*X2 + 0.96196
```

## A linear model, but with no constant term, in 2-d

```
uv = rand(100,2);
w = sin(sum(uv,2));
p = polyfitn(uv,w,'u, v');
if exist('sympoly') == 2
  polyn2sympoly(p)
end
if exist('sym') == 2
  polyn2sym(p)
end
```

```
A scalar sympoly object
    0.76416*u + 0.70472*v
```

## A model with various exponents, not all positive integers.

```
% Note: with only 1 variable, x & y may be row or column vectors.
x = 1:10;
y = 3 + 2./x + sqrt(x) + randn(size(x))/100;
p = polyfitn(x,y,'constant x^-1 x^0.5');
if exist('sympoly') == 2
  polyn2sympoly(p)
end
if exist('sym') == 2
  polyn2sym(p)
end

xi = 1:.1:10;
yi = polyvaln(p,xi);
plot(x,y,'ro',xi,yi,'b-')
```

```
A scalar sympoly object
    2.9805 + 2.0448*x^-1 + 1.0041*x^0.5
```

Published with MATLAB® 7.0.1
