# Supplementary material for: Maximum-likelihood model fitting for quantitative analysis of SMLM data
Source: Nat Methods. 2022 Dec 15;20(1):139–48. doi: 10.1038/s41592-022-01676-z (PMC9834062; doi:10.1038/s41592-022-01676-z)
Supplement: Supplementary file 7 — Source code of LocMoFit v1.1 [file 41592_2022_1676_MOESM7_ESM.zip › LocMoFit/external/PolyfitnTools/doc/understanding_polyfitn.rtf]

Understanding PolyfitnJohn R. D'Erricowoodchips@rochester.rr.com8/8/06Regression modelingPolyfitn is really rather simple. It solves for the coefficients of a polynomial regression model using traditional linear least squares techniques, with some care applied in the solution. The method chosen for its solution is one that also yields standard errors for the coefficients.A good reference for regression analysis is the one I learned from:Draper, N.R., Smith, H.; "Applied Regression Analysis"; John Wiley & SonsCare has been taken in the numerical methods of polyfitn, first by use of a QR factorization with pivoting to solve the system. This is more stable than the simple, unpivoted QR. I've also used automatic variable scaling to deal with a simple cause of ill-conditioning.Numerical details of polyfitnOnce the polynomial (or multinomial in n-dimensions) model has been specified, the estimation procedure is itself rather simple. The problem becomes that of estimation of the vector x, given the linear system of equations	A*x = yOf course, for this estimation to have a unique solution, the matrix A should be both nonsingular and have more rows than columns. Problems with fewer rows than columns are called underdetermined. I'd strongly recommend that you get more data if there are fewer data points than parameters to estimate.So, assuming that A is an nxp matrix, with n>p, we can solve this system via many different approaches in Matlab. I'll outline the method I chose, as well as what I've done to assure the the best parameter estimates possible.The traditional methods in Matlab that one might use to estimate a linear regression model are- Backslash (\)- PINV- LSQR- The normal equations, i.e., x = (A' * A)\(A' * y)- QR decomposition- Pivoted QR decompositionOf these methods, only those based on the QR factorization will also directly yield estimated variances for the parameters. A pivoted QR is also reasonably efficient, as well as numerically stable, so I chose this method.Given the design matrix A in the above expression, the first task is to scale it so that all columns have unit mean. While I'd like to introduce a shift also for the best possible estimates, this is not possible for a general multinomial model, as it may introduce additional terms into the model. For example, suppose the polynomial model we must estimate is	y = a1*x + a2*x^2If we translate the columns by automatically subtracting the mean, then we have implicitly inserted a constant term into the model which was not requested. So all that is done is a scaling of the matrix A. Essentially, we solve the problem	A*D*inv(D)*x = yHere D is the diagonal matrix	D = diag(1./mean(A,1));If we write	z = inv(D)*xthen the scaling results in the problem	(A*D)*z = ySolve for z, then scale back to recover x.The next problem to worry about is the existence of nearly linearly dependent columns in A. I'll use an extreme example to illustrate what might happen otherwise. Consider an under-determined linear regression problem:	n = 50;	X = randn(n,3);	X = X(:,[1 2 2 3]);Note that the third column is actually just a copy of the second column. Now, generate Y as the simple sum of columns [1 2 4] of X.	Y = X*[1;1;0;1] + randn(n,1)/100000;The coefficients of our true model are just	Y = 1*x1 + 1*x2 + 0*x3 + 1*x4Can we estimate Details of the arguments for polyfitnThere are only three arguments to polyfitn.- An array (or vector) of independent variable values. Each column of this array corresponds to an independent variable. If a vector, - A vector of dependent variable values.- A model specification
