# Supplementary material for: Maximum-likelihood model fitting for quantitative analysis of SMLM data
Source: Nat Methods. 2022 Dec 15;20(1):139–48. doi: 10.1038/s41592-022-01676-z (PMC9834062; doi:10.1038/s41592-022-01676-z)
Supplement: Supplementary file 7 — Source code of LocMoFit v1.1 [file 41592_2022_1676_MOESM7_ESM.zip › LocMoFit/external/PolyfitnTools/ReadMe.rtf]

ReadMe - PolyfitnJohn D'Erricowoodchips@rochester.rr.com8/23/06This directory contains several functions. The main one of course is polyfitn.m, which is why I've posted it all.POLYFITN - A general n-dimensional polynomial fitting toolPOLYVALN - An evaluation tool for polynomials produced by polyfitnPOLYN2SYMPOLY - A conversion tool to generate a sympoly from the results of polyfitnPOLYN2SYM - A conversion tool to generate a symbolic toolbox object from the results of polyfitnNote that polyn2sympoly is only of value IF you also have downloaded my sympoly toolbox. This will allow you to display and manipulate the resultant polynomials symbolically. You can find it from this link:http://www.mathworks.com/matlabcentral/fileexchange/loadFile.do?objectId=9577&objectType=FILELikewise, polyn2sym needs the symbolic toolbox to function.In additions to these m-files, there are several other files of interest. For those who have an interest in understanding how polyfitn works (quite simple really) you should look to the file understanding_polyfitn.rtf, in the doc subdirectory.
