# Supplementary material for: Maximum-likelihood model fitting for quantitative analysis of SMLM data
Source: Nat Methods. 2022 Dec 15;20(1):139–48. doi: 10.1038/s41592-022-01676-z (PMC9834062; doi:10.1038/s41592-022-01676-z)
Supplement: Supplementary file 7 — Source code of LocMoFit v1.1 [file 41592_2022_1676_MOESM7_ESM.zip › LocMoFit_manual.pdf]

LocMoFit (Localization Model Fit)

Release 1.1.0

Yu-Le Wu

For a better reading experience, please use the online version: https://locmofit.readthedocs.io/en/latest/

Aug 08, 2022

CONTENTS

I Introduction 1

1 LocMoFit 3
- 1.1 Overview . . . 3
- 1.2 New to LocMoFit? . . . 3
- 1.3 Content navigation . . . 3

2 Getting started 5
- 2.1 Requirements . . . 5
- 2.2 Installation . . . 6
- 2.3 Using LocMoFit with GUI now (SMAP required) . . . 6

II Basics 7

3 Structure of LocMoFit 9
- 3.1 Geometric model . . . 9
- 3.2 SMLM model . . . 10
- 3.3 Fitter . . . 10

4 Graphic user interface (GUI) 11
- 4.1 GUI overview . . . 12
- 4.2 Fit viewer . . . 17

III Tutorial 23

5 Introductory series 25
- 5.1 Tutorials . . . 26

6 Simulating SMLM data 57
- 6.1 Requirement . . . 57
- 6.2 Task . . . 57
- 6.3 Main tutorial . . . 57

IV FAQ 63

7 How-to 65
- 7.1 SMAP basics . . . 65
- 7.2 LocMoFit GUI . . . 72

V Documentation 75

8 References 77
8.1 Model library . . . 77
8.2 Fitter . . . 87
8.3 SMLM models . . . 93
8.4 Geometric model classes . . . 96
8.5 Indices and tables . . . 98

MATLAB Module Index 99

Index 101

Part I

Introduction

CHAPTER

ONE

LOCMOFIT

1.1 Overview

LocMoFit (Localization Model Fit) is a model fitting software for SMLM data. It fits an arbitrary geometric model to the localization coordinates of a segmented structure. LocMoFit is described in the bioRxiv preprint Wu, Y.-L. et al. Maximum-likelihood model fitting for quantitative analysis of SMLM data. 2021.08.30.456756. *bioRxiv* (2021) doi:10.1101/2021.08.30.456756.

1.2 New to LocMoFit?

- First check *Getting started* in the menu on the left.

> **Important:** For a better user experience, we recommend you to install LocMoFit as a part of SMAP, a modular super-resolution microscopy analysis platform for SMLM data we developed.

- To know the structure and components of LocMoFit, see *Structure*.
- To learn how to use LocMoFit, start with the tutorial *Quick start*.

1.3 Content navigation

Information is structured as pages under different topics on the left:

- **BASICS** contains the necessary information for working with LocMoFit.
- **TUTORIAL** gets you familiar with LocMoFit through hands-on tutorials.
- **FAQ** provides answers to frequently asked questions.

You can find out details of functions and classes provided by LocMoFit in *References*.

CHAPTER

TWO

GETTING STARTED

LocMoFit is a tool developed in MATLAB. LocMoFit composites a set of classes and functions. You can download them here or get them as a part of SMAP (Super-resolution Microscopy Analysis Platform), a modular analysis platform for SMLM data. LocMoFit comes with its graphic user interface only in SMAP.

LocMoFit can be used in the following ways:

- **In MATLAB environments**, as classes and functions that can be called.
- As **a plugin of SMAP** (check section *Installation* for SMAP on GitHub), which also has a fully functional stand-alone version.

2.1 Requirements

Requirements differ according to the two following scenarios:

2.1.1 Running in MATLAB environments (with/without SMAP)

1. MATLAB 2022a (optional) and newer. Toolboxes: Optimization, Image processing, Curve fitting, Statistics, Machine Learning, and Global Optimization.
2. Mac or Windows.
3. SMAP (optional but highly recommended).

2.1.2 Running in the stand-alone version of SMAP

1. Mac or Windows.
2. The stand-alone version of SMAP (can be downloaded from here)

Note: The stand-alone version requires no MATLAB license but is limited in extendibility. Installation notes can be downloaded here.

3. MATLAB Runtime R2022a (no MATLAB license required).

2.2 Installation

2.2.1 With SMAP

You can access LocMoFit by installing SMAP. Check section *Installation* for SMAP on GitHub.

2.2.2 Without SMAP

You can download the zipped files of LocMoFit here. To install the code, simply unzip (usually in less than 5 min) the file and add the path of the unzipped folder to MATLAB.

2.3 Using LocMoFit with GUI now (SMAP required)

After the installation, we are ready to go. To learn more about the LocMoFit GUI, we recommend you to follow the tutorial *Quick start*.

Part II

Basics

CHAPTER

THREE

STRUCTURE OF LOCMOFIT

Important: If you would just like to try out LocMoFit, the information provided here is not essential. You can directly start with the tutorial *Quick start* and consult this page later.

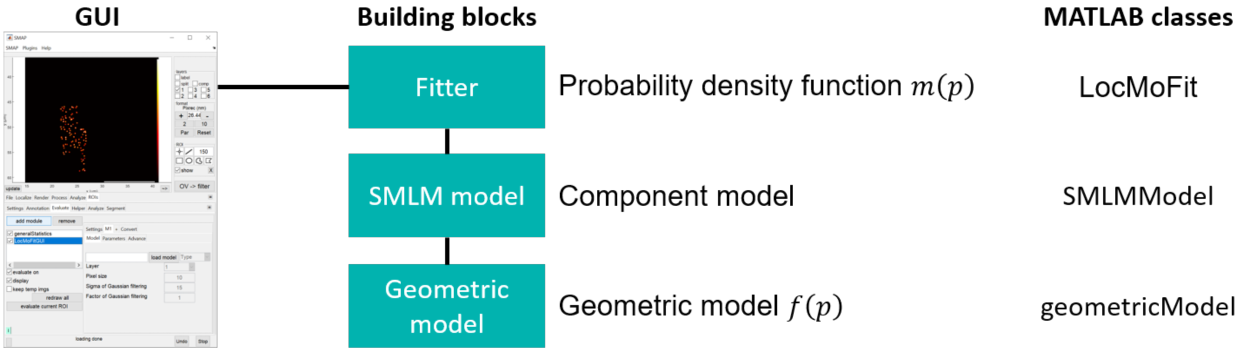

The individual geometry to be fitted to the data is defined in the **geometric model** (see `@geometricModel.geometricModel`). Model-specific parameters should be defined here. The geometric model is then handled by the **SMLM model** (see `@SMLMModel.SMLMModel`), where general parameters (positions, rotations, background … etc.) are incorporated. Finally, the SMLM model is converted to a probability density function (PDF) by the **fitter** (i.e., `@LocMoFit.LocMoFit`) and fitted to the data. Combining multiple SMLM models in the fitter to form a composite model is possible. The optimizer of choice and its settings should be defined in the fitter. The fitter then fits the PDF to the data.

Note: Most of the time, the user only has to interact with the fitter, either through the SMAP GUI or by calling the MATLAB classes.

3.1 Geometric model

We provide a list of *pre-defined geometric models*, including the ones used in the manuscript, which are ready to use. If none of them suit your purpose, you can build your own models, which require basic programming skills in MATLAB.

3.1.1 Model type and format

A geometric model can be defined as an image or a sub-class of `@geometricModel.geometricModel`.

- **Image**: *png* and *mat* files. A rigid template with only extrinsic parameters.
- **sub-class of geometricModel**: *m* files.

3.2 SMLM model

This class (and its sub-class) links a geometric model to the fitter and defines how the PDF is calculated. A geometric model has to be 'loaded' to an SMLM model to be controlled. Different model types are handled by different sub-classes:

- **Image**: handled by `@imageModel.imageModel`. The image serves as a density map. The likelihood value of each localization is calculated based on spatial interpolation on the map. Since the map is the same for all localizations, they share the same uncertainty, which is determined by how 'blurred' the image is.
- **sub-class of geometricModel**: handled by `@functionModel.functionModel`. There are two different ways of calculating the PDF:
  - **Continuous**: a model with both intrinsic and extrinsic parameters. During the optimization, a continuous model generates a density map and the likelihood value of each localization is calculated based on spatial interpolation on the map. Since the map is the same for all localizations, they share the same uncertainty (usually it is the mean value).
  - **Discrete**: a model with both intrinsic and extrinsic parameters. During the optimization, a discrete model generates a set of model points and the likelihood value of each localization is calculated based on its distance to each model point. The real uncertainty of each localization is used.

3.3 Fitter

The fitter (implemented as the class `@LocMoFit.LocMoFit`) controls and combines (if multiple SMLM models are loaded) geometries into a single PDF which is then fitted to localization data. The fitting is done by *maximum likelihood estimation*, i.e., varying the free structural parameters in order to maximize the log-likelihood, the matrice indicating a good fit. This procedure, termed optimization, is achieved by a specific optimizer (see below). After the fit, parameter estimates are then reported and the fit results are visualized.

Initial values of the structural parameters and their boundaries should be defined here. The optimizer of choice and its settings should also be defined here.

3.3.1 Optimizers

Currently, LocMoFit supports three different optimizers:

- **fmincon**: a nonlinear optimizer provided by MathWorks. This optimizer is a gradient-based method.
- **fminsearchbnd**: a derivative-free optimizer implemented by John D'Errico, based on the fminsearch optimizer by MathWorks.
- **particleswarm**: an optimizer that searches the parameters more globally. Also provided by MathWorks.

CHAPTER

FOUR

GRAPHIC USER INTERFACE (GUI)

4.1 GUI overview

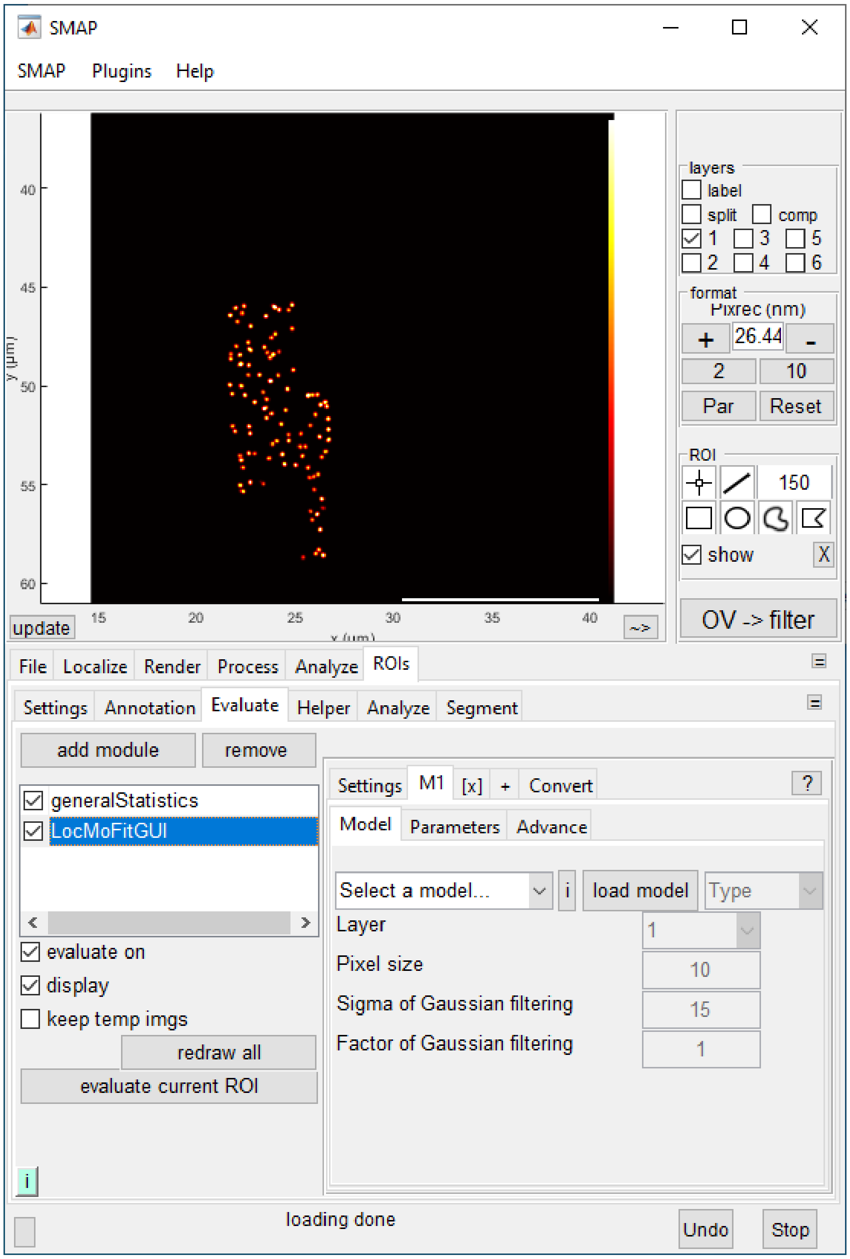

LocMoFit is integrated into SMAP. You can follow the steps to access to LocMoFit in SMAP:

1. Go to the **[ROIs]** tab.
2. Go to **[Evaluate]** tab and click **add module**.
3. In the popup window, select *LocMoFitGUI* and click *ok*.

Now the SMAP window should look like the image above. There are three main tabs in the LocMoFit GUI: **[Settings]**,**[M1]**, and **[Convert]** and a button **[+]**.

Note: The button **[+]** here allows you to add one more model.

Here I will introduce them in the order of a routine workflow.

4.1.1 Tab *[M1]*: Model 1

This is the first tab you will see once the module *LocMoFitGUI* is added. In this tab, you can load and set up the first SMLM model.

Sub-tab *[Model]*

- a button **load model** allows you to load a SMLM model.
- a pop-up menu *Type* allows you to specify the model type. You can find out more about the model types here.
- a pop-up menu *Layer* allows you to define which layer this model will be fitted to.
- a field *Pixel size* for you to define the pixel size. This only applies to an *image* or *continuous* model.
- a field *Sigma of Gaussian filtering* where you can specify a constant sigma of Gaussian filtering. The higher the more blurred.
- a field *Factor of Gaussian filtering* where you can specify the factor of Gaussian filtering. The higher the more blurred.

Note: *Sigma of Gaussian filtering* and *Factor of Gaussian filtering* exclude each other.

Sub-tab *[Parameters]*

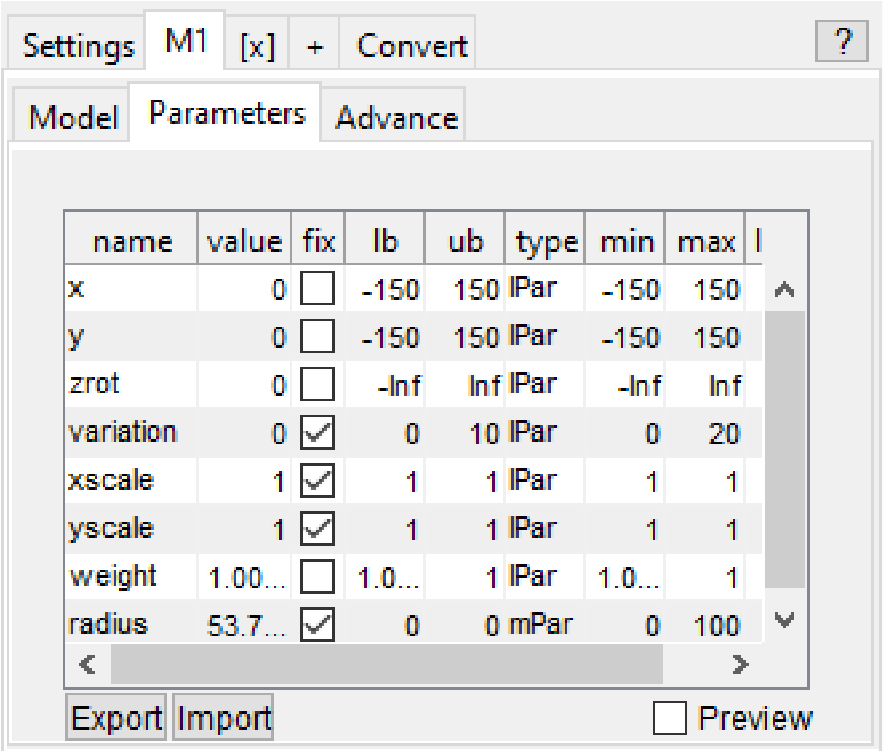

If you already loaded a model, you can move on to this tab and set up the model parameters. Here you will see the following:

- the main table where you can set up the model parameters. See parameter table for more details.
- buttons **Save** and **Load** allow you to save and load settings.
- a button **Pick site** allows you to click a site in the ROI manager without evaluating the fit. This is usually used together with the button *Preview*
- a button **Preview** to show the model with the initial parameters.

Description of the fields

- *name*: parameter names.
- *value*: initial values of parameters.
- *fix*: specify the parameter should be fix or or not. If checked, the parameter will be set to the value you defined and will be a constant that are not fitted.
- *lb*: relative lower boundaries of parameter ranges.
- *ub*: relative upper boundaries of parameter ranges.
- *type*: types of parameters. This is not editable.
- *min*: absolute lower boundaries of parameter ranges.
- *max*: absolute upper boundaries of parameter ranges.

- *label*: user-defined labels for the corresponding parameters.

Note: For example, *value* 30, *lb* -10, *ub* 20, *min* 10, and *max* 45 results to a parameter range of [20 45]. This is based on first get [value+lb value+ub] = [20 50], and then check whether this is beyond [min max] = [10 45] or not. If this is the case, the range will be set to the min or max values so the final upper boundary is 45 but not 50.

Sub-tab *[Advance]*

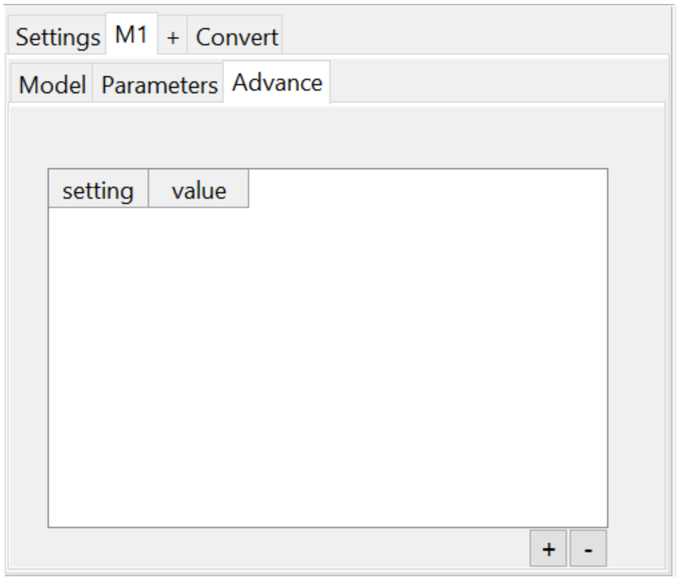

You can set up the advanced settings here. The setting items are model-specific. Here you will see the following:

- the main table where you can set up the advanced settings. You can choose a setting item that you want to modify in the column *Setting* and define its value in the column *Value*.
- buttons **+** and **-** allows you to add/remove a setting item.

Note: You can click the button **[+]** next to the tab label **[M1]** to add one more model as **[M2]**.

4.1.2 Tab *[Settings]*

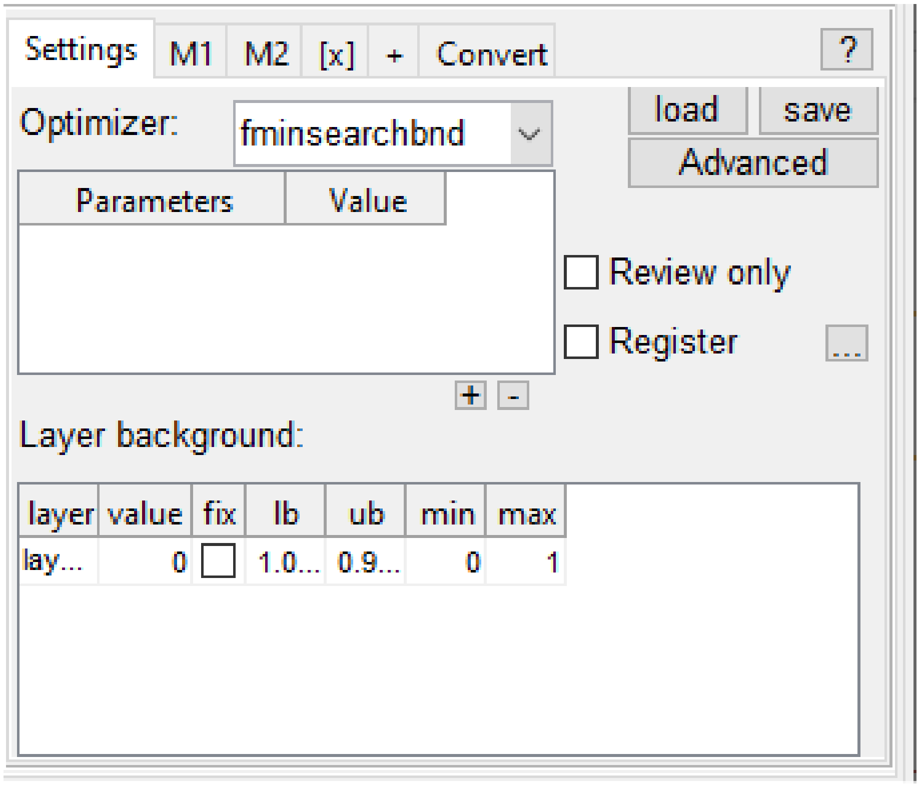

In this tab, you can set up settings beyond specific models and have a global control of the current LocMoFitGUI instance. Here you will see the following:

- a pop-up menu *Optimizer* allows you to define which optimizer to use.
- a table of *optimizer parameters* and buttons **+** and **-**. Here you can define parameters of the optimizer. Use **+/-** to add or remove a parameter.
- buttons **load** and **save** allow you to load and save the entire settings.
- a button **advanced** for advanced settings. Usually you don't have to touch this part. If you click it, you will get a list of advanced settings in a new pop-up window.
- a checkbox *Review only*. Check it if you have fitted the current site and just want to review the fit.
- a checkbox *Transform*. Check it if you would like to transform the site based on the model with the final parameter estimates.
- a table of *Layer background*. This is similar to a general parameter table.

4.1.3 Tab *[Convert]*

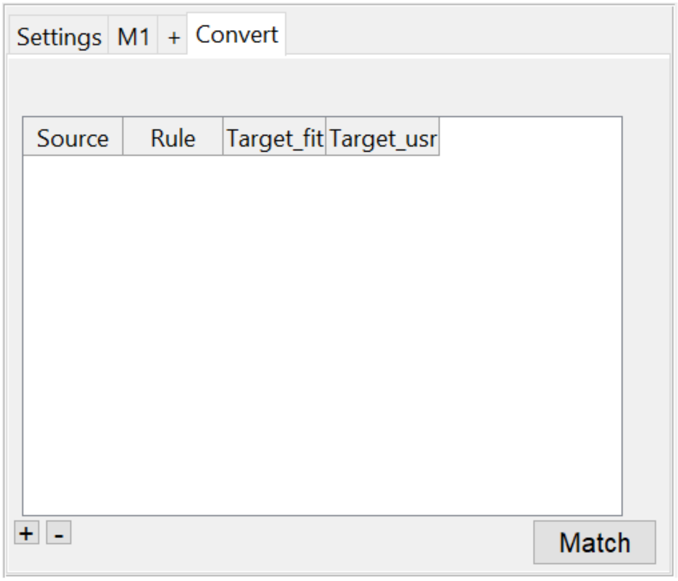

Instead of constants, you can define initial parameters with user-defined rules here. Here you will see the following:

- the main table where you can set up the rules for converting them to parameter values. The meanings of the columns:
  - *Source*: source LocMoFitGUI instance/step.
  - *Rule*: rules for defining the target.
  - *Target_fit*: target parameters in the fit.
  - *Target_usr*: target temporal user-defined variables.
- buttons **+** and **-** allow you to add or remove a rule.
- a button **Match** for matching the parameters with the same names from a previous step. These define the value of matched ones based on the final estimates of the previous step.

Note: If you defined a conversion rule here for a parameter, the initial value of the parameter defined in the sub-tab [Mxx]/[Parameters] will be ignored.

4.2 Fit viewer

After the current site is fitted, the fit viewer will show if the **display** is checked. This viewer is different per model type and data dimension.

4.2.1 For 3D continuous/discrete models

You should see this window show up:

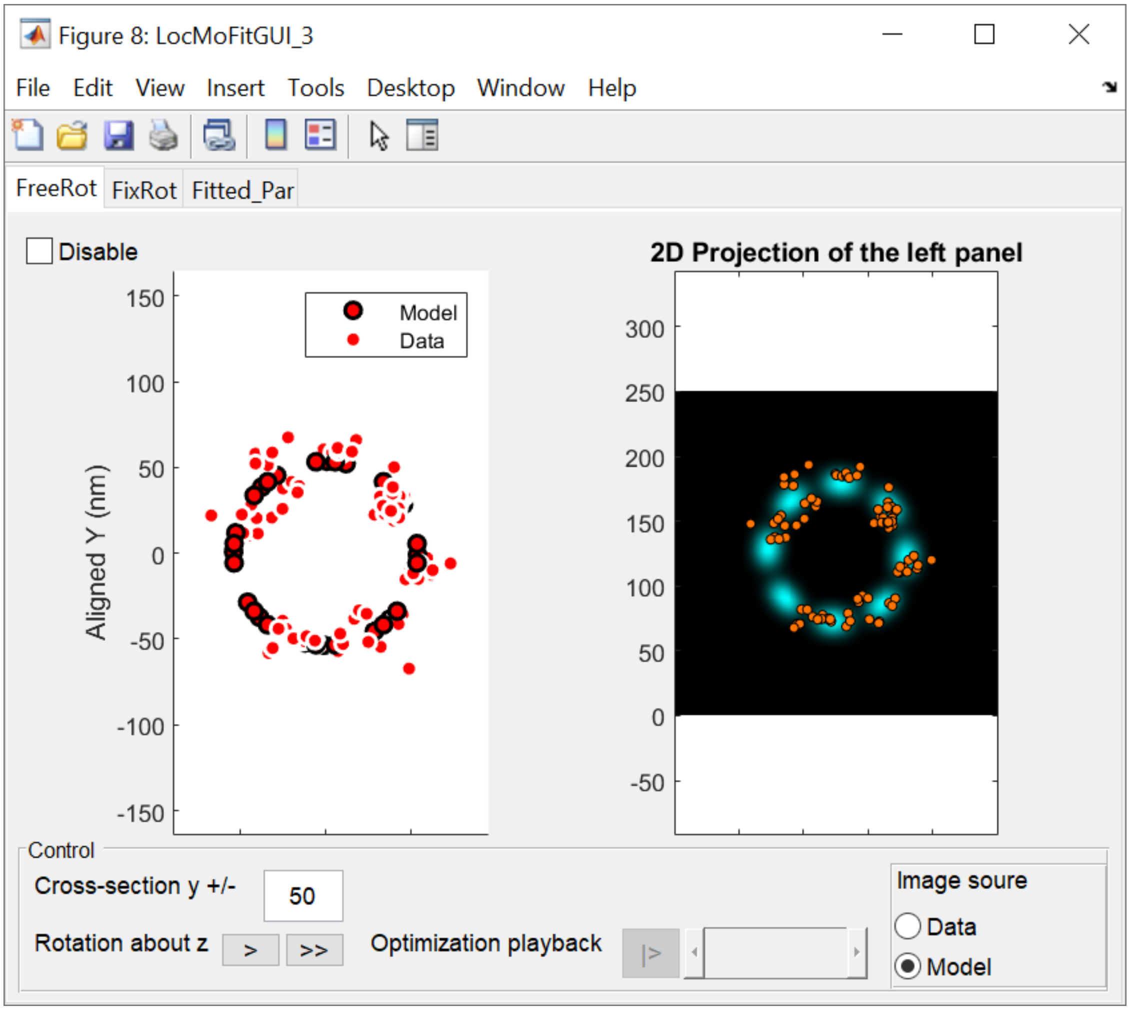

Tab *[FreeRot]*

Here you will see the following:

- a checkbox **Disable**. For efficiency, you can check it so that the plots will not be drawn.
- the left panel *Dot plot*. Here both the model and the data are represented by dots.
- the right panel *2D Projection of the left panel*. Here either the model or the data is rendered as a image. You can specify the image source bellow in the panel *Control*.
- the bottom panel *Control*. Here you can:
  - define the thickness of the cross-section along the y-axis.
  - rotate the site about z-axis with the buttons > and >> in small and large steps, respectively.
  - You can playback the process of optimization if its history is saved.

Note: You can enable the history saving by turning on the *OutputFun* in the table *optimizer parameters*

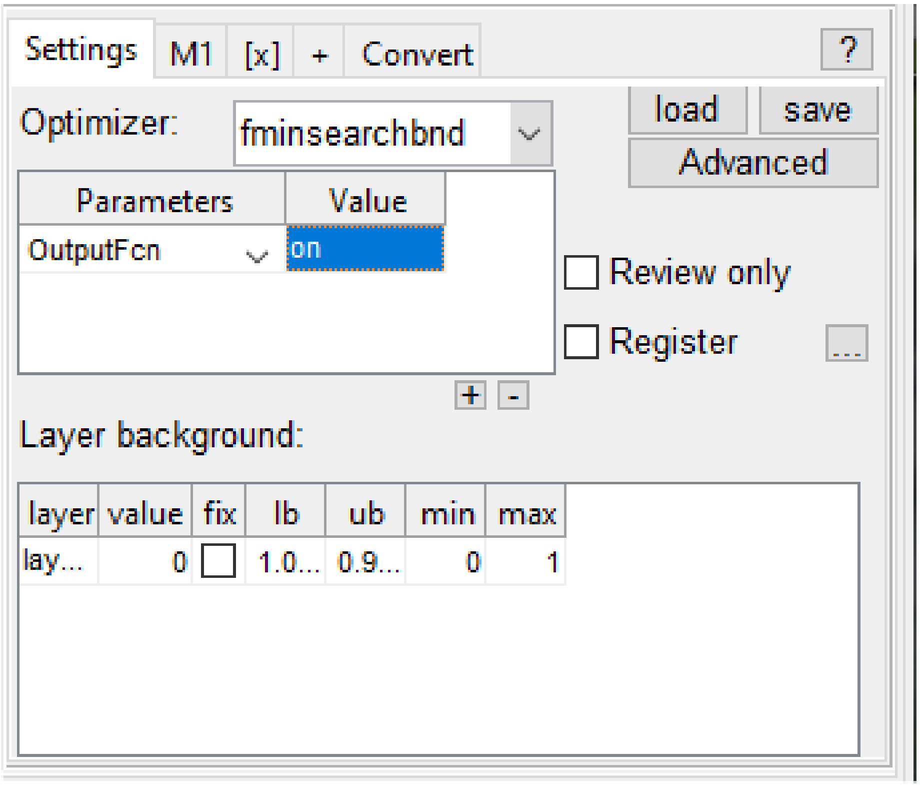

Tab *[FixRot]*

Note: By default, this tab is disabled. You can uncheck the checkbox **Disable** to show the content.

- a checkbox **Disable**. For efficiency, you can check it so that the plots will not be drawn.
- four panels of the fitted site with different rotation angles around the z-axis.

Tab *[Fitted_Par]*

Note: For each viewer, it comes with a tab **[Fitted_Par]** regardless of the model type:

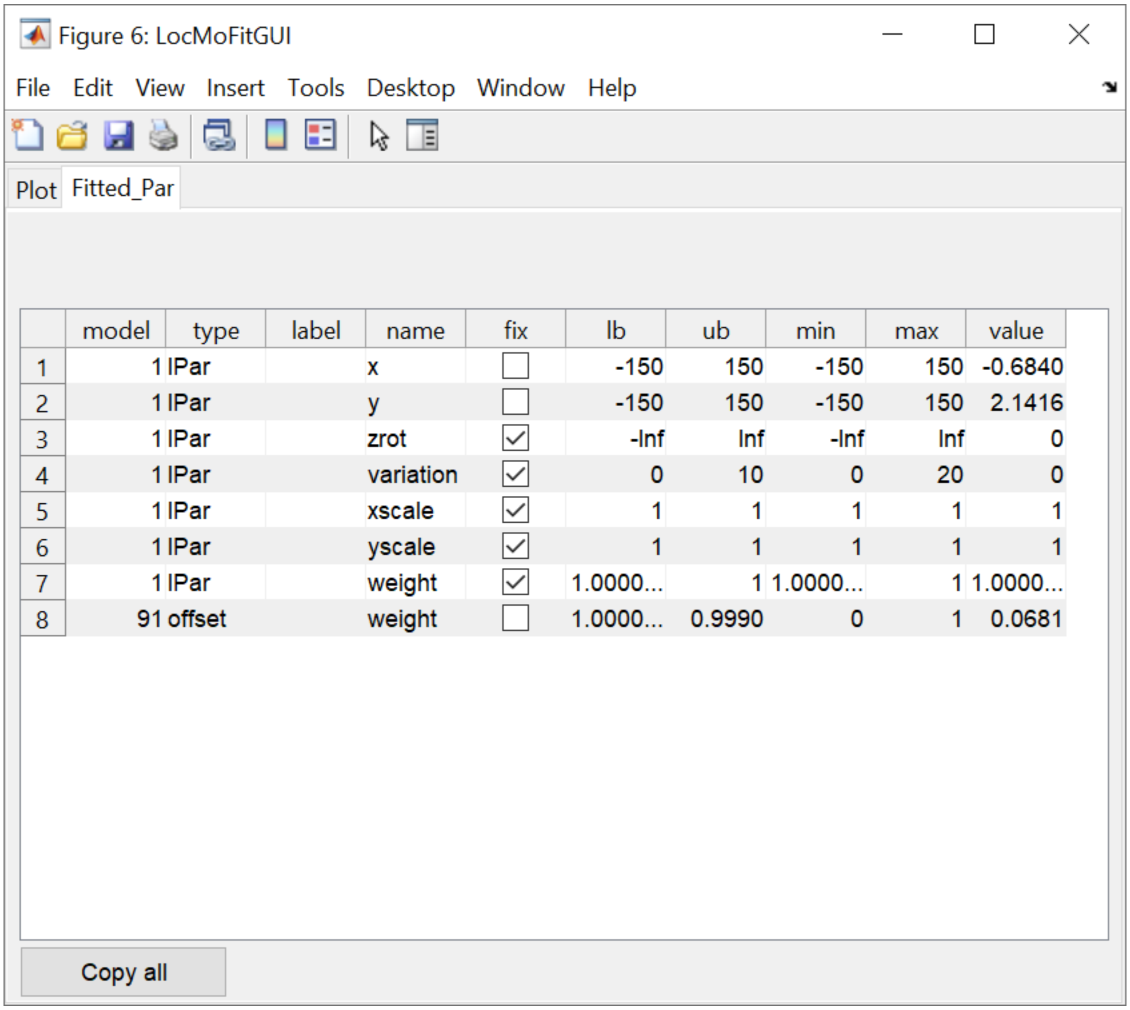

	model	type	label	name	fix	lb	ub	min	max	value
1	1	lPar		x	☐	-150	150	-150	150	-0.6840
2	1	lPar		y	☐	-150	150	-150	150	2.1416
3	1	lPar		zrot	☑	-Inf	Inf	-Inf	Inf	0
4	1	lPar		variation	☑	0	10	0	20	0
5	1	lPar		xscale	☑	1	1	1	1	1
6	1	lPar		yscale	☑	1	1	1	1	1
7	1	lPar		weight	☑	1.0000...	1	1.0000...	1	1.0000...
8	91	offset		weight	☐	1.0000...	0.9990	0	1	0.0681

- the main table that displays the parameter settings with parameter estimates shown in the column *Value*.
- a button **Copy all** allows you to copy the entire table to the clipboard.

4.2.2 For 3D image models

You should see this window show up:

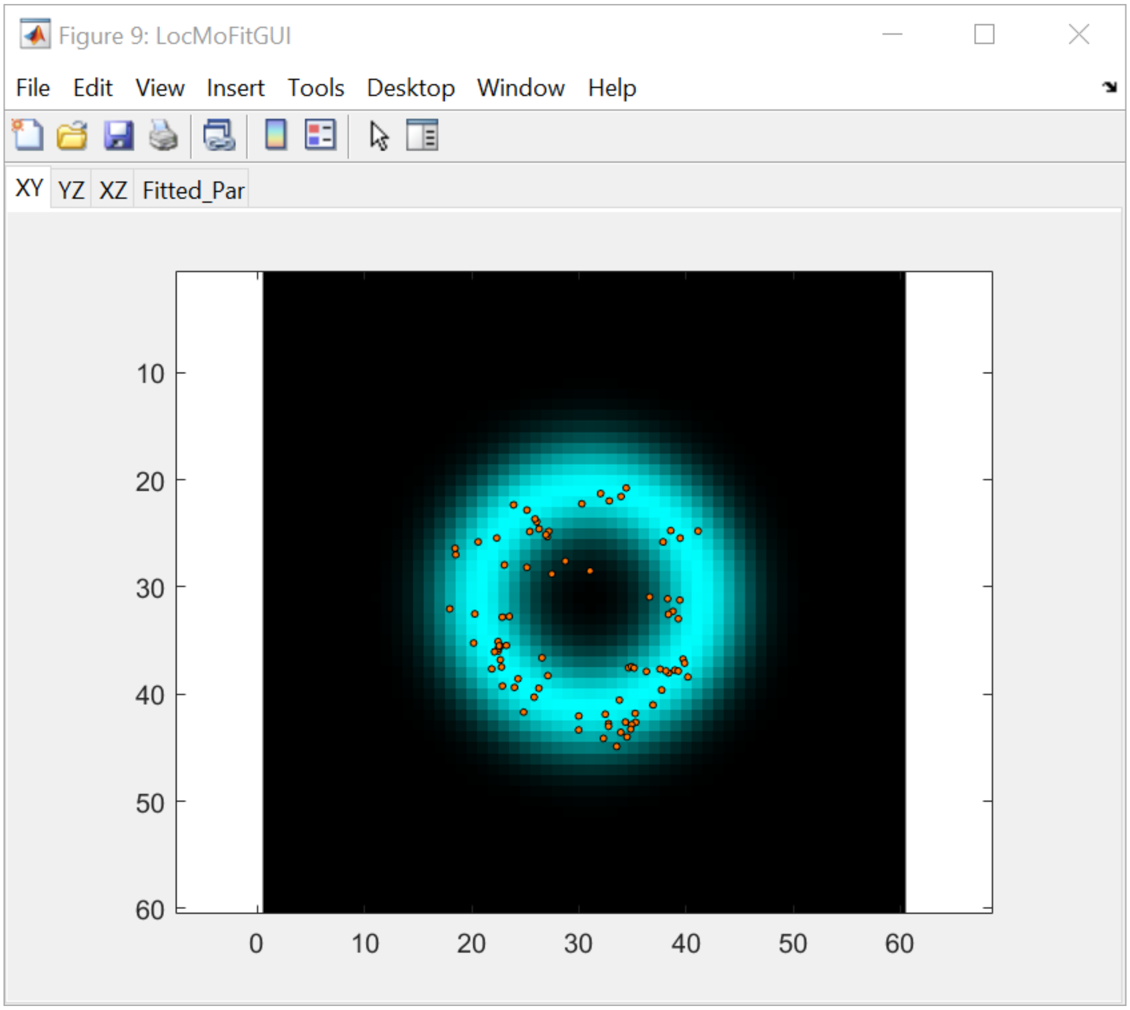

There are three projection tabs showing the site in different views (planes **[XY]**, **[YZ]**, and **[XZ]**) and the common tab **[Fitted_Par]**.

4.2.3 For 2D models

You should see this window show up:

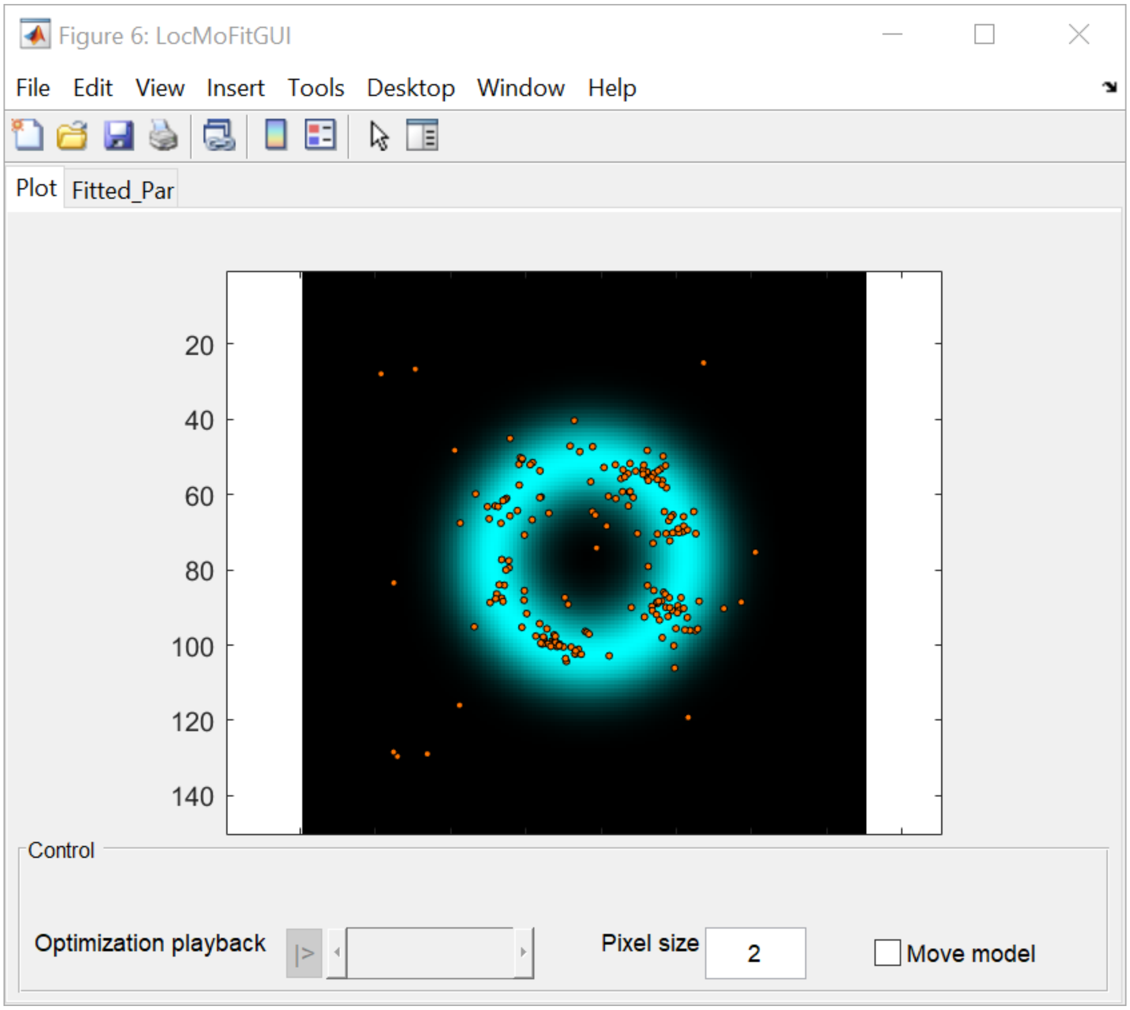

There are two tabs:

- **[Plot]** showing the data as points and the model as an image. In the control panel:
  - you can playback the process of optimization if its history is saved.
  - you can define the pxiel size for rendered image above.
  - normally the data is transforemd. You can transform the model instead by checking the checkbox **Move model**.
- the common tab **[Fitted_Par]**.

Part III

Tutorial

CHAPTER

FIVE

INTRODUCTORY SERIES

This series is designed for a beginner user to get an overview of LocMoFit GUI. You will learn how to define the setting steps in order to extract structural parameters by fitting geometric models.

Here, the nuclear pore complex (NPC) will be used as the example structure. After the tutorials, you should be able to reproduce the workflow we applied to extract structural parameters of individual NPCs (see Fig. 2a-f in our manuscript describing LocMoFit).

The individual NPCs appear as rings if you see them in their top views. In its side view, an NPC appears as two parallel rings. In the example dataset we will be using, we genetically labeled Nup96, a protein that has 32 copies per NPC. Two copies form a unit that appears eight times in one ring. Therefore, the rings look discontinuous. Also, because the labeling efficiency is not 100% (some of the protein copies are not visible in the images), some rings look incomplete.

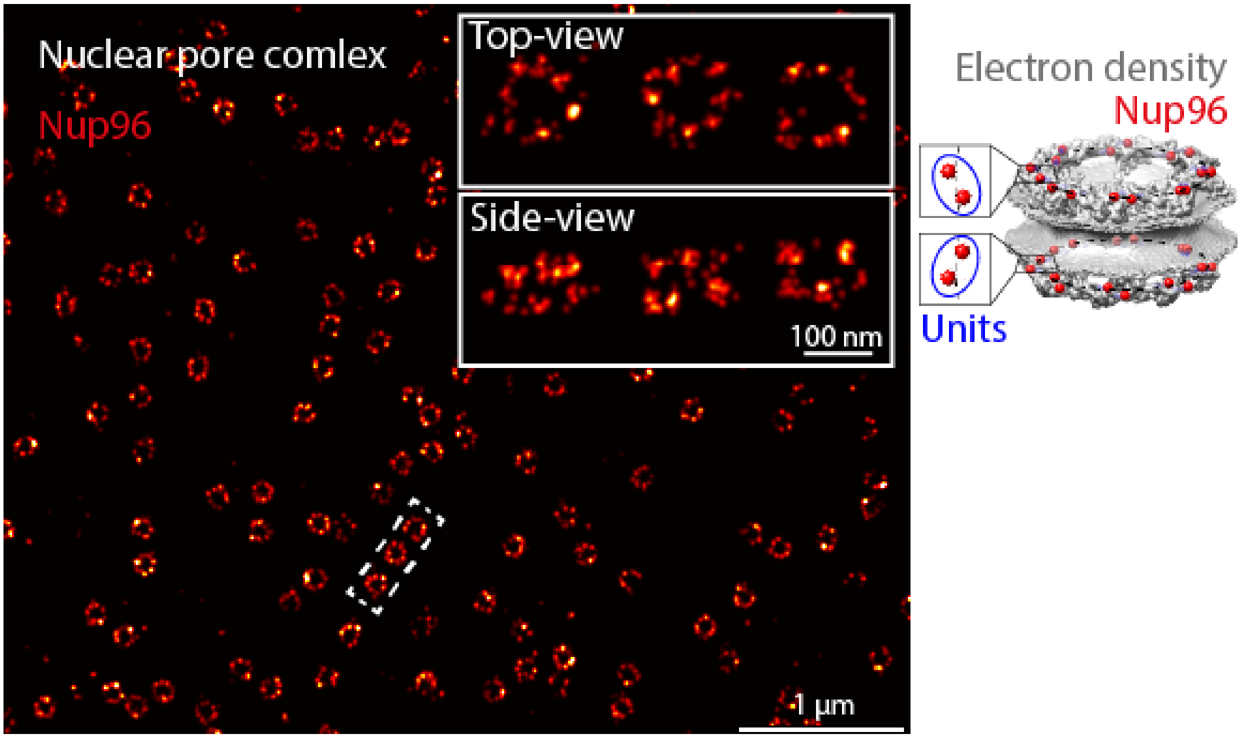

We are particularly interested in the radius, ring separation, and ring twist, the three key structural parameters of the NPC. In this series, we will show you how to extract these parameters using LocMoFit step-by-step.

5.1 Tutorials

- In **Quick start**, you will perform a simple fit: fitting top-view projections of the NPCs with a ring model. This helps you to get familiar with the LocMoFit GUI.
- In **Composite model**, you will extend the fit to 3D and extract the ring separation based on a dual-ring model you will build yourself.
- In **Chain steps**, you will learn how to extract the three parameters robustly by chaining different fitting steps: transitioning from a smooth dual-ring model to a detailed eight-fold rotationally symmetrical model.

Time required: 45 min - 1 hr for all tutorials.

5.1.1 Quick start

Note: Time required: ~15 min.

LocMoFit fits a geometry to single structures in SMLM data. In this tutorial, you will learn how to do this with the LocMoFit GUI in SMAP (what is SMAP?).

We will be using the nuclear pore complex (NPC) as an example. This complex appears as rings if you see them in the top view.

Task

Fitting a 2D ring model to the top-view projection of NPCs to find their positions.

Requirement

- Software: SMAP installed. Further information can be found on our GitHub site.
- Localization data: *U2OS_Nup96_BG-AF647_demo_sml.mat*

Main tutorial

Preparation

1. Start **SMAP** (*how to?*).
2. Load the localization data (*how to?*) *U2OS_Nup96_BG-AF647_demo_sml.mat*. This file contains segmented NPCs that you will be analyzing in the following steps.

Note: How was the segmentation done? You can check out all the pre-processing steps from *fitting raw data (raw camera frames)* to *segmentation of NPCs* in **SMAP_manual_NPC.pdf**.

The current window:

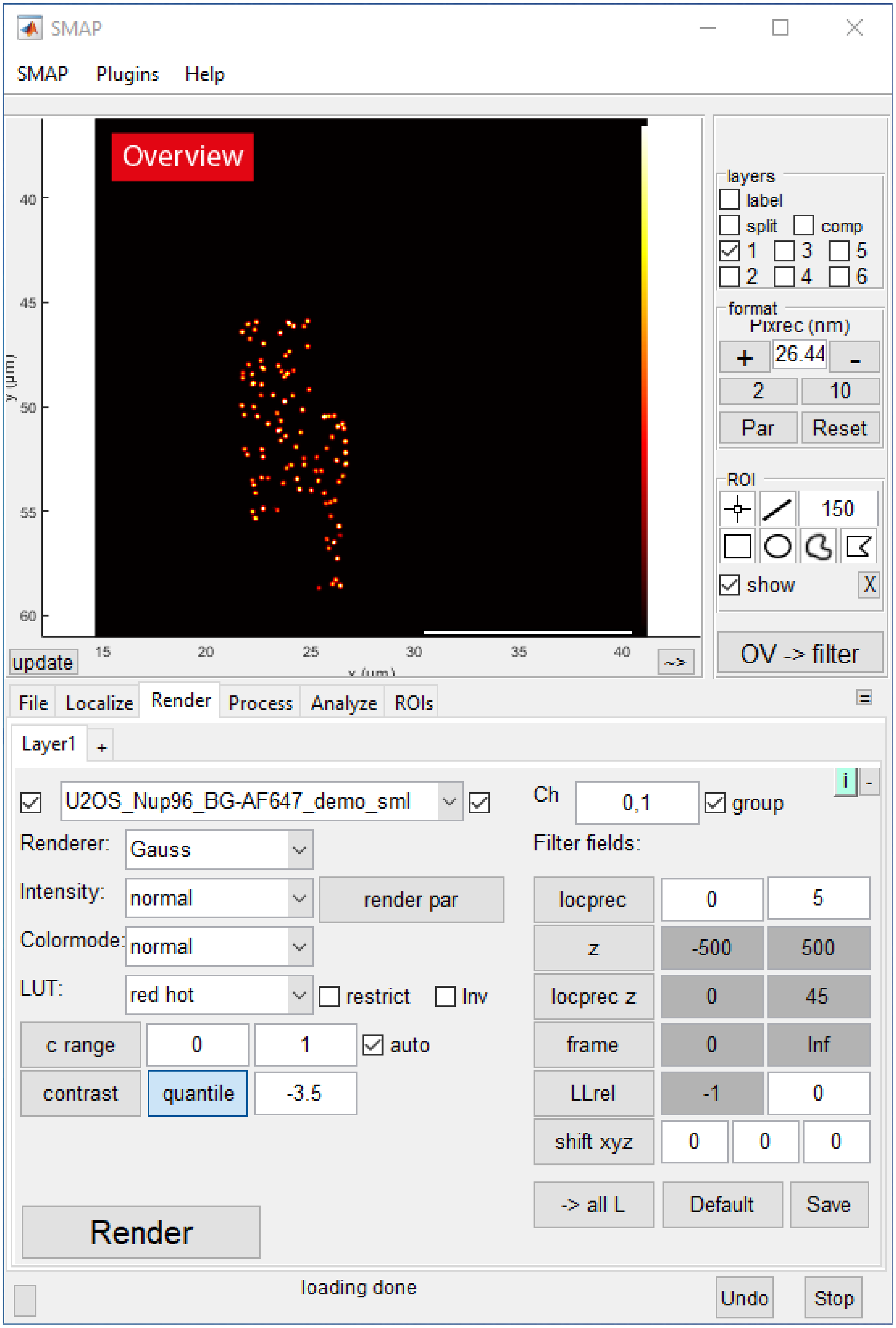
SMAP
SMAP Plugins Help
Overview
40
45
50
55
60
y (µm)
15 20 25 30 35 40
update
layers
label
split comp
1 3 5
2 4 6
format
Pixrec (nm)
+ 26.44 -
2 10
Par Reset
ROI
150
show X
OV -> filter
File Localize Render Process Analyze ROIs
Layer1 +
U2OS_Nup96_BG-AF647_demo_sml
Ch 0,1 group
Renderer: Gauss
Filter fields:
Intensity: normal
render par
Colormode: normal
LUT: red hot
restrict Inv
c range 0 1 auto
contrast quantile -3.5
locprec 0 5
z -500 500
locprec z 0 45
frame 0 Inf
LLrel -1 0
shift xyz 0 0 0
-> all L Default Save
Render
loading done
Undo Stop

You should see the data set displayed in the **Overview**.

Warm-up

Before fitting, let us explore the data a bit first. We can find the list of segmented NPCs in the **ROIManager**:

1. Go to **[ROIs]** -> **[Settings]**, click **show ROI manager**. This opens the **ROIManager** in a new window.

   Note: **ROIManager** allows you to manage ROIs in different cells and files. Check section 8.2 (*Manually generating a list of ROIs*) in **SMAP_manual_NPC.pdf** for more information.

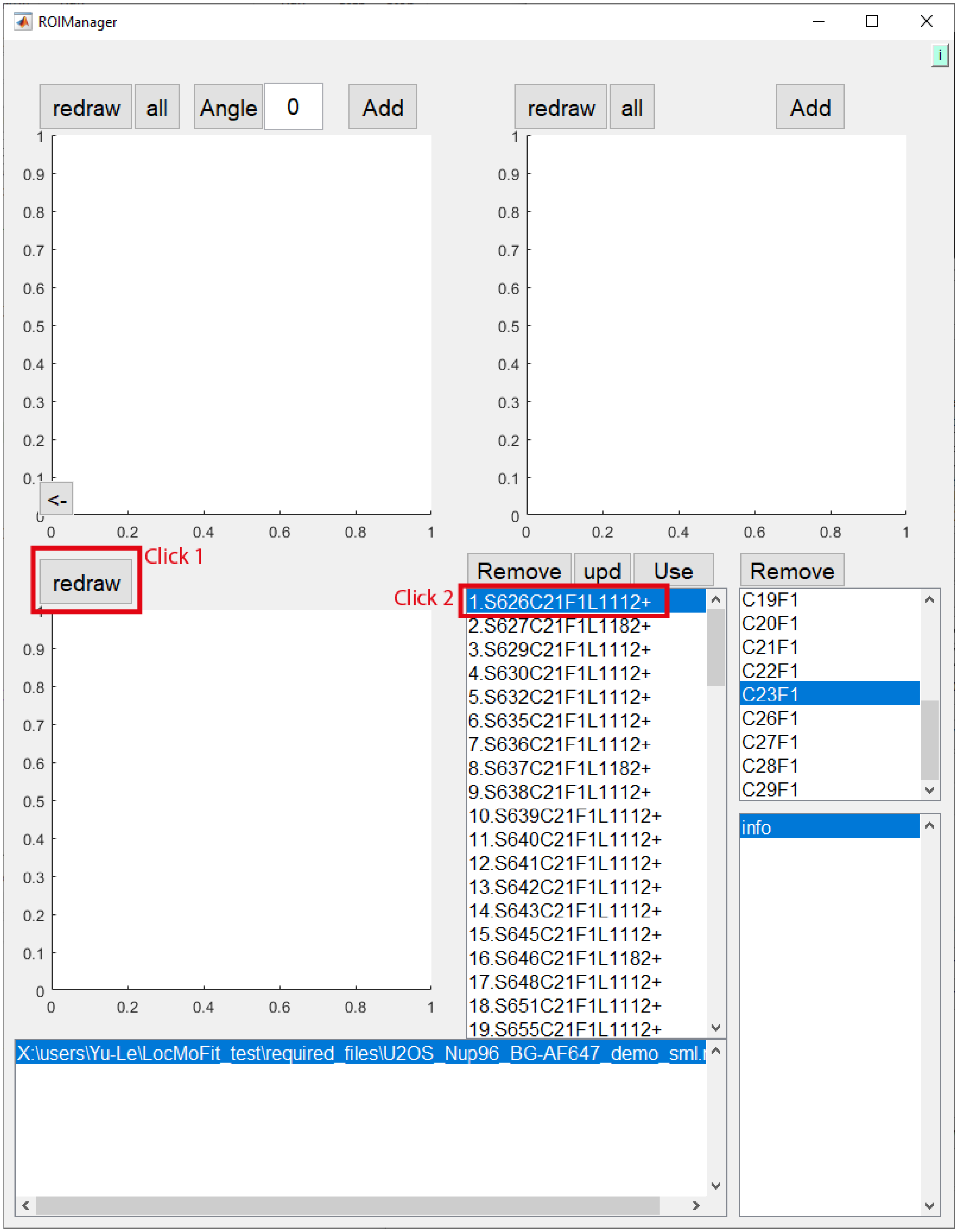

2. Now you see empty boxes. To show images properly, click the **redraw** button and **one site** as in the image above. The window should look like this now:

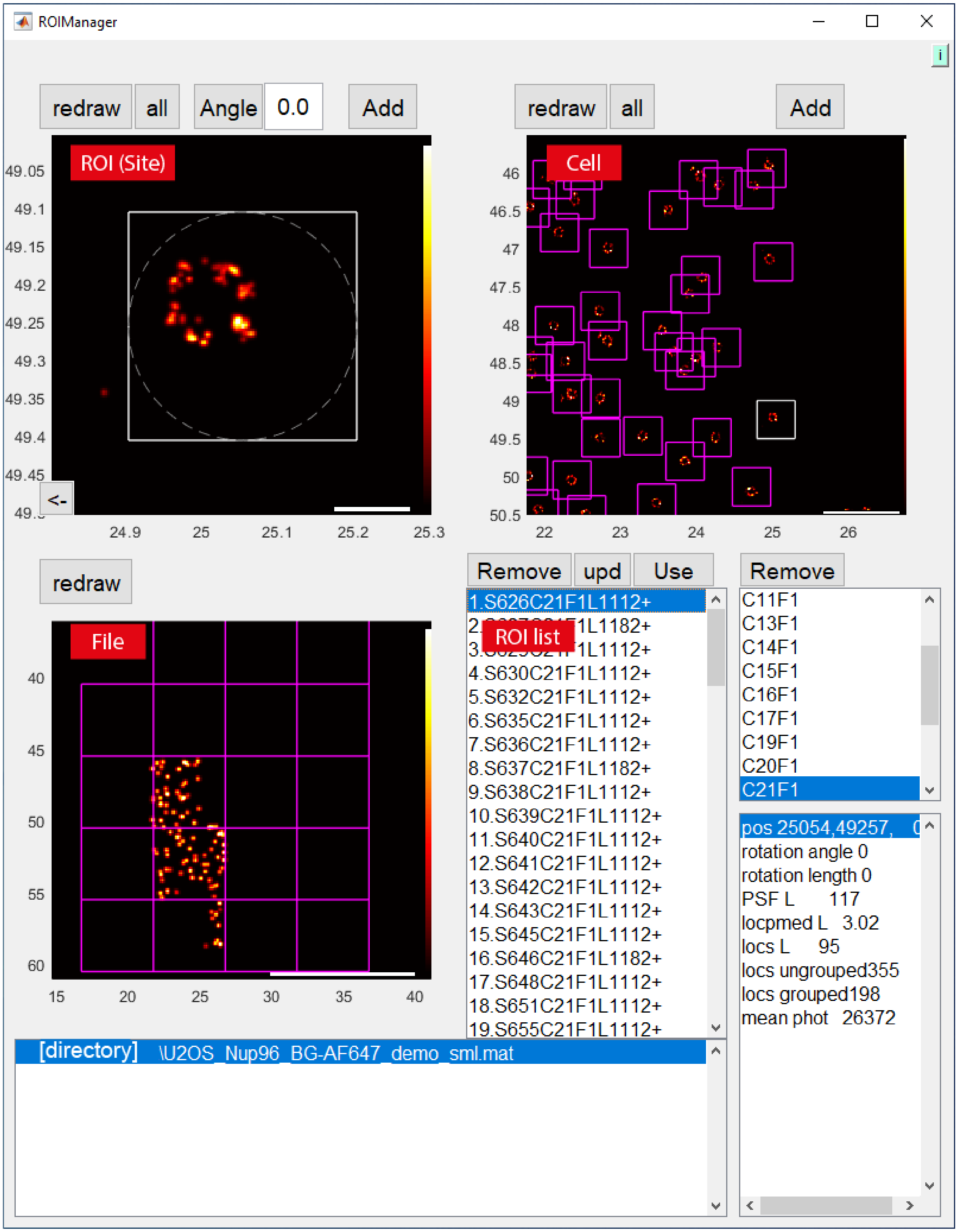

3. Click a few sites in the ROI list to display them.

Loading LocMoFit

Let us now start to work with LocMoFit by loading it into SMAP:

1. Go to the **[ROIs]** tab.
2. Go to **[Evaluate]** tab and click **add module**.

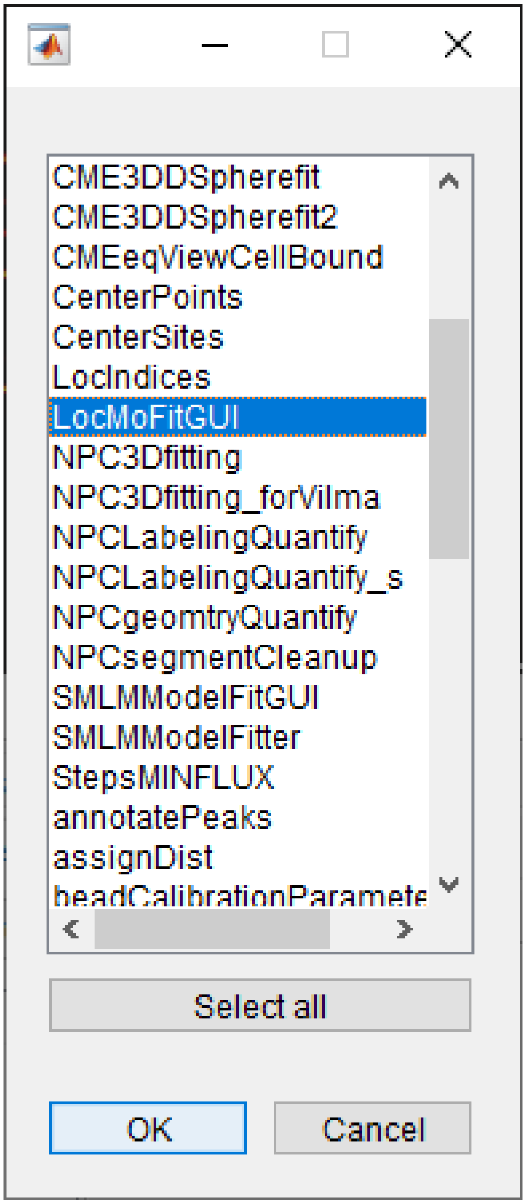

3. In the popup window, select *LocMoFitGUI* and click *ok*.
4. Show the *LocMoFitGUI* GUI by clicking on it in the list of loaded modules. Your SMAP window should look like this now:

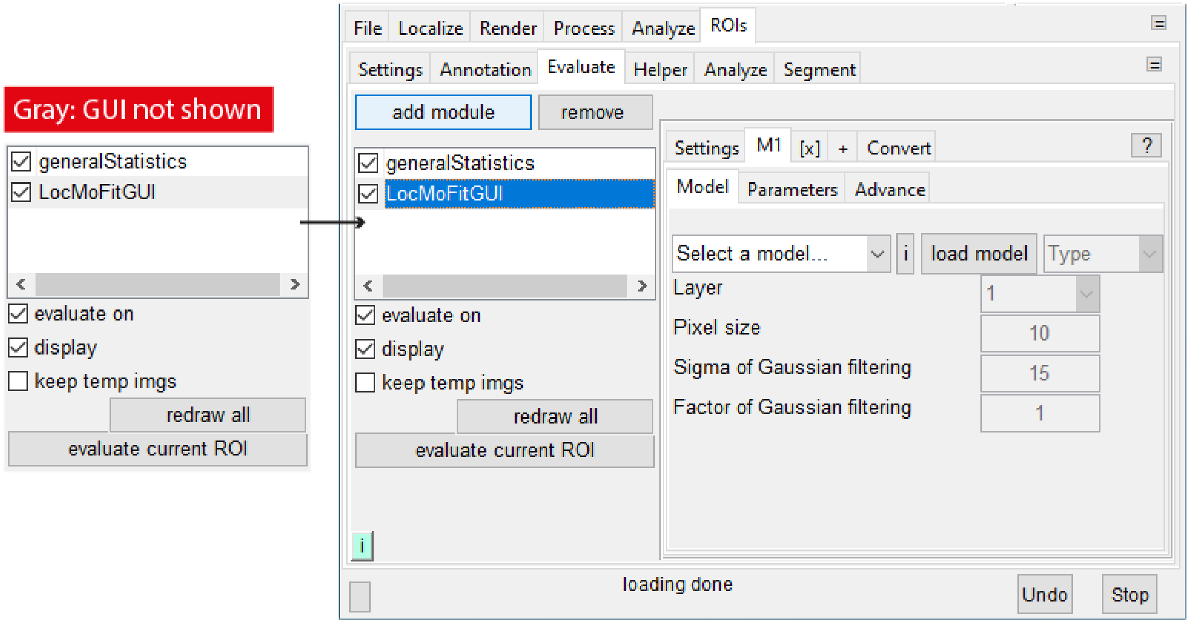

Note: The button 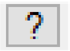 in the up-right corner of the LocMoFit GUI provides details of fields/buttons in the current tab/sub-tab.

Setup

We will be using the model *`ring2D`* here (more about model types).

1. First, we have to load the model into LocMoFit:

   - In the right panel, go to **[M1]** -> **[Model]**, click the drop-down menu (where *select the model…* is shown), and then select *ring2D*.

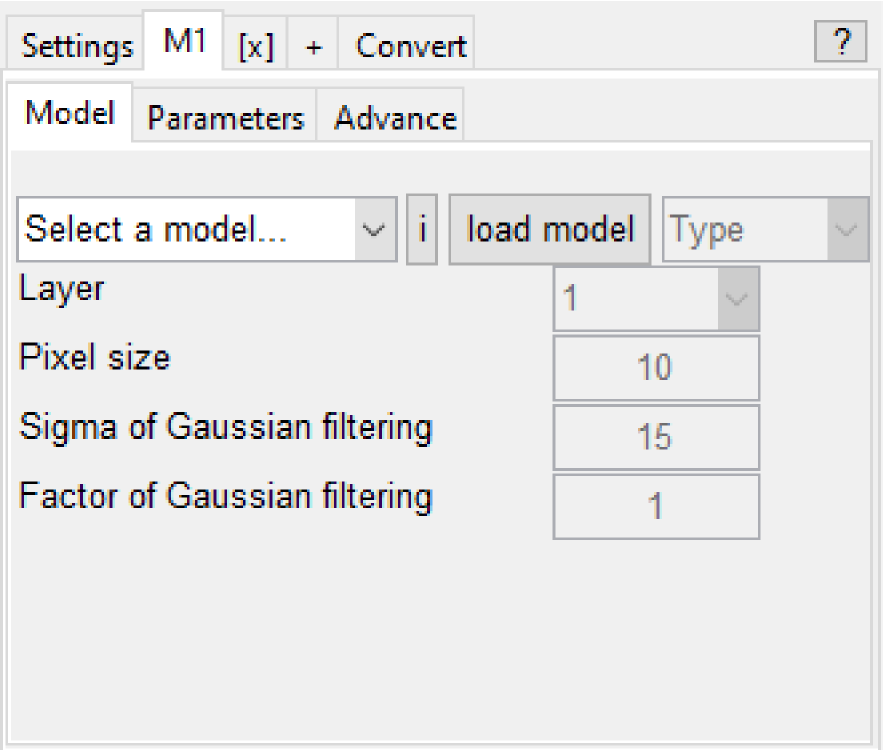

- Click **load model**. Now the model is loaded.

Note: **M1** stands for model 1. Find out more about the M1 tab here. LocMoFit allows you to load multiple models and combine them into a single composite model, which you will learn in the next tutorial.

Note: Clicking the button 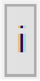 next to the drop-down menu opens the webpage detailing the selected model.

2. Next, we set up the parameter settings:

   - Go to the tab **[Parameters]**

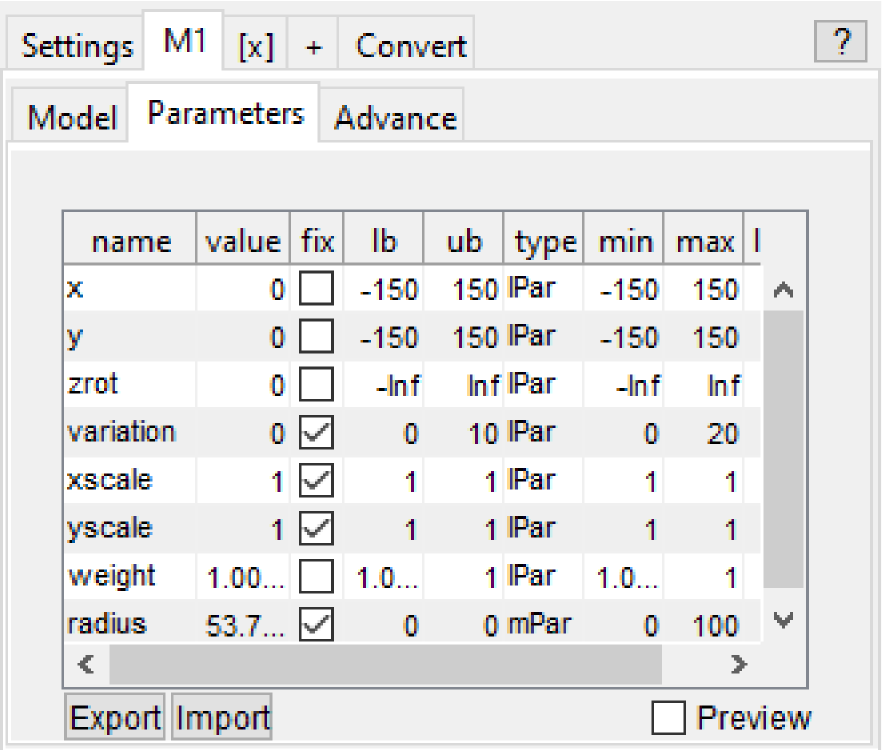

- Fill in the table as followed (non-default values are in bold):

name	value	fix	lb	ub	type	min	max
x	0	☐	-150	150	lPar	-150	150
y	0	☐	-150	150	lPar	-150	150
zrot	0	☑	-Inf	Inf	lPar	-Inf	Inf
variation	**10**	☑	0	10	lPar	0	20
xscale	1	☑	1	1	lPar	1	1
yscale	1	☑	1	1	lPar	1	1
weight	**1**	☑	1e-05	1	lPar	1e-05	1
radius	53.7	☑	0	0	mPar	0	100

Important: What do all these different elements mean? Let's start with the fields **name** and **type**. **name** shows parameter names. **type** indicates the types of parameters:

- **lPar**: extrinsic parameters, which are independent of the geometries. These parameters therefore apply to different geometries.
- **mPar**: intrinsic parameters, which determine the shape of the geometry and therefore are geometry-specific.

Parameters *x* and *y* are the xy coordinates of the model with respect to the center of the ROI. *zrot* is the rotation around the z-axis. xscale and yscale are the scaling of the model along the respective axes. *variation* is the extra variation, the uncertainties that cannot be solely explained by the localization precision. It basically defines how blurred the model is. The field **fix** indicates whether each parameter is fixed to a specific value or not. If checked, the corresponding parameter will not be estimated but fixed to the value defined in the field **value**.

The field **value** defines the initial values of the parameters. The fields **min** and **max** define the search range of a parameter. We will discuss other fields later.

With the settings, now you defined a ring with a radius = 53.7 nm. The ring can be moved in the xy plane freely from -150 to 150 nm.

Preview the model before fitting

In practice, you often have to optimize the fitting settings, especially finding good initial parameters. In this case, previewing the model with the initial parameters is useful.

1. To activate the preview mode, check the checkbox **preview** in the bottom-left corner of the tab **[Parameters]**.
2. Go back to **[Evaluate]**, make sure that **evaluate on** and **display** in the left panel are checked.

Note:

- The loaded modules will be evaluated only when **evaluate on** is checked.
- Result windows of the loaded modules will be displayed only when **display** is checked.

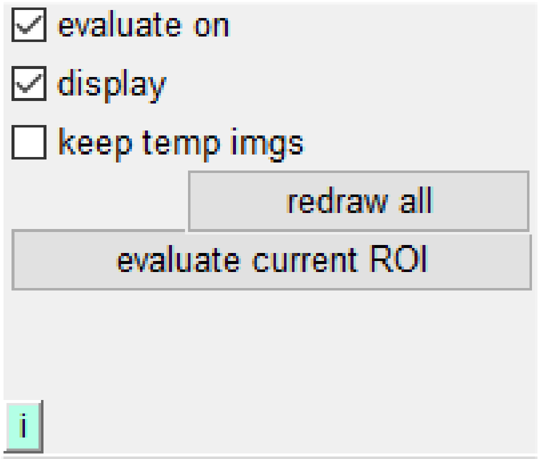

---

3. Now click on the first ROI in the *ROI manager* window and wait for a few seconds. You should see a new window *LocMoFitGUI*, in which the localizations are plotted on the initial model.

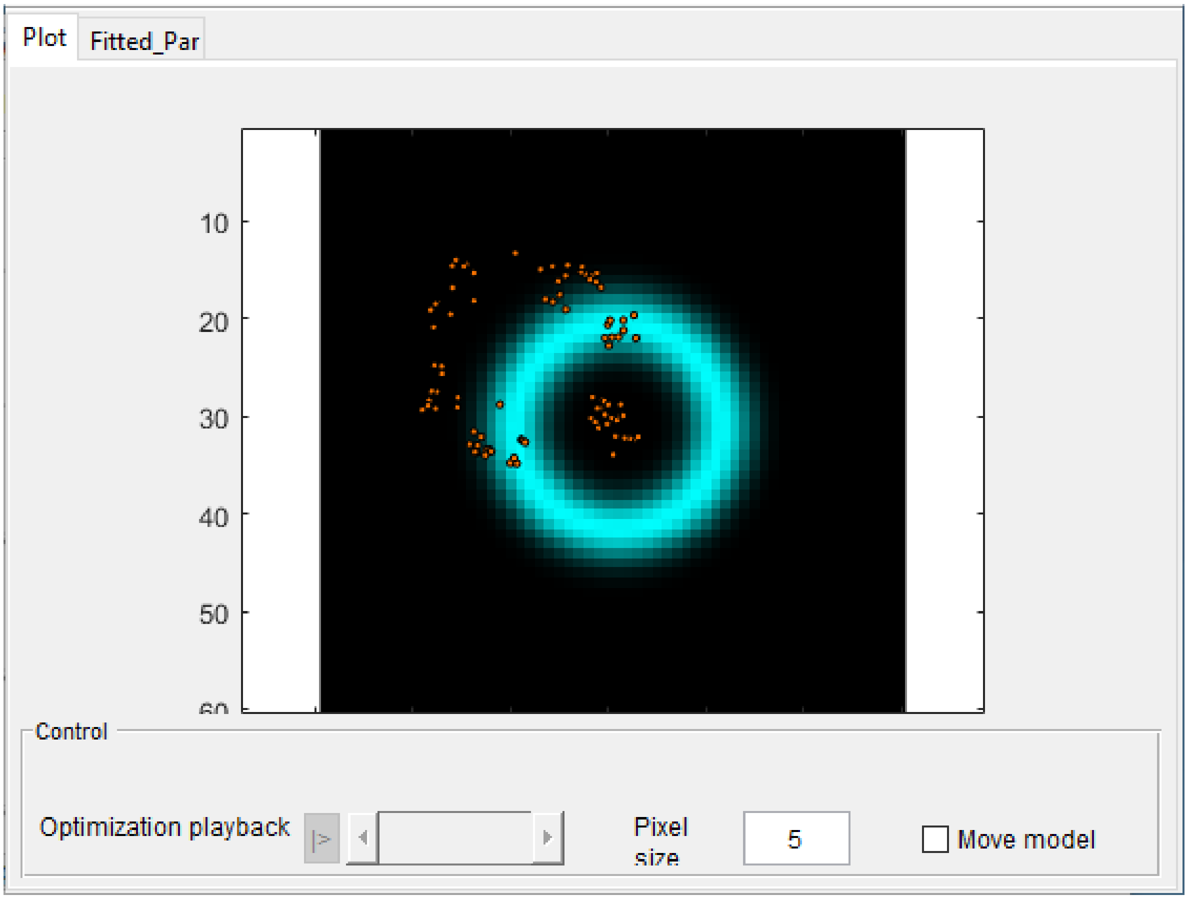

4. You can explore the data more by repeating these steps for a few more ROIs.

Fitting

Next, we will execute the fitting. This is done by clicking the site in the list of sites in the *ROI manager* window with the preview box unchecked:

1. Go back the tab **[Parameters]** and uncheck **preview**.
2. In the *ROI list* of *ROI Manager* window, click on site 1 and wait for a few seconds. You should see the updated *LocMoFitGUI* window displaying the fitted model.

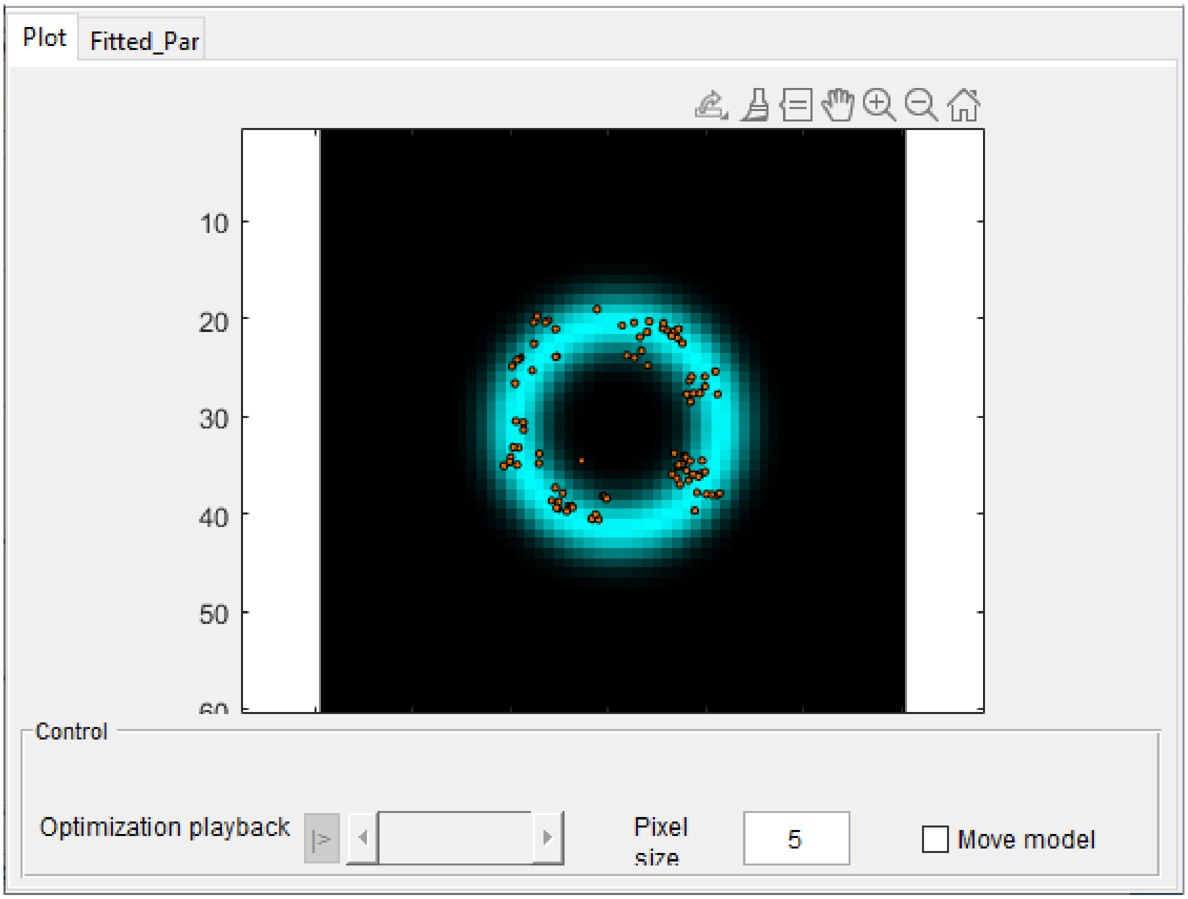

Note: The **LocMoFitGUI** window allows you to inspect the result right after fitting. The window may look different depending on the model type.

Now you have your first fit done! Congratulations! You should see the previously uncentered structure now centered because the fit finds the position of the structure. You can further explore a few sites to get familiar with the interface.

Next tutorial

You are in the introductory series. The next tutorial is *Composite model*.

5.1.2 Composite model

Note: Time required: ~15 min.

In *quick start*, we fitted a 2D ring to single nuclear pore complexes (NPCs). However, in 3D, there are actually two parallel rings per NPC (which can be seen in the side view). To extract parameters such as the distance between the two rings, a different geometry is required. Normally, this would require the user to create a new file with some coding. However, this particular geometry can be derived from the existing one (i.e., twice the 2D ring in 3D). Building a composite model by combining existing ones without coding is supported by LocMoFit.

Task

Building a composite model with the GUI. We will build a 3D dual-ring model by combining two times the identical ring model `ring3D`.

Requirement

- Software: **SMAP** installed. Further information can be found on our GitHub site.
- Localization data: *U2OS_Nup96_BG-AF647_demo_sml.mat*
- Fitting settings:
  - *dualRing_model1_fitPar.csv*

The data and setting files can be downloaded here.

Main tutorial

Preparation

1. Start SMAP (*how to?*).

Important: If you continue from the previous tutorial, please close the current SMAP and start a new session.

2. Load the dataset *U2OS_Nup96_BG-AF647_demo_sml.mat*. (*how to?*)
3. Go to **[ROIs]** -> **[Settings]**, click **show ROI manager**. This opens the **ROIManager** in a new window.
4. Load the plugin **LocMoFitGUI** in **[ROIs]** -> **[Evaluate]** (see *quick start* if you forget how to do it).

Setup

We will combine two identical rings in 3D (`ring3D`) to form a 3D dual-ring model.

1. We first load the individual models and set up the arguments of the model parameters. Now for the first ring model:
   - Go to **[M1]** -> **[Model]**, click the drop-down menu (where *select the model…* is shown), and then select *ring3D*. Click **load model**.
   - Go to the tab **[Parameters]** and click the button **Import**. In the new window, navigate to the settings directory and open *dualRing_model1_fitPar.csv*. Another new window should show up:

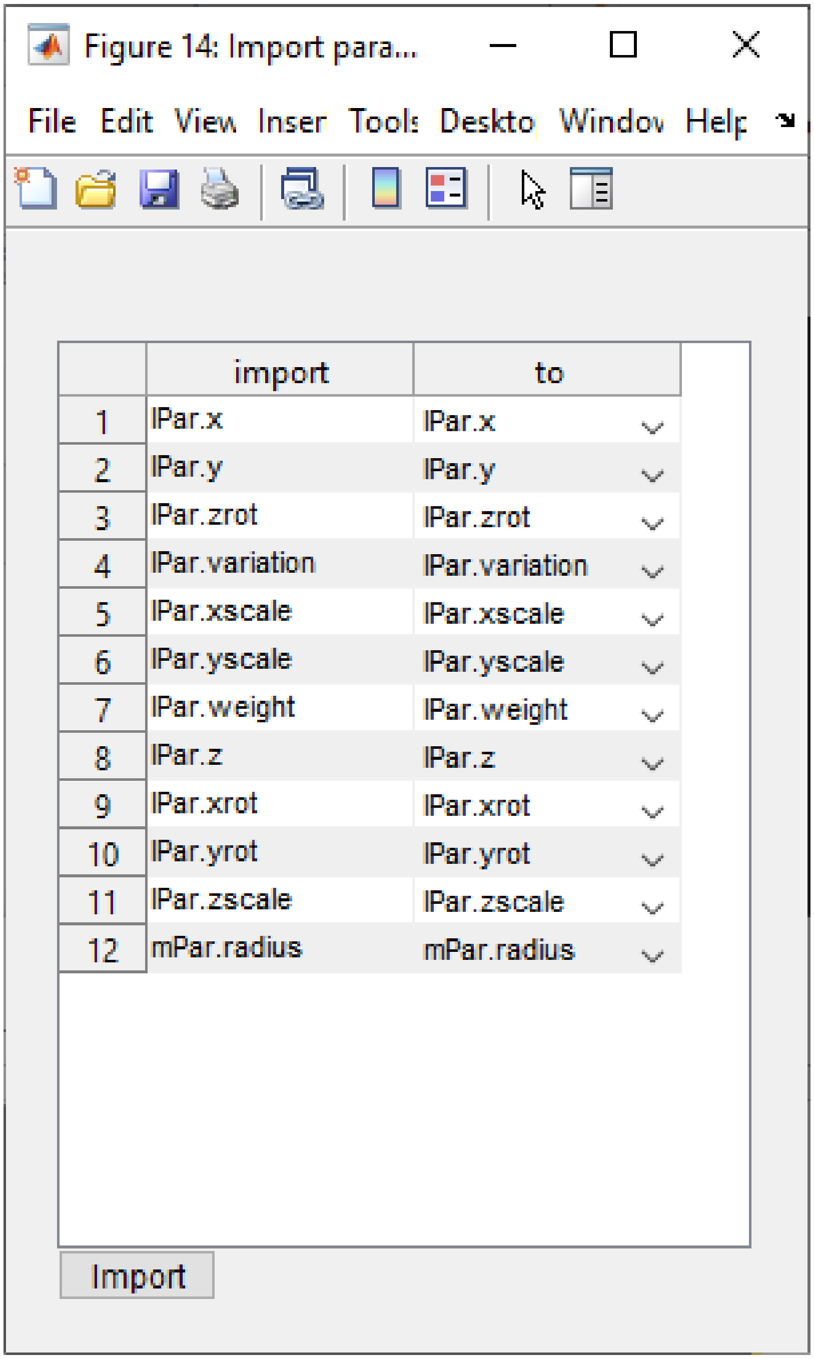

- Click **Import** button in the popup window. Now the parameter arguments/settings are updated.

Note: In the new window, the saved parameters (the field **import**) are matched to the parameters (the field **to**) in the GUI based on the same names and model types. We will discuss this more in step 2.

Hint: Now you loaded a previously exported parameter settings. In LocMoFit, you don't have to manually input the parameters every time. You can save the current settings through the **Export** button and use them for the next time.

2. Now you have to tell LocMoFit that we need to add a second model:

    - To add the second model tab **[M2]**, click **[+]** next to **[[x]]**:

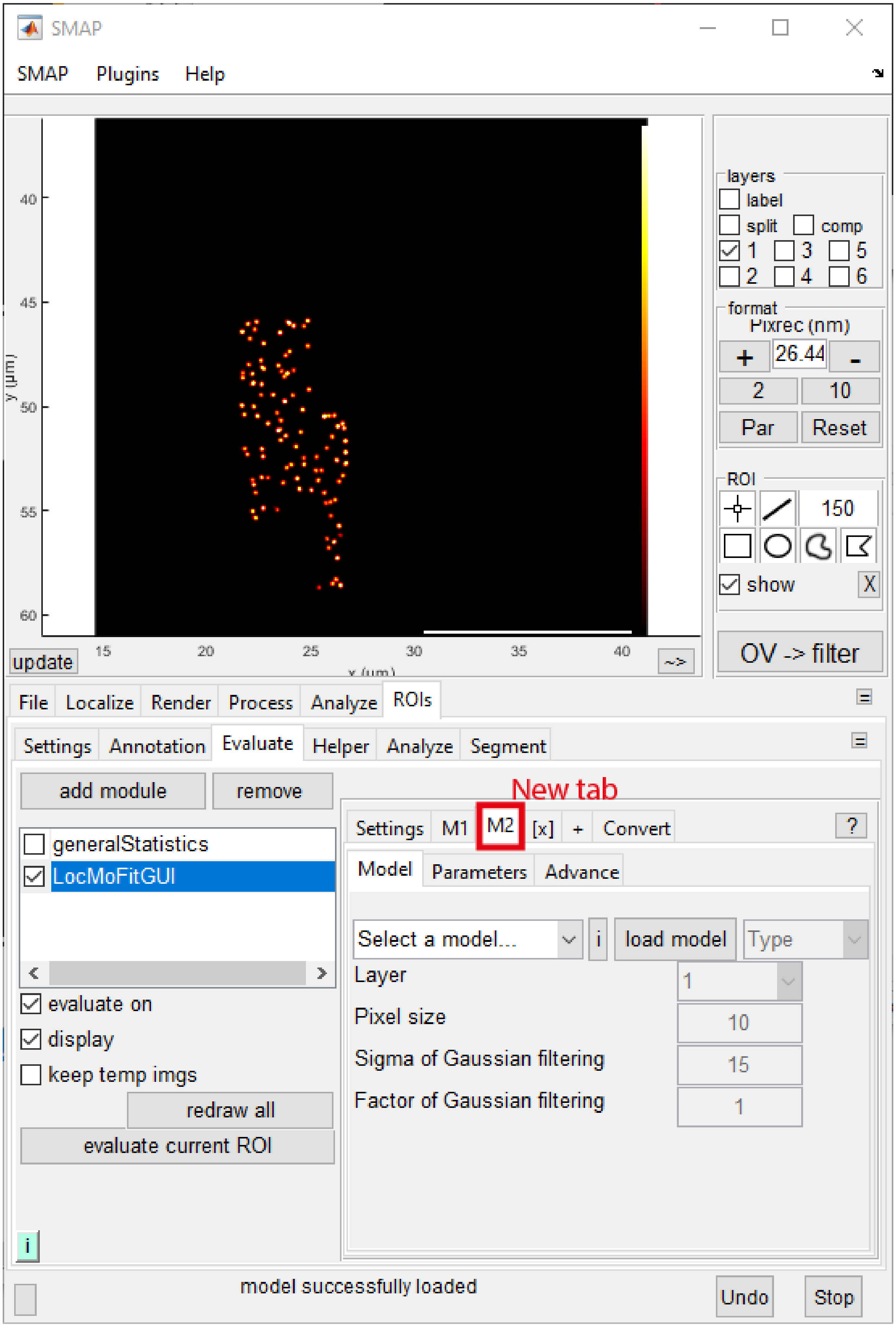
SMAP
SMAP Plugins Help
layers
label
split comp
1 3 5
2 4 6
format
Pixrec (nm)
26.44
2 10
Par Reset
ROI
150
show
OV -> filter
update
File Localize Render Process Analyze ROIs
Settings Annotation Evaluate Helper Analyze Segment
add module remove
New tab
generalStatistics
LocMoFitGUI
Settings M1 M2 [x] + Convert
Model Parameters Advance
Select a model... i load model Type
Layer
Pixel size
Sigma of Gaussian filtering
Factor of Gaussian filtering
evaluate on
display
keep temp imgs
redraw all
evaluate current ROI
model successfully loaded
Undo Stop

- You are now in **[M2]** -> **[Model]**. Click the drop-down menu (where *select the model…* is shown), select *ring3D*, and then click **load model**.
- Go to the tab **[Parameters]** and set all **fix** to *true* except for the parameter *z*. Next, change the **value** of *weight* to 1. Also change the **value**, **lb**, and **ub** of *z* to 40, -40, and 60. Now the table should look like this (non-default values are in bold):

name	value	fix	lb	ub	type	min	max
x	0	☑	-150	150	lPar	-150	150
y	0	☑	-150	150	lPar	-150	150
zrot	0	☑	-Inf	Inf	lPar	-Inf	Inf
variation	0	☑	0	10	lPar	0	20
xscale	1	☑	1	1	lPar	1	1
yscale	1	☑	1	1	lPar	1	1
weight	**1**	☑	1.0000e-05	1	lPar	1.0000e-05	1
z	**40**	☐	**-40**	**60**	lPar	-300	300
xrot	0	☑	-Inf	Inf	lPar	-Inf	Inf
yrot	0	☑	-Inf	Inf	lPar	-Inf	Inf
zscale	1	☑	1	1	lPar	1	1
radius	53.7000	☑	0	0	mPar	0	100

Important: Why are there so many zero values? Zeros mean that those parameters share the same values as M1. When there is more than one model, the extrinsic parameters (*lPar*) are always defined relative to the M1. For example, we set the **value** of *z* of M2 to 40 nm in order to move it 40 nm away from M1 in *z*, having the two rings separate.

3. To have better starting parameters, you may not want to always set the xy positions to zero. Instead, they can be roughly estimated based on the median position of the localizations. To take the median values as the starting parameters, you can use the functionality **Convert**, which dynamically converts from a *Rule* to the starting value of a parameter specified in **Target_fit**.

   - Go to tab **[Convert]** and fill in the table as the following (you can use the **+** button to add a new row):

Source	Rule	Target_fit	Target_usr
this step	median(locs.xnm)	m1.lPar.x	
this step	median(locs.ynm)	m1.lPar.y	
this step	median(locs.znm)-40	m1.lPar.z	

Note: Here we assign the median (xnm, ynm, and znm refer to the values on the respective coordinate axis, in nanometer) position of localizations (locs) as the initial parameters for the center position of model 1 (e.g., m1.lPar.x means the x position of model 1). Model 1 is additionally shifted 40 nm down in z.

4. Now preview the model *before fitting* for site 1 (see *quick start* if you forget how to do it). The expected result:

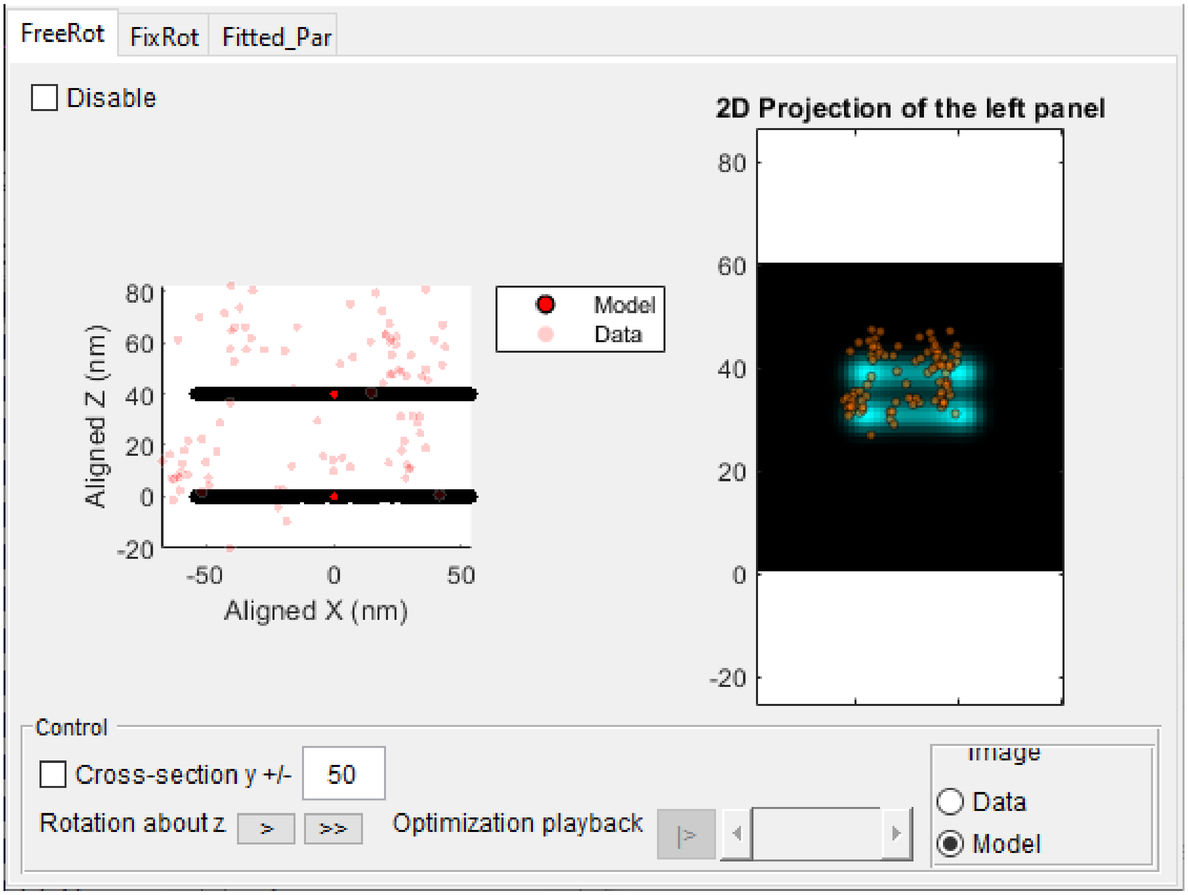

Tip: In the viewer, how do I explore the model in 3D? In the scatter plot on the left, you can either hold the left-click and drag or right-click and select views:

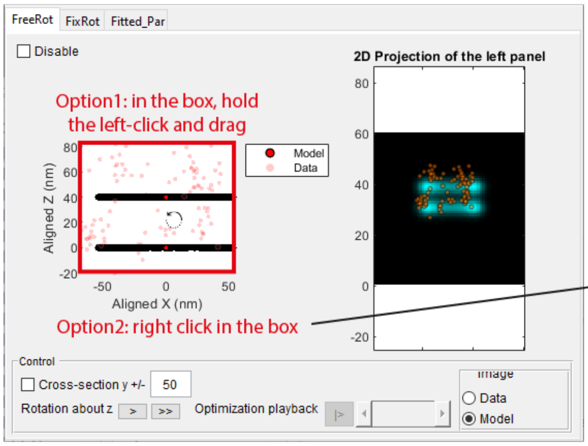

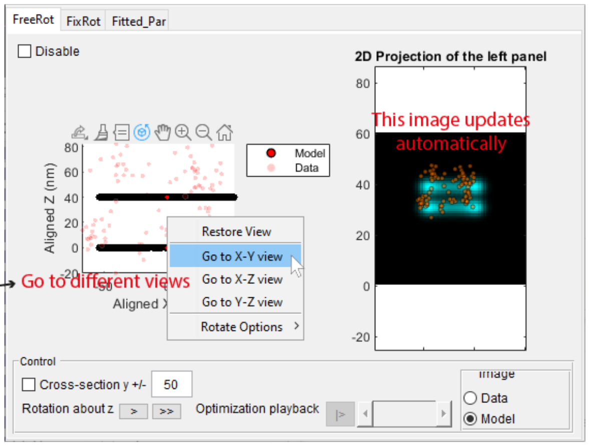

Go to X-Y view, you will see the side view of the model:

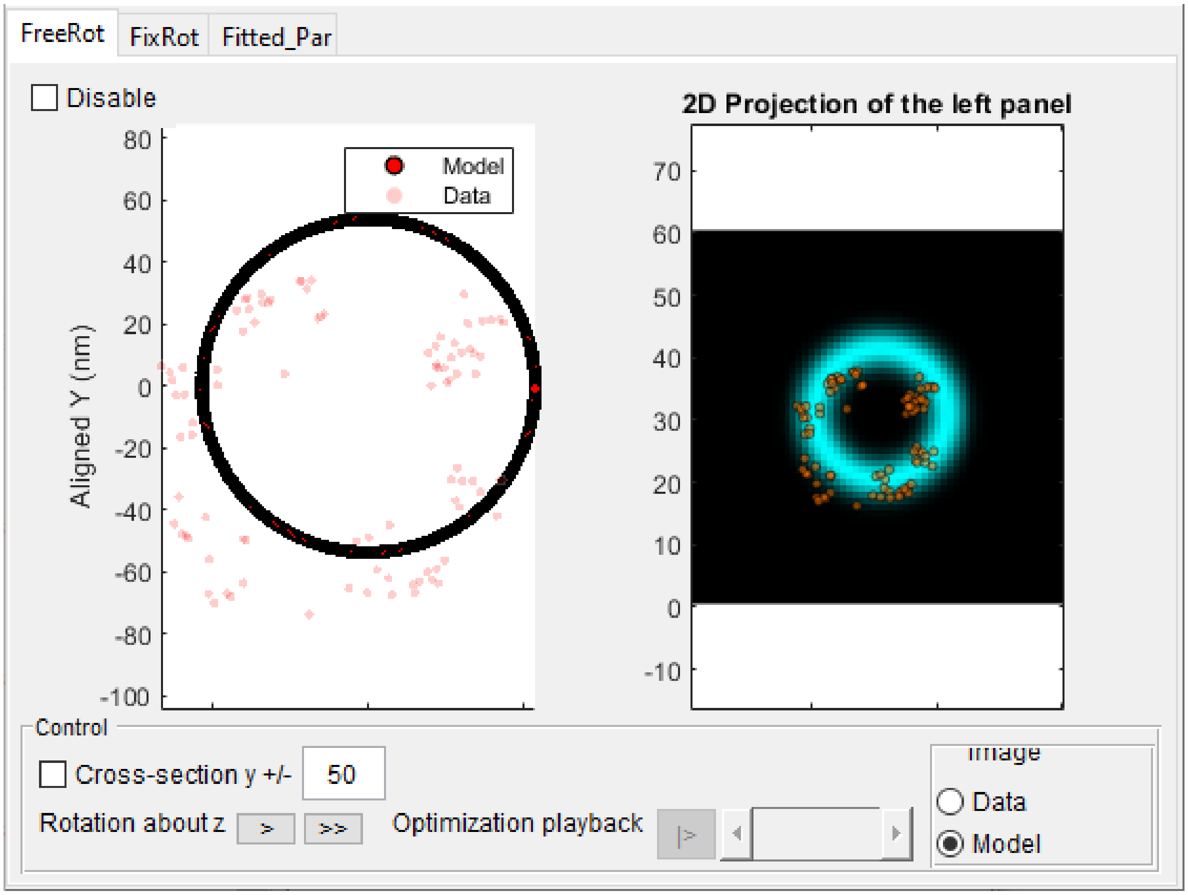

Fitting

1. To disable the preview mode, uncheck **preview** in the tab **[Parameters]**.
2. In the *ROI Manager* window, click on one site and wait for a few seconds. You should see the updated *LocMoFitGUI* window displaying the fitted model:

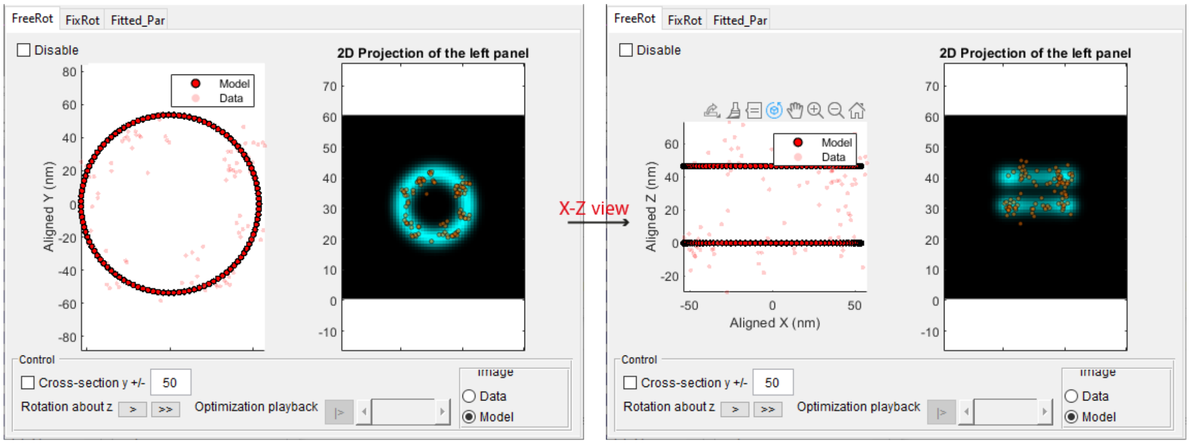

Note: To see the effect of fitting, you can compare the model before and after fitting with/without the preview mode on.

Saving all settings

You can save the current settings, including the loaded models, parameter settings, and converter, for the same task next time:

- Go to **[Settings]**, click on the button *save*, navigate to where you want to save the settings, and save it as *NPC3D_step1_dualRings_LocMoFit.mat*:

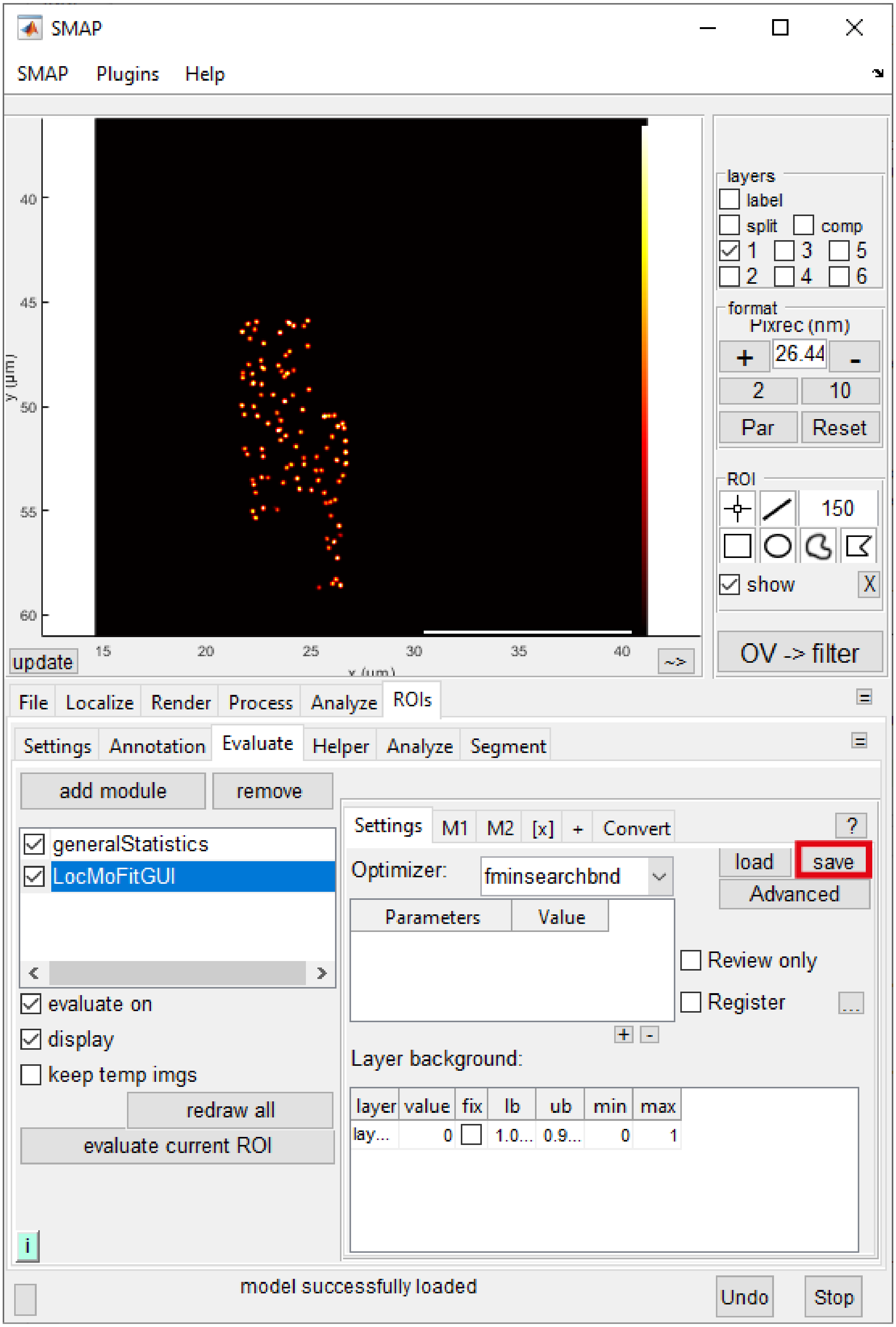
SMAP
SMAP Plugins Help
layers
label
split comp
format
Pixrec (nm)
26.44
Par Reset
ROI
150
show
OV -> filter
update
File Localize Render Process Analyze ROIs
Settings Annotation Evaluate Helper Analyze Segment
add module
remove
generalStatistics
LocMoFitGUI
evaluate on
display
keep temp imgs
redraw all
evaluate current ROI
Settings M1 M2 [x] + Convert
Optimizer:
fminsearchbnd
load
save
Advanced
Parameters
Value
Review only
Register
Layer background:
layer value fix lb ub min max
model successfully loaded
Undo
Stop

Congratulations! Now you retained the settings of the composite model you built for the first time.

Next tutorial

You are in the introductory series. The next tutorial is *Chaining steps*

5.1.3 Chaining steps

Note: Time required: ~20 min.

Note: In this tutorial, we loaded the pre-defined parameter settings to simplify the procedure. For all the structures we fit in our manuscript, we provide optimized settings. However, when you work on a new structure, you have to tweak the settings yourself and modify the **parameter table** (find out more here).

Task

Chaining fitting steps with the GUI. We will set up two fitting steps with different models, using the results of the previous step as the initial parameters for the next one.

Requirement

- Software: **SMAP** installed. Further information can be found on our GitHub site.
- Localization data: *U2OS_Nup96_BG-AF647_demo_sml.mat*
- Fitting settings:
  - *NPC3D_step1_dualRings_LocMoFit.mat* (the one you saved in the last tutorial)
  - *NPC3D_step2_dualRings_freeRadius_LocMoFit.mat*
  - *NPC3D_step3_points_LocMoFit.mat*

Files can be downloaded here.

Important: Please first finish the tutorial *composite model*.

Main tutorial

Preparation

1. Start SMAP (*how to?*).

   Important: If you continue from the previous tutorial, please close the current SMAP and start a new session.

2. Load the dataset *U2OS_Nup96_BG-AF647_demo_sml.mat*. (*how to?*)

3. Go to **[ROIs]** -> **[Settings]**, click **show ROI manager**. This opens the **ROIManager** in a new window.

Loading LocMoFit

Now you need three instances of LocMoFit:

- Go to **[Evaluate]** tab and click on **add module**.
- In the popup window, select *LocMoFitGUI* and click **ok**.
- Repeat the two steps above twice more.

Now you should see the **LocMoFitGUI**, **LocMoFitGUI_2**, and **LocMoFitGUI_3** in the loaded modules. These are for three different fitting steps respectively.

Setup

Next, we load the LocMoFit settings for fitting three different models (step 1: `ring3D`; step 2: `dualRing3D_discrete`; step 3: also `dualRing3D_discrete` but with only 32 points).

- Step 1 fits the composite dual-ring model (template) you built earlier to detect the ring separation, position, and orientation of the NPC.
- Step 2 fits a single dual-ring model with a free radius to detect it and to fine-tune other parameters.
- Step 3 fits a point model to detect the ring twist. To set them up, follow the steps below:

1. For step 1, click *LocMoFitGUI* in the list of loaded modules.
2. On the right panel, go to **[Settings]**, click **load**, navigate to the settings directory, and select *NPC3D_step1_dualRings_LocMoFit.mat* (which you saved earlier).
3. For step 2, click *LocMoFitGUI_2* in the list of loaded modules.
4. On the right panel, go to **[Settings]**, click **load**, navigate to the settings directory, and select *NPC3D_step2_dualRings_freeRadius_LocMoFit.mat*.
5. For step 3, click *LocMoFitGUI_3* in the list of loaded modules.
6. On the right panel, go to **[Settings]**, click **load**, navigate to the settings directory, and select *NPC3D_step3_points_LocMoFit.mat*.
7. Go to tab **[Convert]**, click **Match**. You should see a new window:

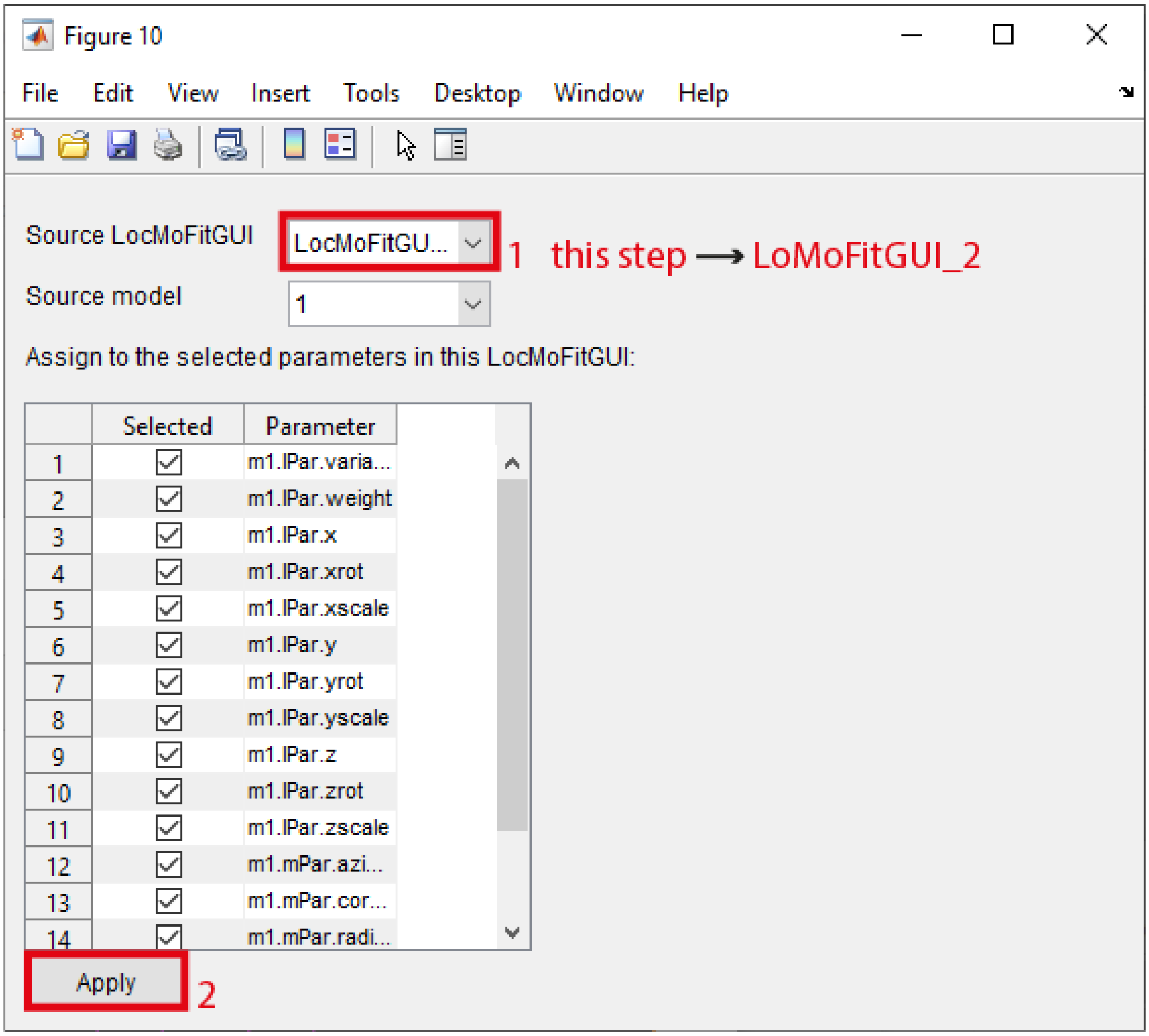

Note: What is this popup window for? It allows you to pass the parameter estimations from the previous steps to the current step as initial parameters based on the names of parameters.

---

1. In the popup window, in **Source LocMoFitGUI**, change the selection from *this model* to *LocMoFitGUI_2* and the click **Apply**. Now you should see new information filled in tab **[Convert]**:

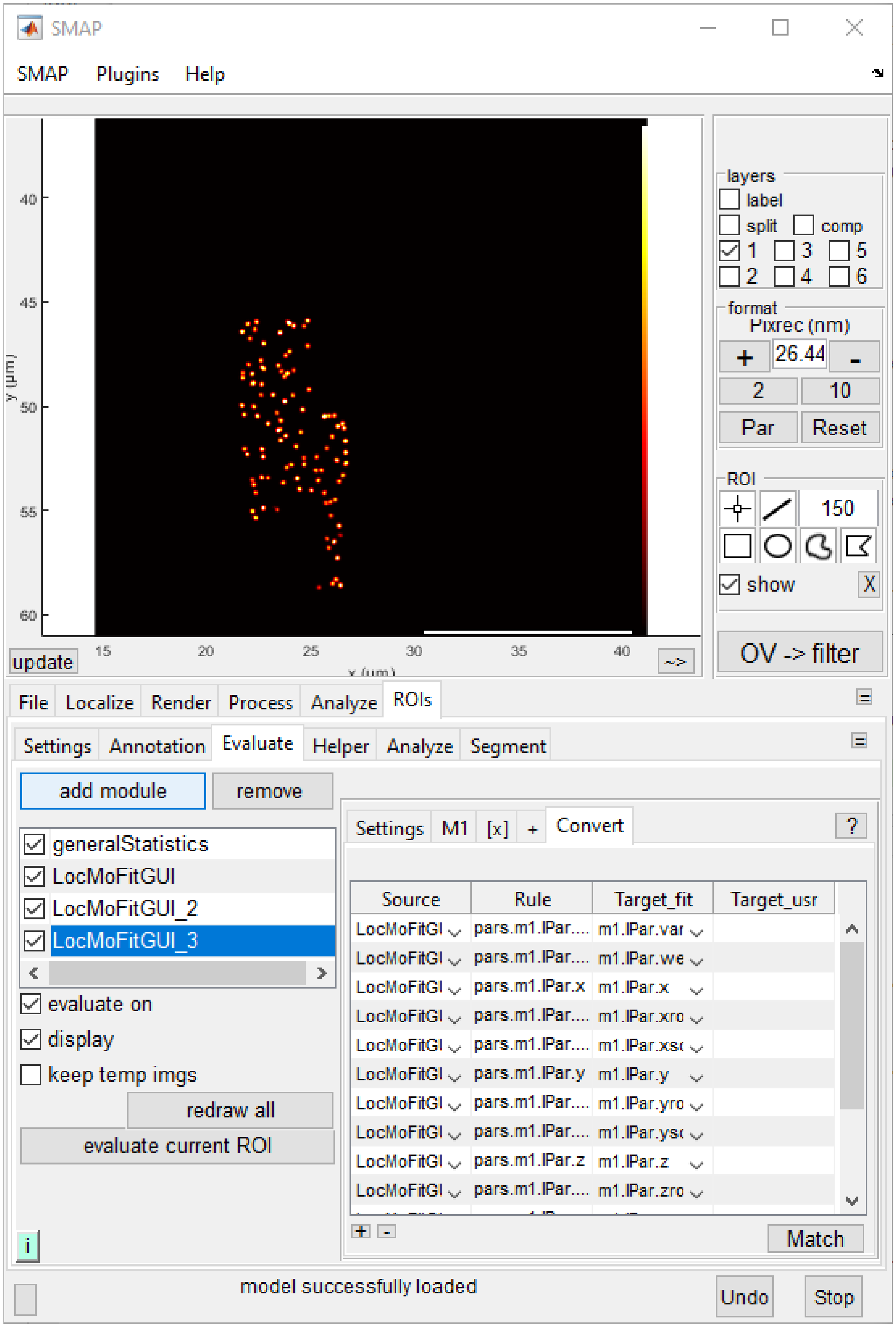
SMAP
SMAP Plugins Help
layers
label
split comp
1 3 5
2 4 6
format
Pixrec (nm)
26.44
2 10
Par Reset
ROI
150
show
OV -> filter
update
y (µm)
x (µm)
File Localize Render Process Analyze ROIs
Settings Annotation Evaluate Helper Analyze Segment
add module remove
generalStatistics
LocMoFitGUI
LocMoFitGUI_2
LocMoFitGUI_3
evaluate on
display
keep temp imgs
redraw all
evaluate current ROI
Settings M1 [x] + Convert ?
Source Rule Target_fit Target_usr
Match
model successfully loaded
Undo Stop

Note: **[Convert]** can be used to convert the fitted values in the previous step to an initial parameter of the current step. It calculates values according to the **rules** based on its **source** and then writes the values to the **target_fit** (see the column names of the convert table). For example, you just defined to assign the parameter m1.mPar.ringDistance in this step from the **Source** *LocMoFitGUI* based on the value calculated by the **Rule** pars.m2.lPar.z.

m1.mPar.ringDistance and *pars.m2.lPar.z* are IDs of the corresponding parameters. m1.mPar.ringDistance means the parameter *ringDistance*, which is an intrinsic parameter (*mPar*) of model 1 (*m1*). See the syntax that can be used in **[convert]**.

Fitting

1. Click on site 1 in the *ROI manager* window. Now you should see three viewers, one for each step:
    - X-Y view

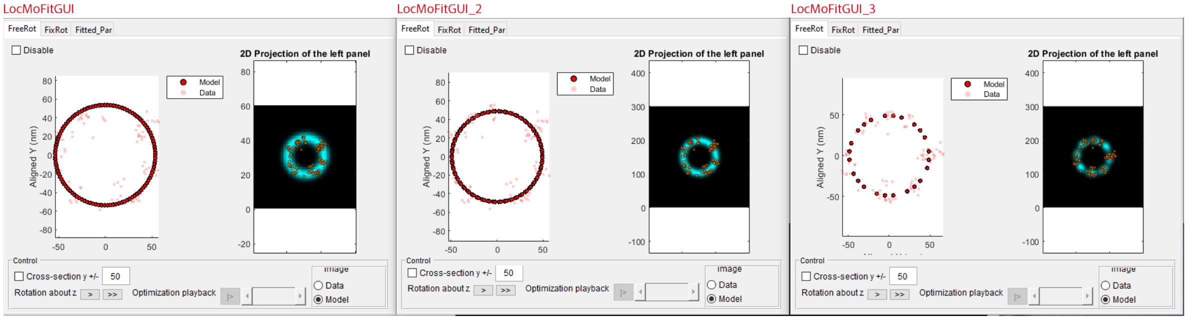

    - X-Z view

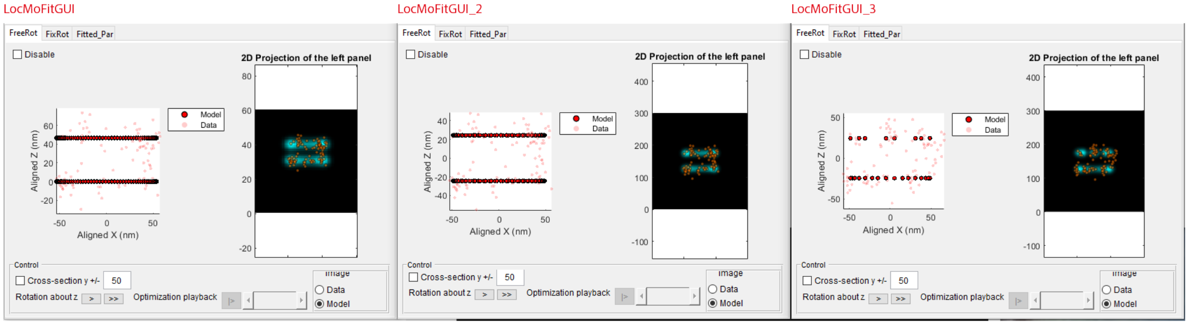

2. You can further explore a few sites to get familiar with the interface.

Batch fitting

After inspecting several sites, we are now moving on to fit all sites. Such a batch analysis can be executed with the *redraw all* function to perform the fitting site-by-site:

1. To disable the viewers for efficiency, stay in the **[Evaluate]** tab. In the left panel, uncheck **display**:

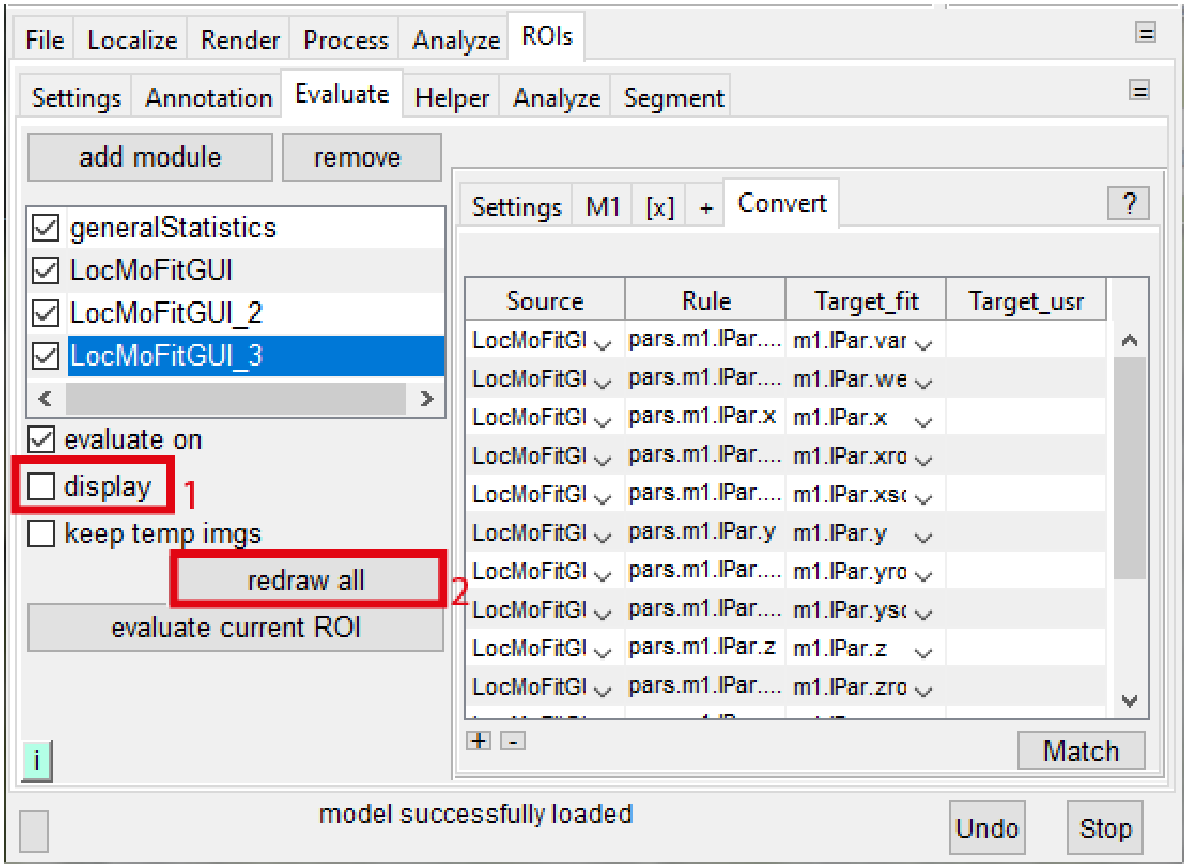

2. Click **redraw all** as shown above. You will see the analysis going down the ROI list.

Note:

- While running, you should see the message *"redrawall: site [current site] of 100"* in the status bar.
- You will know the analysis is done when *"redrawall: completed"* shows up in the status bar. This usually takes around 7 minutes.

Summary of parameter values

When the **redraw all** is done, all the fit results have been saved in the SMAP session. To show the summary of parameter values, you can use the SMAP plugin *summarizeModFitNPC3D*:

1. Go to the drop-down menu **[Plugins]** -> **[ROIManager]** -> **[Analyze]** -> **[*summarizeModFitNPC3D*]**:

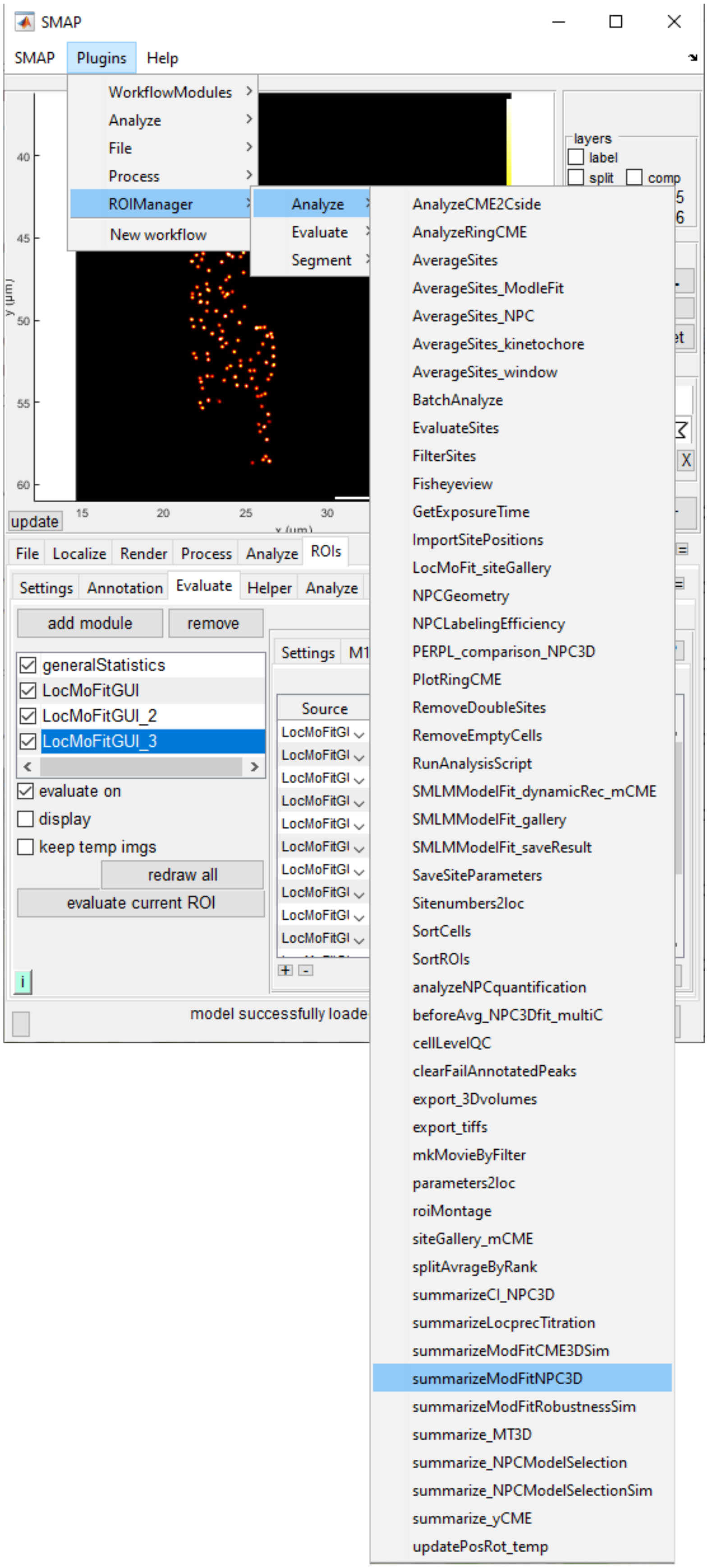
SMAP
SMAP
Plugins
Help
WorkflowModules
Analyze
File
Process
ROIManager
New workflow
Analyze
Evaluate
Segment
AnalyzeCME2Cside
AnalyzeRingCME
AverageSites
AverageSites_ModleFit
AverageSites_NPC
AverageSites_kinetochore
AverageSites_window
BatchAnalyze
EvaluateSites
FilterSites
Fisheyeview
GetExposureTime
ImportSitePositions
LocMoFit_siteGallery
NPCGeometry
NPCLabelingEfficiency
PERPL_comparison_NPC3D
PlotRingCME
RemoveDoubleSites
RemoveEmptyCells
RunAnalysisScript
SMLMModelFit_dynamicRec_mCME
SMLMModelFit_gallery
SMLMModelFit_saveResult
SaveSiteParameters
Sitenumbers2loc
SortCells
SortROIs
analyzeNPCquantification
beforeAvg_NPC3Dfit_multiC
cellLevelQC
clearFailAnnotatedPeaks
export_3Dvolumes
export_tiffs
mkMovieByFilter
parameters2loc
roiMontage
siteGallery_mCME
splitAvrageByRank
summarizeCl_NPC3D
summarizeLocprecTitration
summarizeModFitCME3DSim
summarizeModFitNPC3D
summarizeModFitRobustnessSim
summarize_MT3D
summarize_NPCModelSelection
summarize_NPCModelSelectionSim
summarize_yCME
updatePosRot_temp
layers
label
split
comp
update
File
Localize
Render
Process
Analyze
ROIs
Settings
Annotation
Evaluate
Helper
Analyze
add module
remove
generalStatistics
LocMoFitGUI
LocMoFitGUI_2
LocMoFitGUI_3
evaluate on
display
keep temp imgs
redraw all
evaluate current ROI
Settings
Source
model successfully loade

2. Click **Run** in the new window:

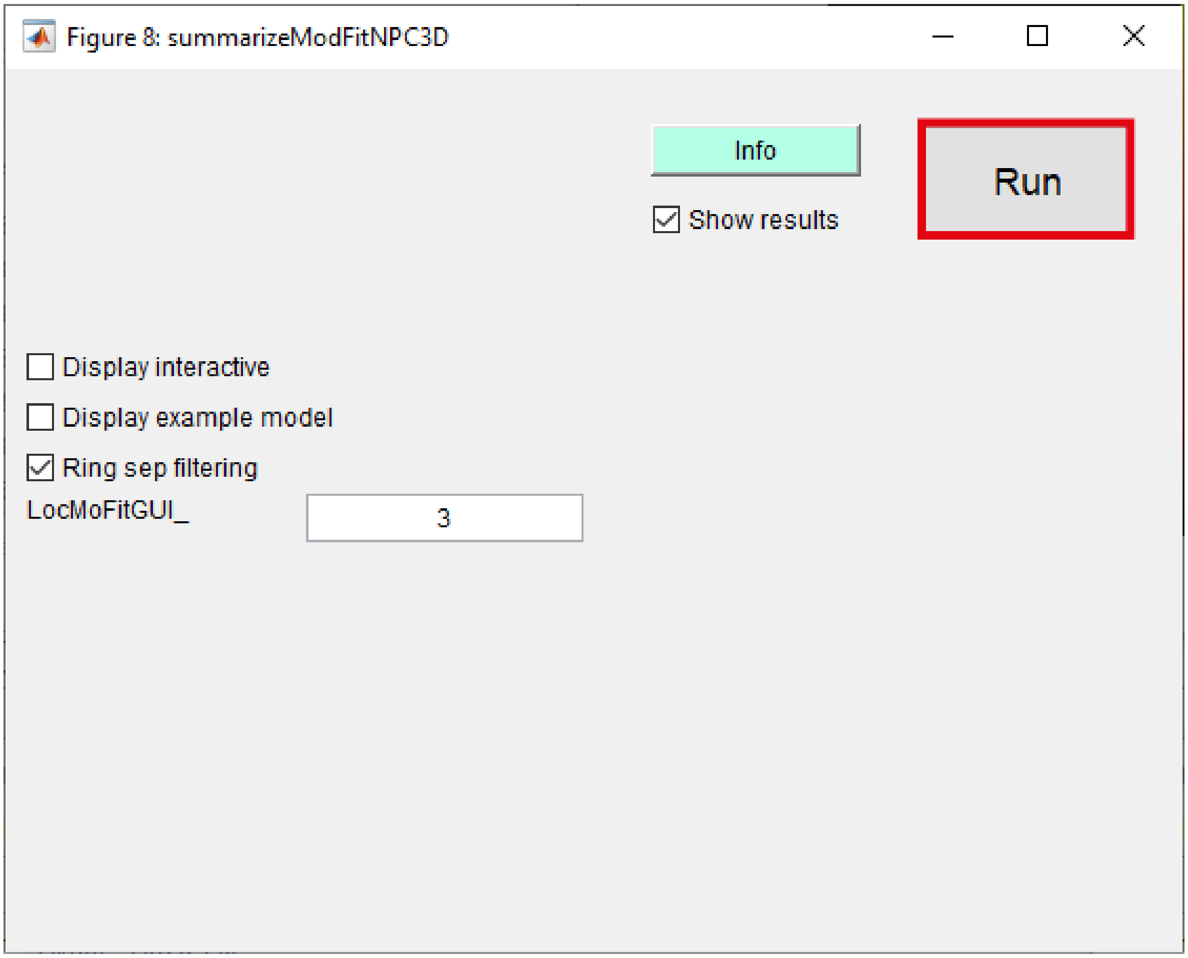

The window where a scatter plot and histograms are will then pop up:

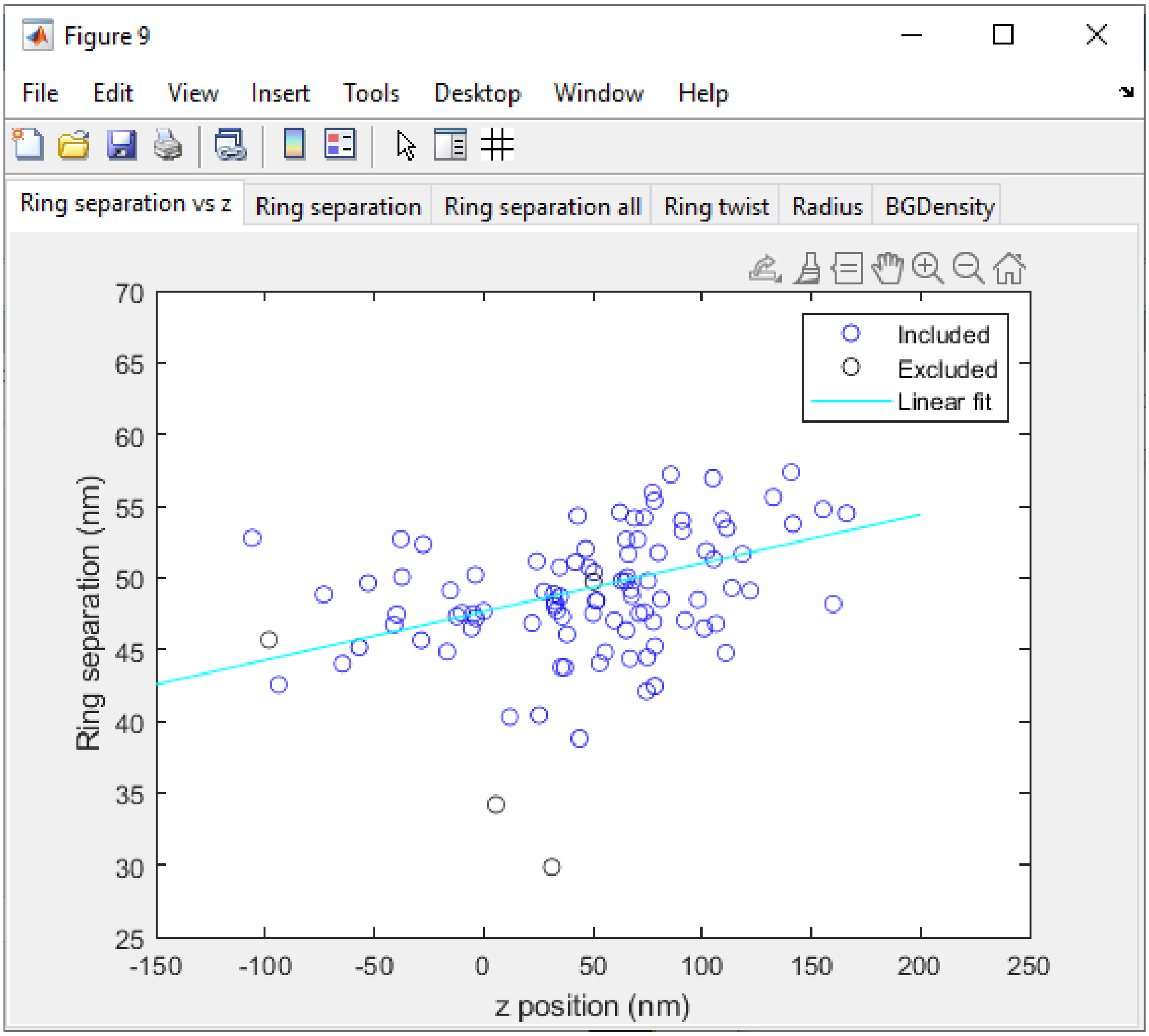

The tabs are:

- **Ring separation vs z**: a scatter plot showing the correlation between the z position and ring separation of the NPCs.
- **Ring separation**: a histogram of the ring separation (one-ring sites excluded).
- **Ring separation all**: a histogram of the ring separation (all sites).
- **Ring twist**: a histogram of the ring twist (one-ring sites excluded).
- **Radius**: a histogram of the radius (one-ring sites excluded).
- **BGDensity**: a histogram of the background density (one-ring sites excluded).

Now you have reproduced the workflow we applied in the manuscript to extract key structural parameters of single NPCs!

The end of the introductory series

This tutorial is the end of the introductory series. If you started from *quick start* and followed along the *Next tutorial* section in each tutorial, you should have learned the basics of LocMoFit.

CHAPTER

SIX

SIMULATING SMLM DATA

Note: Time required: ~10 min.

In this tutorial, you will learn how to simulate SMLM data based on a geometric model.

6.1 Requirement

- Software: SMAP installed. Further information can be found on our GitHub site.

6.2 Task

Simulating realistic localization data based on a geometric model.

6.3 Main tutorial

6.3.1 Preparation

You have to load the geometric model for generating localizations. We use LocMoFitGUI as the interface for setting up the model. Here we use the point model `dualRing3D_discrete` of the nuclear pore complex (NPC) as an example:

1. Start SMAP (*how to?*).
2. Load a new instance of the module *LocMoFitGUI* in tab **[ROIs]** -> **[Evaluate]** (see *quick start* to recap).
3. Uncheck **evaluate on** in the left panel:

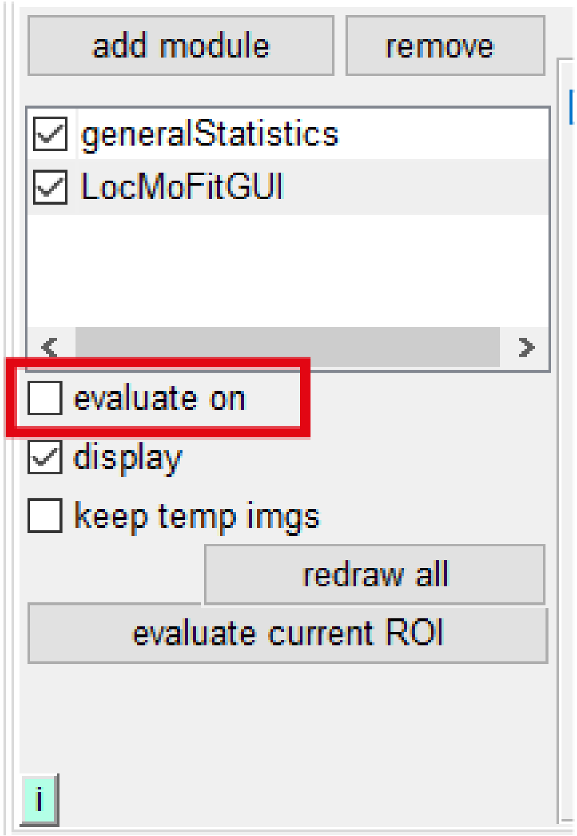

4. Load the particular model and set up the settings for the model parameters:
   - Go to **[M1]** -> **[Model]**, click the drop-down menu (where *select the model…* is shown), and then select `dualRing3D_discrete`.

6.3.2 Simulation

Once the model is loaded, we have to pass it on to the simulation engine provided by SMAP:

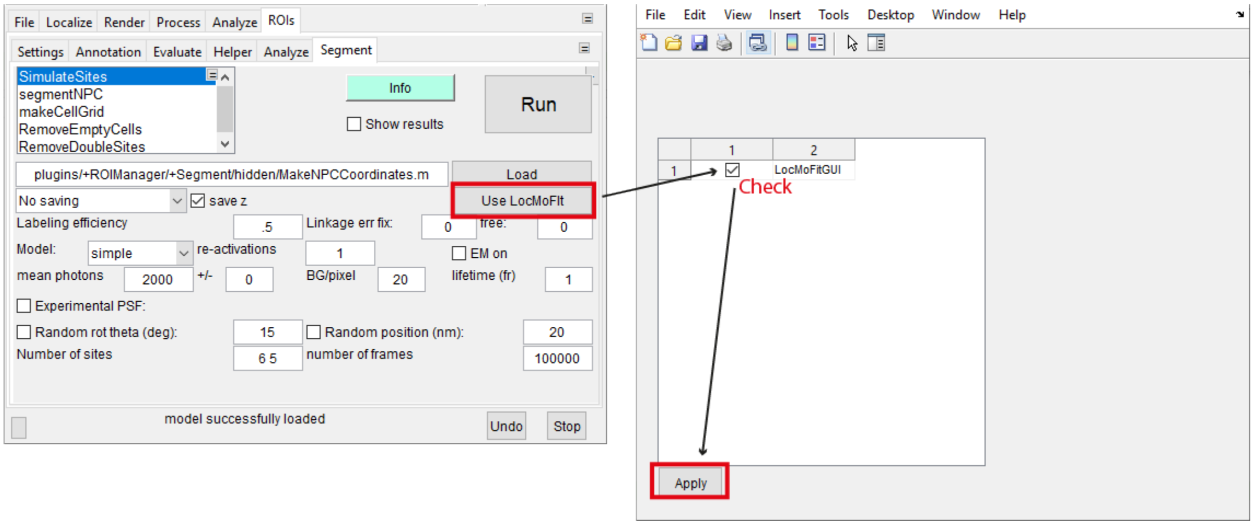

1. We first connect the engine to the model:

- Go to [ROIs]->[Segment], click *SimulateSites* in the list of plugins:
- Click **Use LocMoFit** and check *LocMoFitGUI* in the new window.
- Click **Apply**.

LocMoFitGUI is successfully loaded if the new button **Set model pars** shows up.

2. Click **Set model pars**. A new window will show up and allow you to specify parameter values. Here we keep the default values so do not have to change anything:

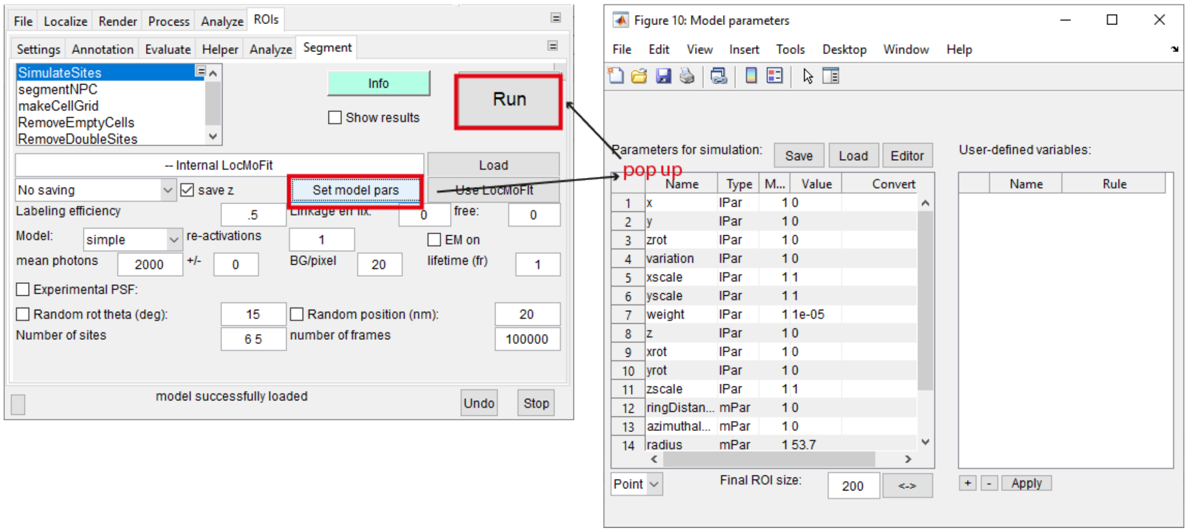

Note: How do I specify parameter values?

- **For specifying a fixed value**, enter it in field **Value** for a parameter (can be identified in the columns *Name*, *Type*, and *Model*).
- **For a random value sampled in a defined interval**, enter two values (e.g., '*-15 15*') separated by a space in field **Value**. In this example, the parameter will be a random value between -15 and 15.

---

3. Usually, we have to set up the SMLM properties in tab **[SimulateSites]**. For simplicity, we keep the default values.

Hint: You can hover over the respective properties in the tab to show their definitions.

---

4. Click **Run** in **[SimulateSites]**. The simulation is done when *'ROIManager.Segment.SimulateSites finished'* is displayed in the status bar. This usually takes a few seconds:

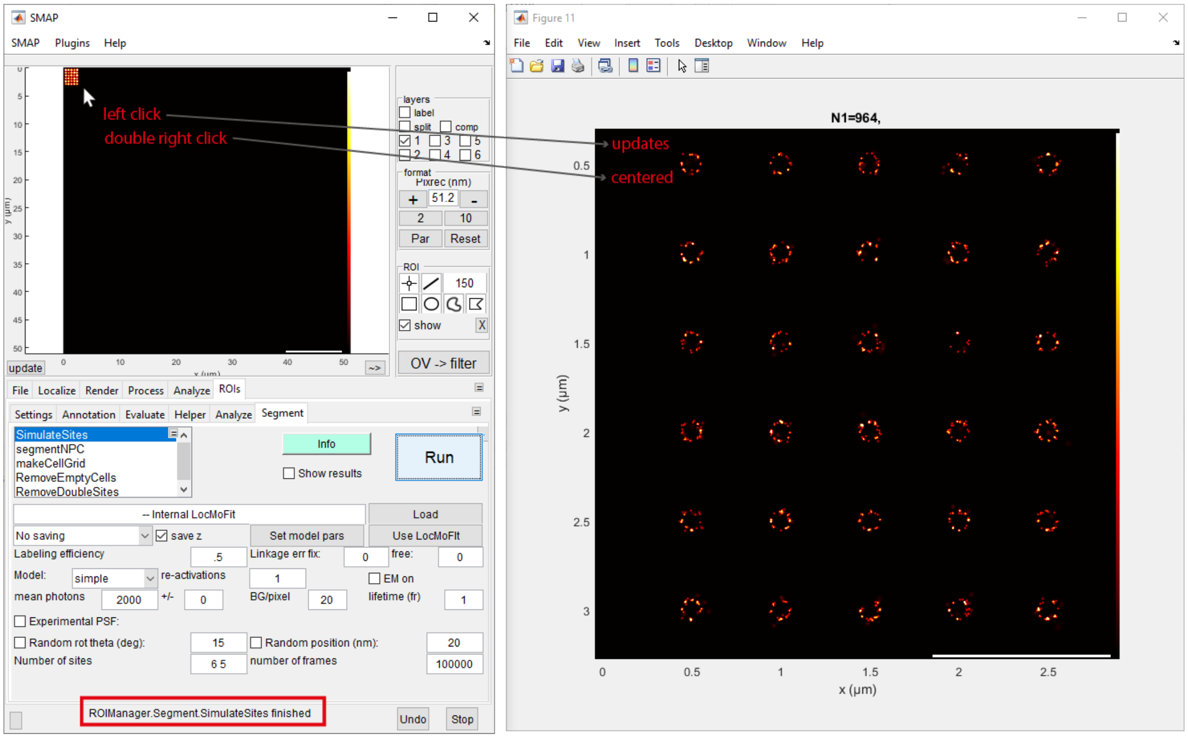

Now you can explore the overview of the simulated data. You can interact with the overview with a left click or right click. You can deal with the simulated data as with real data.

Note: How do I keep the simulated data? You can save it through button **Save** in tab **[File]**:

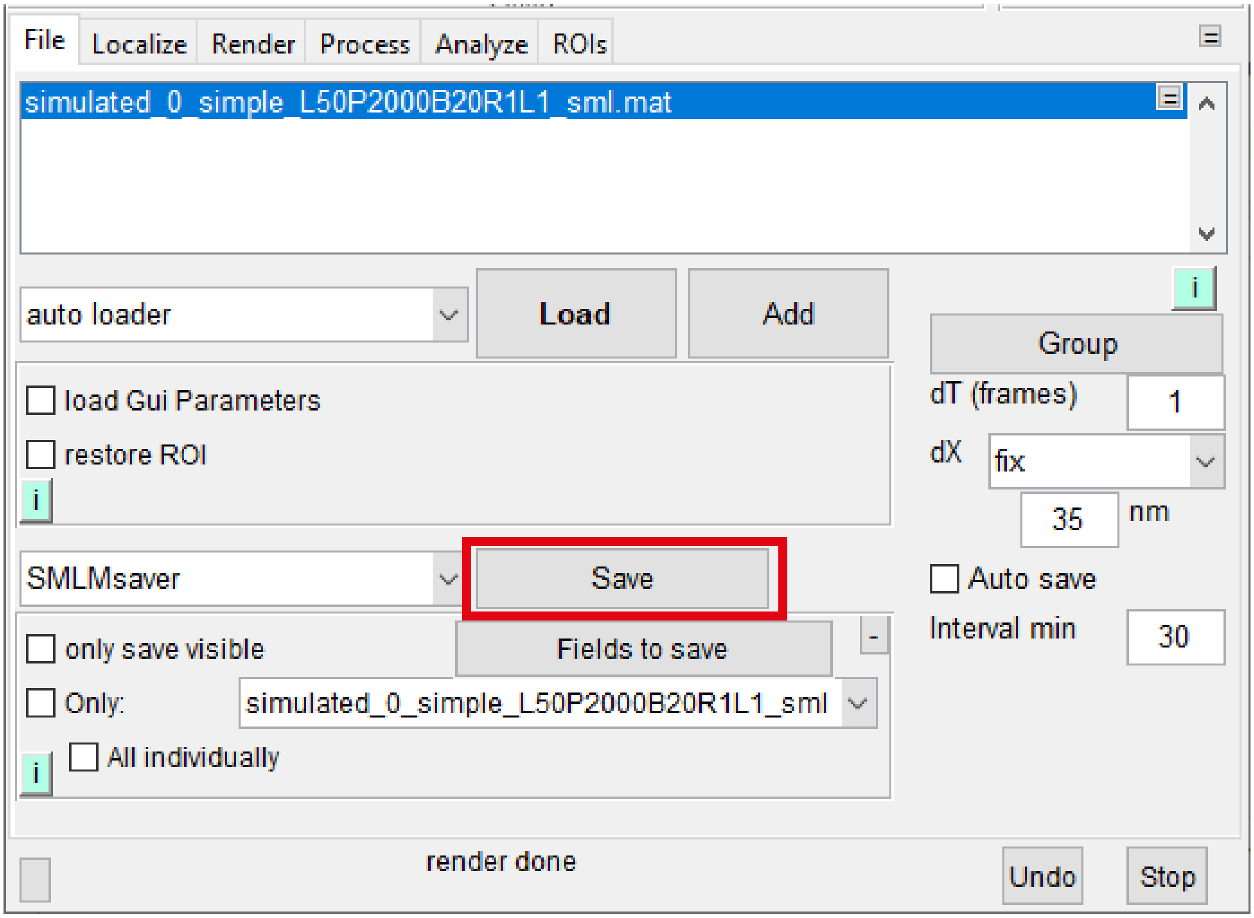

This is the end of the tutorial.

Part IV

FAQ

CHAPTER

SEVEN

HOW-TO

7.1 SMAP basics

7.1.1 Run SMAP

In MATLAB

Simply enter *SMAP* in the *MATLAB Command Window*:

```
SMAP
```

Compiled version

You can run SMAP through the *SMAP* shortcut in the operating system's start menu.

If you successfully open SMAP, you should see its main GUI with *all initialized* displayed in the **status bar**:

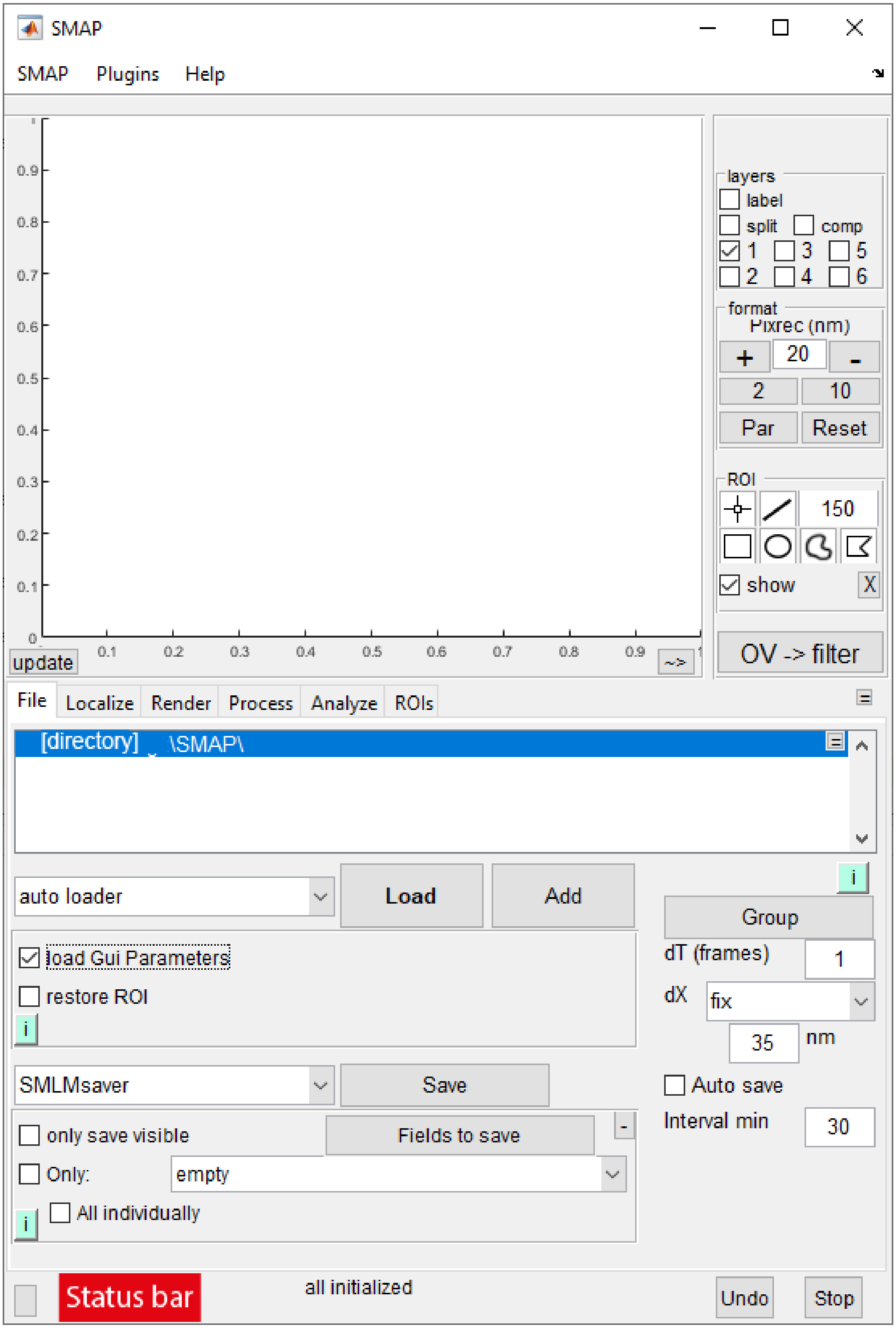
SMAP
SMAP Plugins Help
update
layers
label
split comp
1 3 5
2 4 6
format
Pixrec (nm)
20
2 10
Par Reset
ROI
150
show
OV -> filter
File Localize Render Process Analyze ROIs
[directory] \SMAP\
auto loader
Load
Add
load Gui Parameters
restore ROI
Group
dT (frames) 1
dX fix
35 nm
SMLMsaver
Save
Auto save
only save visible
Fields to save
Interval min 30
Only: empty
All individually
Status bar
all initialized
Undo
Stop

7.1.2 Data loading in SMAP

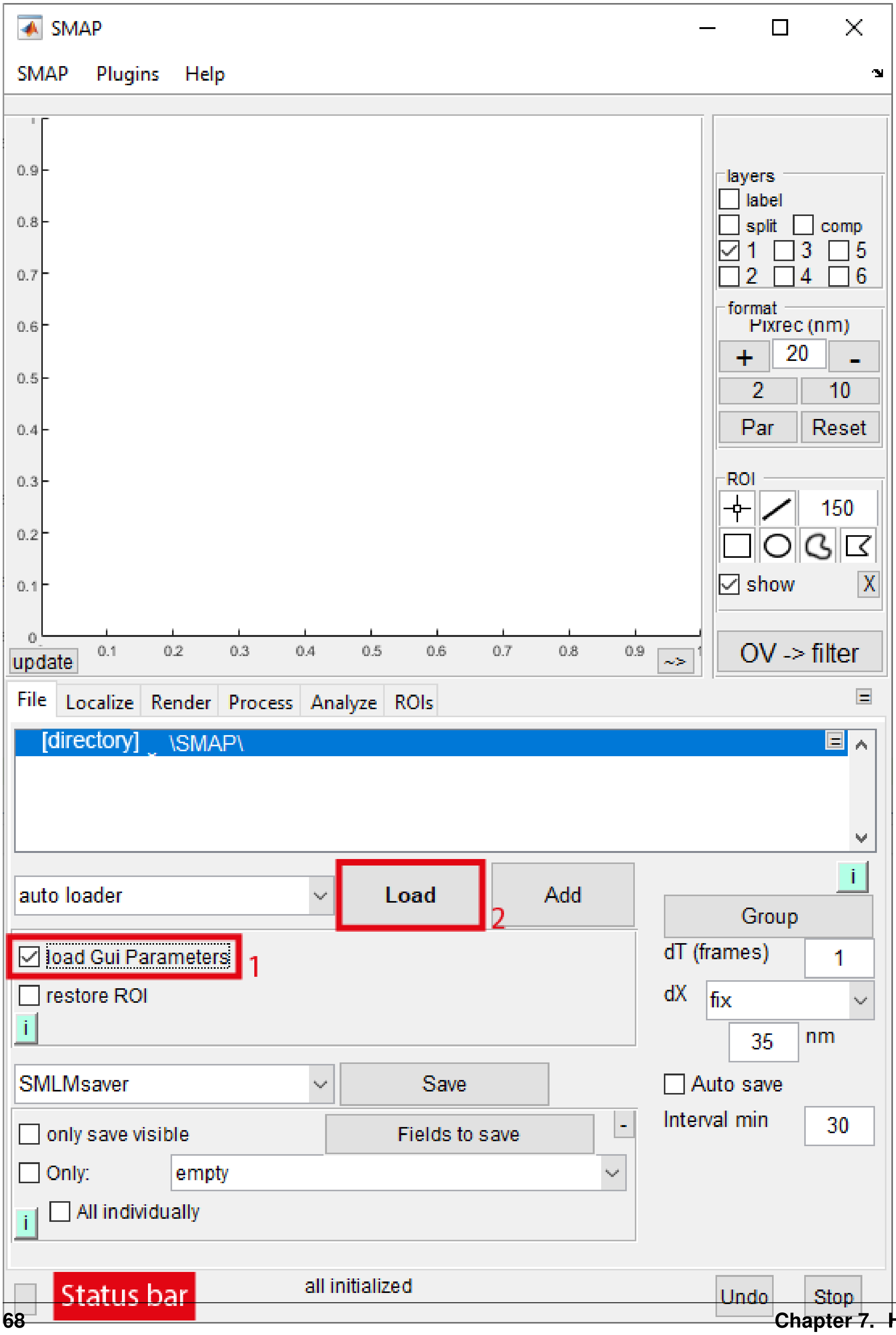

1. Check *load Gui Parameters*.

Note: With this, the previously saved Gui Parameters in the file will be also loaded and applied. Otherwise the SMAP session keeps the original parameters.

2. Click **load**, navigate to where the localization data are saved, and select the files to be loaded. Wait until *loading done* is displayed in the status bar:

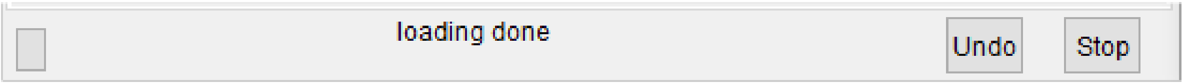

Now the file(s) is loaded.

7.1.3 Load LocMoFit GUI in SMAP

1. Go to the **[ROIs]** tab.
2. Go to **[Evaluate]** tab and click **add module**.

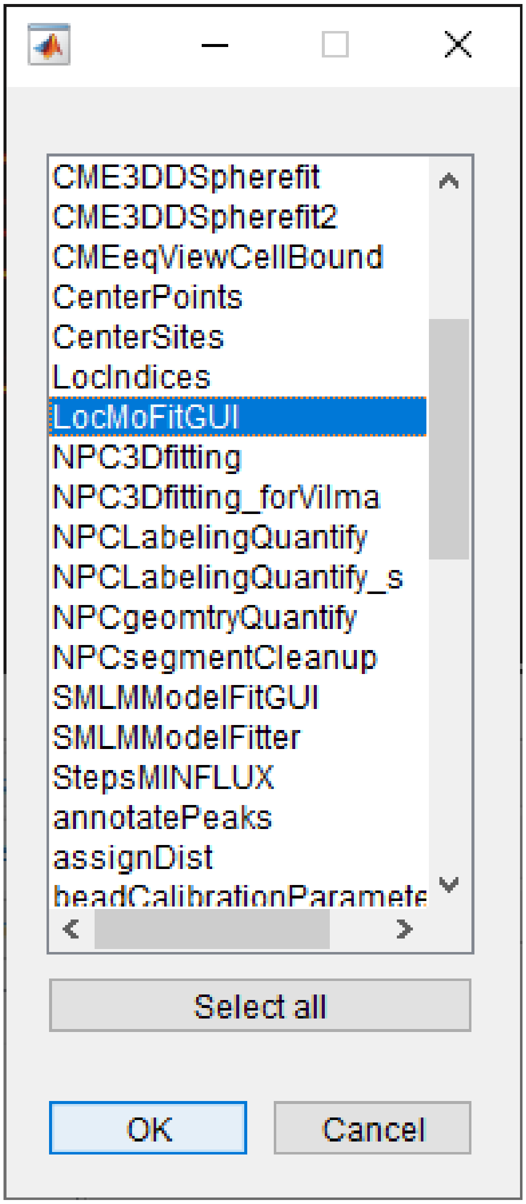

3. In the popup window, select *LocMoFitGUI* and click *ok*.
4. Show the *LocMoFitGUI* GUI by clicking on it in the list of loaded modules. Your SMAP window should look like this now:

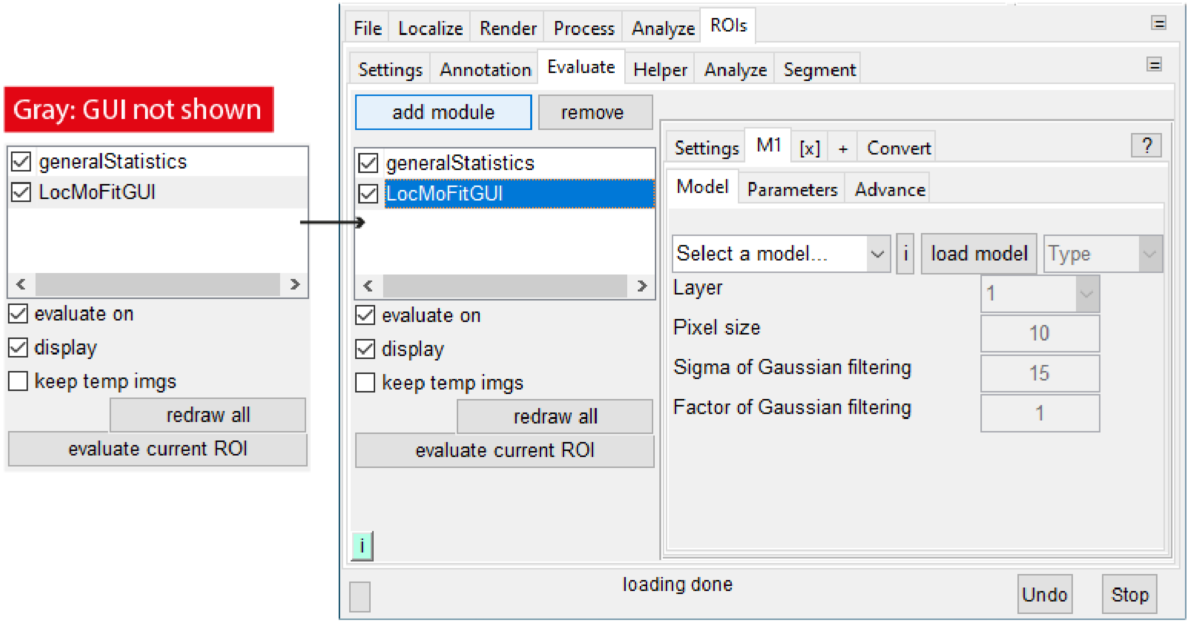
Gray: GUI not shown
generalStatistics
LocMoFitGUI
evaluate on
display
keep temp imgs
redraw all
evaluate current ROI
File
Localize
Render
Process
Analyze
ROIs
Settings
Annotation
Evaluate
Helper
Analyze
Segment
add module
remove
generalStatistics
LocMoFitGUI
evaluate on
display
keep temp imgs
redraw all
evaluate current ROI
Settings
M1
[x]
+
Convert
?
Model
Parameters
Advance
Select a model...
i
load model
Type
Layer
1
Pixel size
10
Sigma of Gaussian filtering
15
Factor of Gaussian filtering
1
i
loading done
Undo
Stop

7.2 LocMoFit GUI

7.2.1 Load a model in LocMoFit GUI

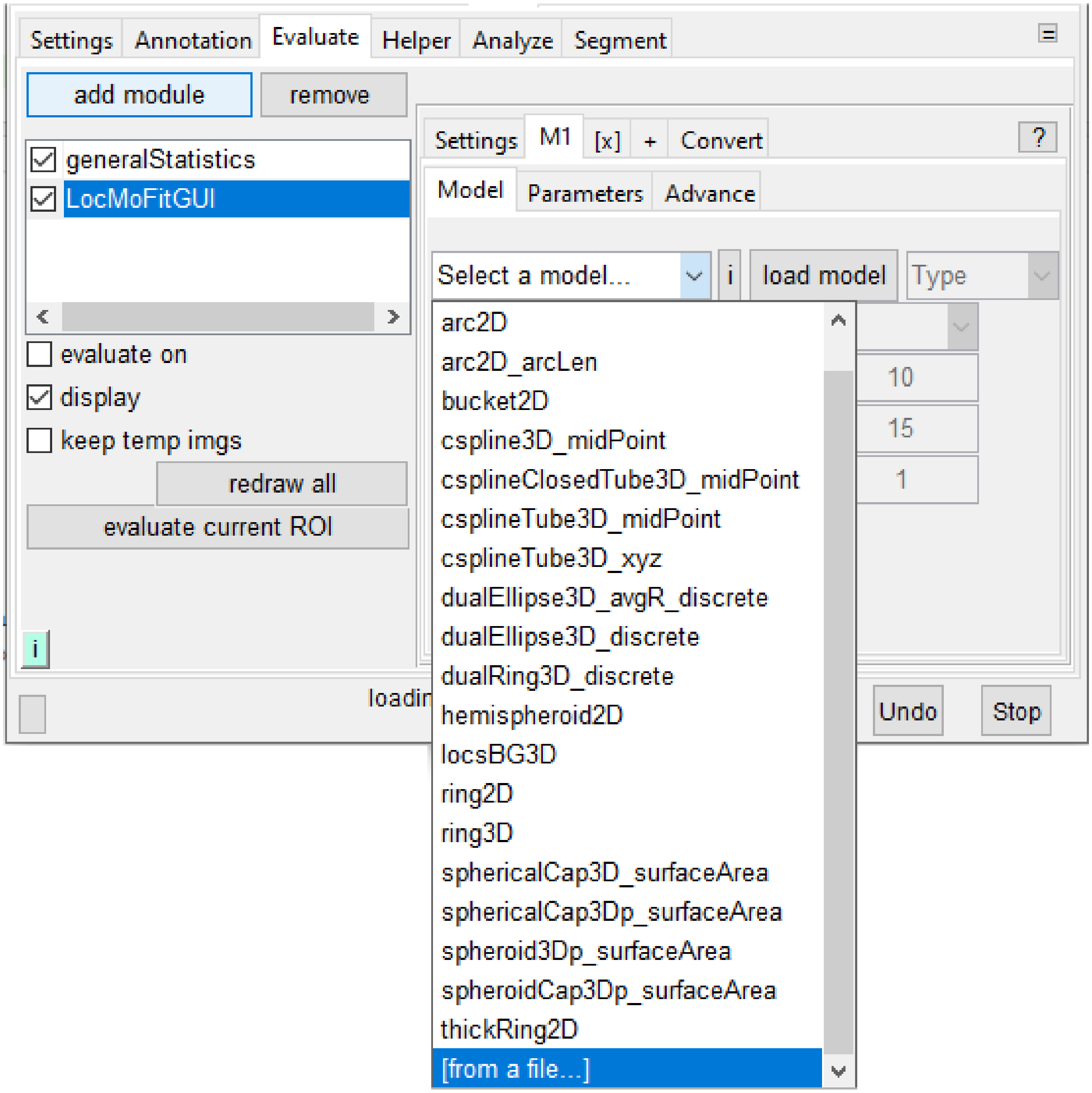

Built-in models

1. Go to a model tab (**[M1]** or **[M2]**… etc.) -> **[Model]**.
2. Click the drop-down menu (where *selet the model…* is shown).
3. Select the model to be loaded, and then click **load model**. The possible options of models are documented in the *page of model library*.

Own models

1. Go to a model tab (**[M1]** or **[M2]**… etc.) -> **[Model]**.
2. Click the drop-down menu (where *selet the model…* is shown).
3. Select the option *[from a file…]* at the bottom, and then click **load model**. Navigate to where the model you want to load is and load the model.

Note: **Possible formates of a model** include .png and .mat for images and .m for functions.

> **Warning:** Loading .m files is not supported by the compiled version of SMAP.

Part V

Documentation

CHAPTER

EIGHT

REFERENCES

This page details all the classes, methods, and functions of the framework.

8.1 Model library

All the names of the models follow the rule: **'[geometry][dimension][p]_[parameterization]_[…]_[model form]'**, with the simplest case as '[geometry][dimension]' (e.g., `ring3D`). For example, `dualEllipse3D_avgR_discrete` means a dual-ellipse geometry in 3D, parametrized by the average radius, in a discrete form.

Note: The individual components of the names

- [geometry]: the geometry.
- [dimension]: the model dimension.
- [p]: (optional) when it is mentioned, the model is parametric, otherwise not.
- [parameterization]: (optional) when multiple parameterizations exist, this is added to identify the specific parameterization. When the [parameterization] is not mentioned, the model is assumed to be with the default parameterization.
- […]: (optional) if the [parameterization] itself is not sufficient to differentiate the implementations, extra labels are added here.
- [model form]: (optional) the form of how the model is implemented. When it is not provided, the model is in the continuous form.

8.1.1 Arc

class models.**arc2D**(*varargin*)

Bases: `@geometricModel.geometricModel`

`arc2D` is a 2D model that describes an arc geometry.

Geometric parameters:

- *radius*: (nm) the radius of the ring where the arc is derived.
- *theta*: (degree) the closing anlge of the arc.

Relavent biological structure:

- Cross-section of a clathrin coat

Preview:

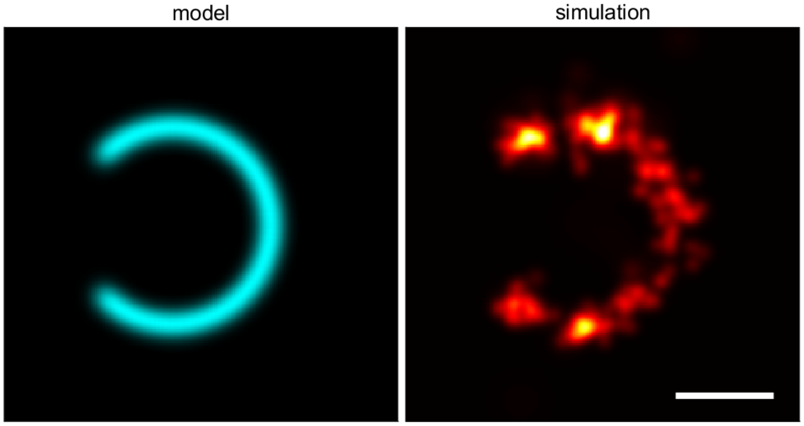

Scale bar: 50 nm.

reference (*par*, *dx*)
For details, see `reference()`.

class models.**arc2D_arcLen** (*varargin*)
Bases: `@geometricModel.geometricModel`

`arc2D_arcLen` is a 2D model that describes an arc geometry. It describes the same geometry as by `arc2D` but with a different parameterization.

Geometric parameters:

- *arcLength*: (nm) the length of the arc.
- *theta*: (degree) the closing anlge of the arc.

Relavent biological structure:

- Cross-section of a clathrin coat

See also:

`arc2D`

reference (*par*, *dx*)
For details, see `reference()`.

8.1.2 Bucket

class models.**bucket2D** (*varargin*)
Bases: `@geometricModel.geometricModel`

`bucket2D` is a 2D model that describes a bucket geometry. It creates a bucket based on an arc. The bucket is created to contain the arc.

Geometric parameters:

- *radius*: (nm) the radius of the ring where the arc is derived.
- *theta*: (°) the closing anlge of the arc.

See also:

`arc2D`

reference (*par*, *dx*)
For details, see `reference()`.

getDerivedPars (*pars*)
For details, see *getDerivedPars()*.

8.1.3 Ring

class models.**ring2D** (*varargin*)
Bases: *@geometricModel.geometricModel*

ring2D is a 2D model that describes a ring geometry.

Geometric parameters:

- *radius*: (nm) the ring radius.

Relavent biological structure:

- Top-view projections of the nuclear pore complex.

Preview:

Note: It will be available soon.

reference (*par*, *dx*)
For details, see *reference()*.

class models.**ellipse3D** (*varargin*)
Bases: *@geometricModel.geometricModel*

ellipse3D describes a ellipse geometry in 3D.

Geometric parameters:

- *a*: (nm) the axis along the x-axis.
- *b*: (nm) the axis along the y-axis.

Preview:

Note: It will be available soon.

reference (*par*, *dx*)
For details, see *reference()*.

class models.**ring3D** (*varargin*)
Bases: *@geometricModel.geometricModel*

The model class *ring3D* describes a ring geometry in 3D.

Geometric parameters:

- *radius*: (nm) the ring radius.

Relavent biological structure:

- Top-view projections of the nuclear pore complex.

Preview:

Note: It will be available soon.

reference (*par*, *dx*)
For details, see `reference()`.

8.1.4 Spline

class models.**cspline3D_midPoint** (*varargin*)
Bases: `@geometricModel.geometricModel`

A c-spline for describing a linear structure traversing in 3D space.

Geometric parameters:

- *xMid, yMid, zMid*: (nm) the xyz coordinates of the mid point.
- *dist*: (nm): the distance between neighbouring control points
- *rotAzi[L/R]_n, rotEle[L/R]_n*: (°) the azimuthal and elevation

angles or of the vector pointing to the [L/R]_n control point. *[L/R]* is either L (left) or R (right) with respect to the mid point. *n* indicates the order. For exmple, rotAziL1 means the azimuthal angle defining the 1st point on the left of the mid point.

Relavent biological structure:

- Actin filaments
- The central axes of microtubules

Preview:

> **Note:** It will be available soon.

reference (*par*, *dx*)
For details, see `reference()`.

getThings2Plot (*par*)
The user can define what should be also displayed in the plots.

Parameters **obj** – a `@geometricModel.geometricModel` object.

Returns *items* – things to be plotted.

8.1.5 Tube and derivitives

class models.**csplineClosedTube3D_midPoint** (*varargin*)
Bases: `@geometricModel.geometricModel`

A c-spline for describing a flexible tube that has its ends closed, traversing in 3D space.

Geometric parameters:

- *xMid, yMid, zMid*: (nm) the xyz coordinates of the mid point.
- *r*: (nm) the radius of the tube.
- *dist*: (nm): the distance between neighbouring control points
- *rotAzi[L/R]_n, rotEle[L/R]_n*: (°) the azimuthal and elevation angles or of the vector pointing to the [L/R]_n control point. *[L/R]* is either L (left) or R (right) with respect to the mid point. *n* indicates the order. For exmple, rotAziL1 means the azimuthal angle defining the 1st point on the left of the mid point.

Relavent biological structure:

- outer membrane of a mitochondria

Preview:

Note: It will be available soon.

reference (*par*, *dx*)
For details, see *reference()*.

getThings2Plot (*par*, *varargin*)
The user can define what should be also displayed in the plots.

Parameters **obj** – a *@geometricModel.geometricModel* object.

Returns *items* – things to be plotted.

getDerivedPars (*pars*)
Exports a empty variable when no derived parameters.

class models.**csplineTube3D_midPoint** (*varargin*)
Bases: *@geometricModel.geometricModel*

A c-spline for describing a flexible tube traversing in 3D space.

Geometric parameters:

- *xMid, yMid, zMid*: (nm) the xyz coordinates of the mid point.
- *r*: (nm) the radius of the tube.
- *dist*: (nm): the distance between neighbouring control points
- *rotAzi[L/R]_n, rotEle[L/R]_n*: (°) the azimuthal and elevation angles or of the vector pointing to the [L/R]_n control point. *[L/R]* is either L (left) or R (right) with respect to the mid point. *n* indicates the order. For exmple, rotAziL1 means the azimuthal angle defining the 1st point on the left of the mid point.

Relavent biological structure:

- actin filaments
- the central axes of microtubules

Preview:

Note: It will be available soon.

reference (*par*, *dx*)
For details, see *reference()*.

getThings2Plot (*par*, *varargin*)
The user can define what should be also displayed in the plots.

Parameters **obj** – a *@geometricModel.geometricModel* object.

Returns *items* – things to be plotted.

getDerivedPars (*pars*)
Exports a empty variable when no derived parameters.

class models.**csplineTube3D_xyz**(*varargin*)

Bases: *@geometricModel.geometricModel*

A c-spline for describing a linear structure traversing in 3D space. It describe the same geometry as *csplineTube3D_midPoint* but with a different parameterization.

Geometric parameters:

- *x_n, y_n, z_n*: (nm) the xyz coordinates of the nth control point.
- *r*: (nm) the radius of the tube.

Relavent biological structure:

- Actin filaments
- The central axes of microtubules

See also:

csplineTube3D_midPoint

Preview:

Note: It will be available soon.

reference(*par*, *dx*)

set additional parameters of the model

getThings2Plot(*par*)

The user can define what should be also displayed in the plots.

Parameters obj – a *@geometricModel.geometricModel* object.

Returns *items* – things to be plotted.

8.1.6 Dual rings and derivitives

class models.**dualEllipse3D_discrete**(*varargin*)

Bases: *@geometricModel.geometricModel*

dualEllipse3D_discrete describes two parallel ellipse in 3D. The ellipse have the same xy positions and short/long axes.

Geometric parameters:

- *ringDistance*: (nm) the distance between the two parallel rings.
- *azimuthalShift*: (°) the twist angle between the two parallel rings.
- *a*: (nm) the length of the long axis.
- *ellipticity*: (no unit) or *e*, is defined as *e = 1-b/a*, where a and b are the long and short axes.
- *aDir*: (°) the rotational offset between the first corner and the long axis *a*.
- *cornerDegree*: (°) the rotational offset between two copies per corner.

Relavent biological structure:

- deformed nuclear pore complex

Preview:

> **Note:** It will be available soon.

reference (*par*, *dx*)
For details, see *reference()*.

class models.**dualEllipse3D_avgR_discrete** (*varargin*)
Bases: *@geometricModel.geometricModel*

dualEllipse3D_avgR_discrete describes two parallel ellipse in 3D. The ellipse have the same xy positions and short/long axes. It describe the same geometry as *dualEllipse3D_discrete* but with a different parameterization.

Geometric parameters:

- *ringDistance*: (nm) the distance between the two parallel rings.
- *azimuthalShift*: (°) the twist angle between the two parallel rings.
- *avgR*: (nm) the average of the short and long axes.
- *ellipticity*: (no unit) or *e*, is defined as *e = 1-b/a*, where a and b are the long and short axes.
- *aDir*: (°) the rotational offset between the first corner and the long axis *a*.
- *cornerDegree*: (°) the rotational offset between two copies per corner.

Relavent biological structure:

- deformed nuclear pore complex

See also:

dualEllipse3D_discrete

Preview:

> **Note:** It will be available soon.

reference (*par*, *dx*)
For details, see *reference()*.

class models.**dualRing3D_discrete** (*varargin*)
Bases: *@geometricModel.geometricModel*

dualRing3D_discrete describes two parallel rings in 3D. The rings have the same xy position and radius.

Geometric parameters:

- *ringDistance*: (nm) the distance between the two parallel rings.
- *azimuthalShift*: (°) the twist angle between the two parallel rings.
- *radius*: (nm) the radius of the rings.
- *cornerDegree*: (°) the rotational offset between two copies per corner.

Relavent biological structure:

- the nuclear pore complex

Preview:

Note: It will be available soon.

reference (*par*, *dx*)
For details, see `reference()`.

8.1.7 Sperical model and derivitives

class models.**sphericalCap3D_surfaceArea** (*varargin*)
Bases: `@geometricModel.geometricModel`

`sphericalCap3D_surfaceArea` describes the geometry of a spherical cap in 3D.

Geometric parameters:

- *surfaceArea*: (10^4 nm^2) the surface area of the spherical cap.
- *closeAngle*: (°) the angle from the pole to the edge of the cap.

Relavent biological structure:

- mammalian endocytic coat

Preview:

Note: It will be available soon.

reference (*par*, *dx*)
For details, see `reference()`.

getDerivedPars (*pars*)
For details, see `getDerivedPars()`.

class models.**sphericalCap3Dp_surfaceArea** (*varargin*)
Bases: `@parametricModel.parametricModel`

`sphericalCap3Dp_surfaceArea` describes a geometry of spherical cap in 3D. It describe the same geometry with the same parameterization as `sphericalCap3D_surfaceArea` but in a parametric form.

Geometric parameters:

- *surfaceArea*: (10^4 nm^2) the surface area of the spherical cap.
- *closeAngle*: (°) the angle from the pole to the edge of the cap.

Relavent biological structure:

- mammalian endocytic coat

See also:

`sphericalCap3D_surfaceArea`

Preview: See `sphericalCap3D_surfaceArea`

getDerivedPars (*pars*)
For details, see `getDerivedPars()`.

class models.**spheroid3Dp_surfaceArea**(*varargin*)

Bases: *models.spheroidCap3Dp_surfaceArea*

spheroid3Dp_surfaceArea describes the geometry of spheroid in 3D. It is parametric. Spheroid is a sphere flattened at the poles.

Important: Here the flattening is applied along the z-axis, not the y-axis.

Geometric parameters:

- ***surfaceArea*: (10^4 nm^2) the surface area of the spherical cap.**
  - *flattening*: (no unit) or *f*, is defined as *1-c/a*, where a and c are the two distinct axis lengths. c lines on the y-axis.

Relavent biological structure:

- deformed vesicle

See also:

spheroidCap3Dp_surfaceArea

Preview:

Note: It will be available soon.

getDerivedPars(*par*)

For details, see *getDerivedPars()*.

class models.**spheroidCap3Dp_surfaceArea**(*varargin*)

Bases: *@parametricModel.parametricModel*

sphericalCap3Dp_surfaceArea describes the geometry of spheroid cap in 3D. It is parametric. Spheroid cap is a spherial cap flattened at the poles.

Important: Here the flattening is applied along the y-axis, not the z-axis.

Geometric parameters:

- *surfaceArea*: (10^4 nm^2) the surface area of the spherical cap.
- ***closeAngle*: (°) the angle from the pole to the edge of the cap.**
  - *flattening*: (no unit) or *f*, is defined as *1-c/a*, where a and c are the two distinct axis lengths. c lines on the y-axis.

Relavent biological structure:

- mammalian endocytic coat

See also:

spheroid3Dp_surfaceArea

Preview:

> **Note:** It will be available soon.

`getDerivedPars`(*pars*)
For details, see `getDerivedPars()`.

8.1.8 2D projection of 3D geometry

class `models.hemispheroid2D`(*varargin*)
Bases: `@geometricModel.geometricModel`

`hemispheroid2D` describes the side-view projection of a hemispheroid.

Geometric parameters:

- *a*: (nm) the axis along the y-axis.
- *b*: (nm) the axis along the x-axis.
- *xcenter*: (nm) [obsolete] please set it to zero.
- *ycenter*: (nm) [obsolete] please set it to zero.

Relavent biological structure:

- actin network at the endocytic site

Preview:

> **Note:** It will be available soon.

`reference`(*par*, *dx*)
For details, see `reference()`.

class `models.thickRing2D`(*varargin*)
Bases: `@geometricModel.geometricModel`

`thickRing2D` describes the side-view projection of a thick ring.

Geometric parameters:

- *innerRadius*: (nm) the inner radius of the ring.
- *outerRadius*: (nm) the outer radius of the ring.
- *thickness*: (nm) the thickness of the ring.
- *xcenter*: (nm) [obsolete] please set it to zero.
- *ycenter*: (nm) [obsolete] please set it to zero.

Relavent biological structure:

- actin network at the endocytic site

Preview:

> **Note:** It will be available soon.

reference (*par*, *dx*)
For details, see `reference()`.

8.1.9 Random geometry

class models.**locsBG3D** (*varargin*)
Bases: `@geometricModel.geometricModel`

`locsBG3D` is usually used for simulations. It allows generating localizations in 3D at random positions.

Geometric parameters:

- *density*: (μm^{-2}) density of background localizations in the xy plane.
- *depth*: (nm) the depth of the ROI.

class models.**gaussianCluster2D** (*varargin*)
Bases: `@geometricModel.geometricModel`

`sphericalCap3Dp_surfaceArea` describes a geometry of spherical cap in 3D. It describe the same geometry with the same parameterization as `sphericalCap3D_surfaceArea` but in a parametric form.

Geometric parameters:

- *x0*: (nm) the x position of the cluster.
- *y0*: (nm) the y position of the cluster.

Important: The parameter sigma of the gaussian cluster is determined by the extrinsic parameter *variation*.

Relavent biological structure:

- a protein cluster on the plasma membrane

Preview:

Note: It will be available soon.

reference (*par*, *dx*)
For details, see `reference()`.

8.2 Fitter

This is the handle class definition.

class @LocMoFit.**LocMoFit** (*varargin*)
Bases: `matlab.mixin.Copyable`

This is the class for fitting a set of geometric models to SMLM data.

This class help user to set up and handle the model fitting.

If you would like to perform multi-step fitting, please create one SMLMModelFit object for each step.

Copy right: Yu-Le Wu, 2022

License: GPLv3

Version: 1.0.1

Pleas cite: Wu, Y.-L. et al. Maximum-likelihood model fitting for quantitative analysis of SMLM data. 2021.08.30.456756. bioRxiv (2021) doi:10.1101/2021.08.30.456756.

Last update: 25.07.2022

`dimension = None`
??? The dimension of the data.

`allParsArg = None`
All arguments of the parameters.

`parsInit = None`
???

`fitInfo = None`
Additional information acquired by fitting.

`externalInfo = '[]'`
External info, which were not acquired by the fit but rather used for alignment or so.

`model = None`
The model objects.

`solver = None`
The solver options.

`converterRules = None`
The defined conversion rules.

`converterUserDefined = None`
The temporary variables that the user defined.

`roiSize = '300'`
The size of region of interest in unit of nm.

`imgExtension = '0'`
The extention of image size than the roi size.

`dataDim = '3'`
The dimension of the data.

`allModelLayer = None`
The unique numbers of layers used by models.

`modelLayer = None`
The layer for the corresponding model.

`modelVerCascade = '1'`
For locs model only. Allow the user to specify different versions of the same model for different cascade steps.

`refPoint_spacing = '0.75'`
The spacing bwteen sampled ref points. In the unit of sigma.

`numOfLocsPerLayer = None`
The numbers of localizations per layer.

`representiveLocprec = None`
The representative localization precision.

`display = None`
Settings for displaying figures.

lutLayer = "'likelihood'"
The type of the objective function.

compensationFactor = '1'
The weights of layers.

fitterInfo = "'finished'"
can be 'initial', 'iterative' or 'finished'

advanceSetting = None
Advanced settings are defined here.

linkedGUI = None
If GUI is used, it will be saved here.

converterSource = None
The information source for the converter.

locs = None
the current parameters of the model being evaluated.

handles = None
handles to graphic objects

temp = None
all temp info is saved here.

numOfModel = None
The number of models used in this fitting step.

numOfLayer = None
The number of layers.

roiAreaVol = None
The area/volume of the roi;

sigmaCascade = None
The sigma factor for each step of the sequential cascade fit.

LocMoFit(*varargin*)
Construct the object of the class 'LocMoFit'

Usage: obj = LocMoFit(Name-value)

Parameters pairs (*Name-value*) –

- 'SolverName': one of 'fmincon', 'fminsearchbnd', and 'particleswarm'.
- 'SolverOptions':
- 'DataDim':
- 'TestLocs':

Returns *obj* – an LocMoFit object.

Note: Please create an *LocMoFit* object for each step of fitting.

See also:

setModel()

setModel(*model*, *modelId*)
Setting one single model of the LocMoFit object according to the modelId. Initiation of all arguments of the parameters (allParsArg).

Usage: setModel(obj,model,modelId)

Parameters

- **obj** – an LocMoFit object.
- **model** – an SMLMModel object or sub-object.
- **modelId** – the ID of the model being added.

addModel(*model*)
Add a specified model to the end of the list of models.

Usage: addModel(obj,model)

Parameters

- **obj** – an LocMoFit object.
- **model** – an SMLMModel object or sub-object.

changeModel(*newModel*, *modelNumber*)
Remove the old corresponding parameters and add a new model to overwrite the old model with the same ID.

Usage: changeModel(obj, newModel, modelNumber)

Parameters

- **obj** – an LocMoFit object.
- **newModel** – an SMLMModel object or sub-object being added.
- **modelNumberRemove** – the ID of the model being added.

rmLastModel()
Remove the last model (in terms of the order).

Usage: rmLastModel(obj)

Parameters **obj** – an LocMoFit object.

updateModel(*modelNumber*)
Respond to the change of any internal setting of the geometric model.

getImage(*modelNumber*, *varargin*)
Get an image of the specified model at the best parameters.

updateLayer()
Manage layer-dependent offsets.

prepFit()
Reshape arguments of fit pars: lb, ub, init(value), and min/max

viewPars()
Display the parsArg as a table.

exportPars (*modelID*, *type*)
Get parameters for a specific model.

— Syntax — pars = exportPars(obj, modelID, type)

— Description — pars: a structral array with parameter names as field names. obj: an LocMoFit object. modelID: the ID of the model where you want to get parameters. type: either 'lPar', 'mPar' or 'allPar', specifying the type of parameters you want to get.

wherePar (*parId*)
[Replaced] see getVariable(). 200731: this function has been replaced getVariable().

getVariable (*ID*)
Search for a variable in which info is potentially stored and report its location and value. ID shold look like par.m1.lPar.x or directly the variable name.

setParArg (*parId*, *varargin*)
parId looks like this: 'm1.lPar.x', where m1 means model 1, and x can be any name of the parameters

addPar (*parArg*)
Add a parameter. Usage:

obj.addPar(parArg)

Arg: *parArg: a cell in the order of 'model','type','name', 'lb','ub','value','fix','label','min' and 'max'

rmPar (*parId*)
Remove a parameter

getAllParId (*modelnumber*, *varargin*)
Export Id for all parameters given a model number Show all parId when modelnumber is not specified.

Usage: [parId,subParsArgTemp] = obj.getAllParId(modelnumber, varargin)

Parameters

- **modelnumber** – an LocMoFit object.
- **pairs** (`Name-value`) – 'form': either 'short', 'long', 'auxiliary'

Returns *modCoord* – reference coordinates.

convert2InteralLPar (*lPars*)
TODO: define the conversion for lPars in the furture Now this is just a placeholder

convert2InteralOffset (*offset*)
This function converts any valid offset to the interanl offset (weight)

getBGDensity ()
density here is defined as the projected density. this unit is locs/um^2

convertBG (*queriedForm*, *layer*, *val*)
convert from one parameterization to another.

getLocsInfo ()
Get locs counts per layer

lParSelector (*parameterType*, *parameterForm*)
Defines the form of parameters to be used It reacts when a certain form of lPar is selected.

initLParSelector (*modelnumber*, *isBackground*)
Initiates the form of parameters to be used

matchAllPar (*modelId*, *refObj*, *refModelId*, *varargin*)
matchAllPar() matches the parameters with the same names.

Uasage: obj.matchAllPar(refObj, modelId, except)

Parameters

- **obj** (*LocMoFit*) – an object created by *LocMoFit()*.
- **modelId** (*numeric scalar*) – the model ID that identifies the target model.
- **refObj** (*LocMoFit*) – an object created by *LocMoFit()*. The referece that the parameters matches
- **to.** –
- **refModelId** (*numeric scalar*) – the model ID that identifies the reference model.
- **pairs** (*Name-value*) –
  - except (character vector | cell array of character vectors): parameter IDs (parIds) of the parameters to exclude from the matching.

Returns Nothing.

Last update: 03.05.2022

reactToSet_advanceSetting ()
reactToSet_advanceSetting() defines how LoMoFit reacts when *advanceSetting* is set.

assignParsVal ()
In the given range, assign values to the parameters randomly.

Usage: obj.assignParsVal

Parameters **obj** – an LocMoFit object.

Returns Parameter values saved in obj.allParsArg.

getSimRef (*varargin*)
Get simulation reference based on the allArgVal.

Usage: modCoord = obj.getSimRef(Name-value)

Parameters

- **obj** – an LocMoFit object.
- **pairs** (*Name-value*) –
  - 'finalROISize': the final ROI size for fitting. the

  ROI size for simulations is usually larger than this value to make sure the background fills everywhere. * 'depth': the final depth. This option determines the axial range of the background.

Returns *modCoord* – reference coordinates.

getLLExpDist (*n*)
Get the labels of the model.

saveHandles (*pard*, *tag*)

if isempty(varargin) || rem(length(varargin),2)>0 error('Wrong pair(s) of field names and handles.')

end fn = varargin(1:2:end); h = varargin(2:2:end); for k = 1:length(fn)

obj.handles.(fn{k}) = h{k};

end

loglikelihoodFun (*fitPars*, *compensationFactor*, *locs*, *varargin*)
loglikelihoodFun computes the log-likelihood value of the fit.

getOFLL (*compensationFactor*)
get overfitted log-likelihood

getDerivedPars (*varargin*)
Get derived parameters of all (default) or a specific model. :param obj: an *LocMoFit* object.

Returns:

getModelInternalSettingList (*modelID*)
Get the list of the internal settings' name.

— Syntax — settings = getModelInternalSettingList(obj, modelInd).

— Description— settings: a list (string array) of the internal settings' name. obj: an LocMoFit object. modelID: the ID of the model.

setModelInternalSetting (*modelInd*, *setting*, *value*)
Set the value of a model internal settings.

getModelInternalSetting (*modelInd*, *setting*)
Get the value of a model internal settings.

updateVersion ()
This function is for necessary updates. Structral changes leading to failed runs have to be fixed here.

@LocMoFit.**fit** (*obj*, *locs*, *varargin*)
FIT Perform fitting based on the option values *fit()* performs fitting based on the options.

Usage: fit(obj, locs, varargin)

Parameters

- **obj** – a SMLMModelFit object.
- **locs** – a struct with fields of xnm, ynm, znm, locprecnm.
- **pairs** (*Name-value*) – *'locs2': a struct with fields of xnm, ynm, znm, locprecnm. This is the

second set of locs for particle fusion.

8.3 SMLM models

class @SMLMModel.**SMLMModel**
SMLMModel is a super-class for defining how to deal with a geometric model.

ParentObject = '[]'
Parental SMLMModelFit object.

ID = None
The model's ID in the parental SMLMModelFit object.

img = None
Model image.

parVal = None
[obsolete].

mPars = None
Model parameters.

modelObj = None
Source geometric model object.

modelFun = None
The function for creating coordinates based on the geometric model.

sourcePath = None
The path of the m file of the geometric model.

dimension = None
Dimension of the geometric model.

modelType = None
Type of the model, either discrete, discretized, continuous, intensity, or image.

fixSigma = 'false'
Fix the sigma to a specific value.

displayLut = "'red hot'"
The lookup table for the model.

layer = '1'
The layer that this model is fitted to.

addParent(*parent*)
Add the parental SMLMModelFit object.

exportMPars()
Export model parameters and their default values.

class `@functionModel.`**functionModel**(*model2load*)
A sub-class of `SMLMModel`. *functionModel* class handles any geometric model in the form of a function. *functionModel* handles the function differently based on its *modelType*. The *modelType* is per geometric model and defined in *modelType* of the `geometricModel`.

Last update: 14.10.2021

See also:

SMLMModel, *LocMoFit*, *geometricModel*

pixelSize = '5'
Pixel size of the model

sigma = '15'
Standard deviation of the gaussian kernel used for smoothing the model.

sigmaFactor = '1'
The scaling factor of the kernel's standard deviation.

samplingFactor = '0.75'
For continuous model, deciding the distance between ref points. In the unit of sigma. 0.75 means 0.75*sigma.

sigmaSet = None
The set of sigma.

sigmaZSet = None
The set of sigma in Z.

extraBlurr = None
This is a parameter determined by lPars.variation.

locsPrecFactor = None
The min sqrt(locprec^2+varation^2)

functionModel(*model2load*)
The constructor of the functional model object. This function fetches the default values from the geometric model.

updateMParsArg()
This function updates the mPars' arguments based on the change of the geometric model.

getPoint(*mPars*, *varargin*)
Getting sampled points from the model.

fun(*mPars*, *dx*)
convert the output of model for voxelblurr

deriveSigma(*locs*, *varargin*)
`deriveSigma()` derives the final sigma used for fitting. When `fixSigma` is set as true, sigmas are derived based on pre-defined values. Otherwise, sigmas are derived based on localization precisions. For a continuous model, the minimum sigma is defined as the median of localization precisions.

Uasage: obj.deriveSigma(locs)

Inputs:

- **obj** (`functionModel`) – an object created by `functionModel()`.
- **locs** (structure array) – a typical localization structure array used in SMAP.

Output:

- **sigmaFactor** (numeric vector) – a 1-by-2 vector that determines the fold of localization precisions used for fitting.
- **sigmaSet** (numeric vector | numeric scalar) – sigma used for fitting. A N-by-1 vector, where N is the number of localiztions when `fixSigma` is true.
- **sigmaZSet** (numeric vector | numeric scalar) – z sigma used for fitting. A N-by-1 vector, where N is the number of localiztions when `fixSigma` is true.

Last update: 28.04.2022

See also:

`functionModel`

class @imageModel.**imageModel**(*img*, *varargin*)

imageModel(*img*, *varargin*)
parse varargin

getImage(*mPars*, *varargin*)
parse varargin

8.4 Geometric model classes

These most basic classes allow defining a geometric model in user-defined forms (see `geometricModel`) or in a parametric form (see `parametricModel`).

8.4.1 Geometric model

Important: All the direct subclasses of `geometricModel` are required to contain the method `reference()`.

reference (*obj*, *par*, *dx*)

This funtion samples coordinates of the model as reference.

Usage: [model, p]= reference(obj, par, dx)

Input:

- **obj** (`geometricModel`) – an object of any subclass of `geometricModel`.
- **par** (structure array) – each field contains a parameter value and its fieldname should be the parameter name.
- **dx** (numeric scalar) – sampling rate.

Output:

- **model** (structure array) – a structure object. Its fieldnames are x, y, z, and n, indicating the amplitudes n at xyz positions of the sampled model points.
- **p** (structure array) – additional information of the model.

getDerivedPars (*obj*, *pars*)

This funtion calculates additional parameters derived from the geometric paramaters.

Usage: derivedPars = getDerivedPars(obj, pars)

Input:

- **obj** (`geometricModel`) – an object of any subclass of `geometricModel`.
- **pars** (structure array) – each field contains a parameter value and its fieldname should be the name of the geometric parameter.

Output:

- **derivedPars** (structure array) – each field contains a parameter value and its fieldname should be the name of the derived parameter.

class @geometricModel.**geometricModel** (*varargin*)

`geometricModel` is the superclass of any geometric model. It contains methods for building own geometric models.

Last update: 14.10.2021

name = None

names of model parameters

fix = None

fixing model parameters

value = None

values of model parameters

lb = None
relative lower bounds of model parameters

ub = None
relative upper bounds of model parameters

min = None
min values of model parameters

max = None
max values of model parameters

internalSettings = None
parameters that do not suit fitting

modelType = None
selected model type

modelTypeOption = None
possible model types of a specific geometric model

dimension = None
a scalar indicating the dimensionality of the model. Either 2 or 3.

listed = 'false'
whether this model will be listed in the GUI.

geometricModel(*varargin*)

Usage: obj = geometricModel(varargin)

Parameters

- **pairs** (*Name-value*) –
- **'Parent'** (***) – parental obj.

Returns *obj* – a `geometricModel` object.

getThings2Plot(*mPar*)
The user can define what should be also displayed in the plots. :param obj: a `geometricModel` object.

Returns *items* – things to be plotted.

getDerivedPars(*varargin*)
Get derived parameters of the current model. :param obj: an `functionModel` object.

Returns *derivedPars* – derived parameters.

8.4.2 Parametric Model

class @parametricModel.**parametricModel**(*varargin*)
`parametricModel` is a subclass of *geometricModel*. This class allows the user to define their model in a parametric manner.

reference(*par*, *dx*)
Please do not modify this part. If you have any requst regarding this part, please contact us.

convertPar(*par*)
Define the internal conversion of parameters. Skip this part if no conversion is necessary.

8.5 Indices and tables

- genindex
- modindex
- search

MATLAB MODULE INDEX

@

@functionModel, 94
@geometricModel, 96
@imageModel, 95
@LocMoFit, 87
@parametricModel, 97
@SMLMModel, 93

m

models, 77

INDEX

Non-alphabetical

@functionModel (*module*), 94
@geometricModel (*module*), 96
@imageModel (*module*), 95
@LocMoFit (*module*), 87
@parametricModel (*module*), 97
@SMLMModel (*module*), 93

A

addModel() (*@LocMoFit.LocMoFit method*), 90
addPar() (*@LocMoFit.LocMoFit method*), 91
addParent() (*@SMLMModel.SMLMModel method*), 94
advanceSetting (*@LocMoFit.LocMoFit attribute*), 89
allModelLayer (*@LocMoFit.LocMoFit attribute*), 88
allParsArg (*@LocMoFit.LocMoFit attribute*), 88
arc2D (*class in models*), 77
arc2D_arcLen (*class in models*), 78
assignParsVal() (*@LocMoFit.LocMoFit method*), 92

B

bucket2D (*class in models*), 78

C

changeModel() (*@LocMoFit.LocMoFit method*), 90
compensationFactor (*@LocMoFit.LocMoFit attribute*), 89
convert2InteralLPar() (*@LocMoFit.LocMoFit method*), 91
convert2InteralOffset() (*@LocMoFit.LocMoFit method*), 91
convertBG() (*@LocMoFit.LocMoFit method*), 91
converterRules (*@LocMoFit.LocMoFit attribute*), 88
converterSource (*@LocMoFit.LocMoFit attribute*), 89
converterUserDefined (*@LocMoFit.LocMoFit attribute*), 88
convertPar() (*@parametricModel.parametricModel method*), 97
cspline3D_midPoint (*class in models*), 80
csplineClosedTube3D_midPoint (*class in models*), 80
csplineTube3D_midPoint (*class in models*), 81
csplineTube3D_xyz (*class in models*), 81

D

dataDim (*@LocMoFit.LocMoFit attribute*), 88
deriveSigma() (*@functionModel.functionModel method*), 95
dimension (*@geometricModel.geometricModel attribute*), 97
dimension (*@LocMoFit.LocMoFit attribute*), 88
dimension (*@SMLMModel.SMLMModel attribute*), 94
display (*@LocMoFit.LocMoFit attribute*), 88
displayLut (*@SMLMModel.SMLMModel attribute*), 94
dualEllipse3D_avgR_discrete (*class in models*), 83
dualEllipse3D_discrete (*class in models*), 82
dualRing3D_discrete (*class in models*), 83

E

ellipse3D (*class in models*), 79
exportMPars() (*@SMLMModel.SMLMModel method*), 94
exportPars() (*@LocMoFit.LocMoFit method*), 90
externalInfo (*@LocMoFit.LocMoFit attribute*), 88
extraBlurr (*@functionModel.functionModel attribute*), 95

F

fit() (*in module @LocMoFit*), 93
fitInfo (*@LocMoFit.LocMoFit attribute*), 88
fitterInfo (*@LocMoFit.LocMoFit attribute*), 89
fix (*@geometricModel.geometricModel attribute*), 96
fixSigma (*@SMLMModel.SMLMModel attribute*), 94
fun() (*@functionModel.functionModel method*), 95
functionModel (*class in @functionModel*), 94
functionModel() (*@functionModel.functionModel method*), 95

G

gaussianCluster2D (*class in models*), 87
geometricModel (*class in @geometricModel*), 96
geometricModel() (*@geometricModel.geometricModel method*), 97

getAllParId() (@LocMoFit.LocMoFit method), 91
getBGDensity() (@LocMoFit.LocMoFit method), 91
getDerivedPars(), 96
getDerivedPars() (@geometricModel.geometricModel method), 97
getDerivedPars() (@LocMoFit.LocMoFit method), 93
getDerivedPars() (models.bucket2D method), 78
getDerivedPars() (models.csplineClosedTube3D_midPoint method), 81
getDerivedPars() (models.csplineTube3D_midPoint method), 81
getDerivedPars() (models.sphericalCap3D_surfaceArea method), 84
getDerivedPars() (models.sphericalCap3Dp_surfaceArea method), 84
getDerivedPars() (models.spheroid3Dp_surfaceArea method), 85
getDerivedPars() (models.spheroidCap3Dp_surfaceArea method), 86
getImage() (@imageModel.imageModel method), 95
getImage() (@LocMoFit.LocMoFit method), 90
getLLExpDist() (@LocMoFit.LocMoFit method), 92
getLocsInfo() (@LocMoFit.LocMoFit method), 91
getModelInternalSetting() (@LocMoFit.LocMoFit method), 93
getModelInternalSettingList() (@LocMoFit.LocMoFit method), 93
getOFLL() (@LocMoFit.LocMoFit method), 93
getPoint() (@functionModel.functionModel method), 95
getSimRef() (@LocMoFit.LocMoFit method), 92
getThings2Plot() (@geometricModel.geometricModel method), 97
getThings2Plot() (models.cspline3D_midPoint method), 80
getThings2Plot() (models.csplineClosedTube3D_midPoint method), 81
getThings2Plot() (models.csplineTube3D_midPoint method), 81
getThings2Plot() (models.csplineTube3D_xyz method), 82
getVariable() (@LocMoFit.LocMoFit method), 91

H

handles (@LocMoFit.LocMoFit attribute), 89
hemispheroid2D (class in models), 86

I

ID (@SMLMModel.SMLMModel attribute), 93
imageModel (class in @imageModel), 95
imageModel() (@imageModel.imageModel method), 95
img (@SMLMModel.SMLMModel attribute), 93
imgExtension (@LocMoFit.LocMoFit attribute), 88
initLParSelector() (@LocMoFit.LocMoFit method), 91
internalSettings (@geometricModel.geometricModel attribute), 97

L

layer (@SMLMModel.SMLMModel attribute), 94
lb (@geometricModel.geometricModel attribute), 96
linkedGUI (@LocMoFit.LocMoFit attribute), 89
listed (@geometricModel.geometricModel attribute), 97
LocMoFit (class in @LocMoFit), 87
LocMoFit() (@LocMoFit.LocMoFit method), 89
locs (@LocMoFit.LocMoFit attribute), 89
locsBG3D (class in models), 87
locsPrecFactor (@functionModel.functionModel attribute), 95
loglikelihoodFun() (@LocMoFit.LocMoFit method), 93
lParSelector() (@LocMoFit.LocMoFit method), 91
lutLayer (@LocMoFit.LocMoFit attribute), 88

M

matchAllPar() (@LocMoFit.LocMoFit method), 91
max (@geometricModel.geometricModel attribute), 97
min (@geometricModel.geometricModel attribute), 97
model (@LocMoFit.LocMoFit attribute), 88
modelFun (@SMLMModel.SMLMModel attribute), 94
modelLayer (@LocMoFit.LocMoFit attribute), 88
modelObj (@SMLMModel.SMLMModel attribute), 94
models (module), 77
modelType (@geometricModel.geometricModel attribute), 97
modelType (@SMLMModel.SMLMModel attribute), 94
modelTypeOption (@geometricModel.geometricModel attribute), 97
modelVerCascade (@LocMoFit.LocMoFit attribute), 88
mPars (@SMLMModel.SMLMModel attribute), 94

N

name (@geometricModel.geometricModel attribute), 96
numOfLayer (@LocMoFit.LocMoFit attribute), 89
numOfLocsPerLayer (@LocMoFit.LocMoFit attribute), 88
numOfModel (@LocMoFit.LocMoFit attribute), 89

P

parametricModel (class in @parametricModel), 97

ParentObject (*@SMLMModel.SMLMModel attribute*), 93
parsInit (*@LocMoFit.LocMoFit attribute*), 88
parVal (*@SMLMModel.SMLMModel attribute*), 94
pixelSize (*@functionModel.functionModel attribute*), 94
prepFit() (*@LocMoFit.LocMoFit method*), 90

R

reactToSet_advanceSetting() (*@LocMoFit.LocMoFit method*), 92
reference(), 96
reference() (*@parametricModel.parametricModel method*), 97
reference() (*models.arc2D method*), 78
reference() (*models.arc2D_arcLen method*), 78
reference() (*models.bucket2D method*), 78
reference() (*models.cspline3D_midPoint method*), 80
reference() (*models.csplineClosedTube3D_midPoint method*), 81
reference() (*models.csplineTube3D_midPoint method*), 81
reference() (*models.csplineTube3D_xyz method*), 82
reference() (*models.dualEllipse3D_avgR_discrete method*), 83
reference() (*models.dualEllipse3D_discrete method*), 83
reference() (*models.dualRing3D_discrete method*), 84
reference() (*models.ellipse3D method*), 79
reference() (*models.gaussianCluster2D method*), 87
reference() (*models.hemispheroid2D method*), 86
reference() (*models.ring2D method*), 79
reference() (*models.ring3D method*), 79
reference() (*models.sphericalCap3D_surfaceArea method*), 84
reference() (*models.thickRing2D method*), 86
refPoint_spacing (*@LocMoFit.LocMoFit attribute*), 88
representiveLocprec (*@LocMoFit.LocMoFit attribute*), 88
ring2D (*class in models*), 79
ring3D (*class in models*), 79
rmLastModel() (*@LocMoFit.LocMoFit method*), 90
rmPar() (*@LocMoFit.LocMoFit method*), 91
roiAreaVol (*@LocMoFit.LocMoFit attribute*), 89
roiSize (*@LocMoFit.LocMoFit attribute*), 88

S

samplingFactor (*@functionModel.functionModel attribute*), 94
saveHandles() (*@LocMoFit.LocMoFit method*), 92
setModel() (*@LocMoFit.LocMoFit method*), 89
setModelInternalSetting() (*@LocMoFit.LocMoFit method*), 93
setParArg() (*@LocMoFit.LocMoFit method*), 91
sigma (*@functionModel.functionModel attribute*), 94
sigmaCascade (*@LocMoFit.LocMoFit attribute*), 89
sigmaFactor (*@functionModel.functionModel attribute*), 94
sigmaSet (*@functionModel.functionModel attribute*), 94
sigmaZSet (*@functionModel.functionModel attribute*), 94
SMLMModel (*class in @SMLMModel*), 93
solver (*@LocMoFit.LocMoFit attribute*), 88
sourcePath (*@SMLMModel.SMLMModel attribute*), 94
sphericalCap3D_surfaceArea (*class in models*), 84
sphericalCap3Dp_surfaceArea (*class in models*), 84
spheroid3Dp_surfaceArea (*class in models*), 84
spheroidCap3Dp_surfaceArea (*class in models*), 85

T

temp (*@LocMoFit.LocMoFit attribute*), 89
thickRing2D (*class in models*), 86

U

ub (*@geometricModel.geometricModel attribute*), 97
updateLayer() (*@LocMoFit.LocMoFit method*), 90
updateModel() (*@LocMoFit.LocMoFit method*), 90
updateMParsArg() (*@functionModel.functionModel method*), 95
updateVersion() (*@LocMoFit.LocMoFit method*), 93

V

value (*@geometricModel.geometricModel attribute*), 96
viewPars() (*@LocMoFit.LocMoFit method*), 90

W

wherePar() (*@LocMoFit.LocMoFit method*), 91
