# Supplementary figures and images for: Maximum-likelihood model fitting for quantitative analysis of SMLM data
Source: Nat Methods. 2022 Dec 15;20(1):139–48. doi: 10.1038/s41592-022-01676-z (PMC9834062; doi:10.1038/s41592-022-01676-z)

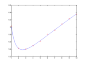

Supplement: Supplementary file 7 — Source code of LocMoFit v1.1 [file 41592_2022_1676_MOESM7_ESM.zip › LocMoFit/external/PolyfitnTools/demo/html/polyfitn_demo.png]

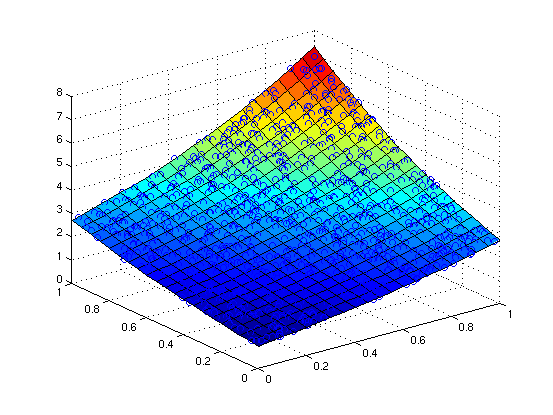

Supplement: Supplementary file 7 — Source code of LocMoFit v1.1 [file 41592_2022_1676_MOESM7_ESM.zip › LocMoFit/external/PolyfitnTools/demo/html/polyfitn_demo_01.png]

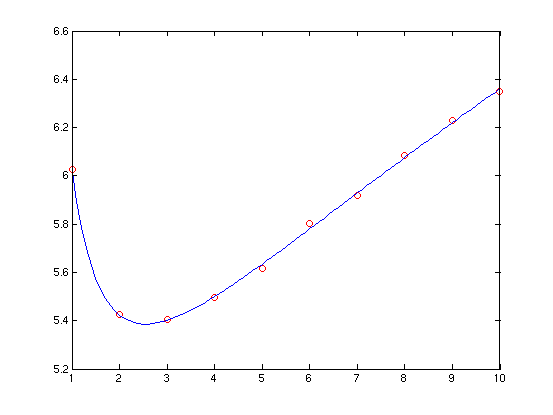

Supplement: Supplementary file 7 — Source code of LocMoFit v1.1 [file 41592_2022_1676_MOESM7_ESM.zip › LocMoFit/external/PolyfitnTools/demo/html/polyfitn_demo_02.png]
